# Designing Single‐Atom Active Sites on sp^2^‐Carbon Linked Covalent Organic Frameworks to Induce Bacterial Ferroptosis‐Like for Robust Anti‐Infection Therapy

**DOI:** 10.1002/advs.202207507

**Published:** 2023-02-27

**Authors:** Baohong Sun, Xinye Wang, Ziqiu Ye, Juyang Zhang, Xiong Chen, Ninglin Zhou, Ming Zhang, Cheng Yao, Fan Wu, Jian Shen

**Affiliations:** ^1^ National and Local Joint Engineering Research Center of Biomedical Functional Materials School of Chemistry and Materials Science Nanjing Normal University Nanjing 210023 P. R. China; ^2^ School of Chemistry and Molecular Engineering Nanjing Tech University Nanjing 211816 P. R. China; ^3^ Key Laboratory of Cardiovascular and Cerebrovascular Medicine School of Pharmacy Nanjing Medical University Nanjing 211166 P. R. China; ^4^ Jiangsu Engineering Research Center of Interfacial Chemistry Nanjing University Nanjing 210023 P. R. China

**Keywords:** anti‐infection therapy, covalent organic frameworks, ferroptosis‐like, single‐atom catalysts, transition metal complex

## Abstract

With the threat posed by drug‐resistant pathogenic bacteria, developing non‐antibiotic strategies for eradicating clinically prevalent superbugs remains challenging. Ferroptosis is a newly discovered form of regulated cell death that can overcome drug resistance. Emerging evidence shows the potential of triggering ferroptosis‐like for antibacterial therapy, but the direct delivery of iron species is inefficient and may cause detrimental effects. Herein, an effective strategy to induce bacterial nonferrous ferroptosis‐like by coordinating single‐atom metal sites (e.g., Ir and Ru) into the sp^2^‐carbon‐linked covalent organic framework (sp^2^c‐COF‐Ir‐ppy_2_ and sp^2^c‐COF‐Ru‐bpy_2_) is reported. Upon activating by light irradiation or hydrogen peroxide, the as‐constructed Ir and Ru single‐atom catalysts (SACs) can significantly expedite intracellular reactive oxygen species burst, enhance glutathione depletion‐related glutathione peroxidase 4 deactivation, and disturb the nitrogen and respiratory metabolisms, leading to lipid peroxidation‐driven ferroptotic damage. Both SAC inducers show potent antibacterial activity against Gram‐positive bacteria, Gram‐negative bacteria, clinically isolated methicillin‐resistant *Staphylococcus aureus* (MRSA), and biofilms, as well as excellent biocompatibility and strong therapeutic and preventive potential in MRSA‐infected wounds and abscesses. This delicate nonferrous ferroptosis‐like strategy may open up new insights into the therapy of drug‐resistant pathogen infection.

## Graphical abstract

A novel nonferrous ferroptosis‐like strategy by coordinating single‐atom metal sites (e.g., Ir and Ru) into the sp^2^‐carbon‐linked covalent organic framework is reported. The as‐constructed Ir and Ru sites can effectively induce lipid peroxidation‐driven bacterial ferroptotic damage, showing strong therapeutic and preventive potential in infected wounds and abscesses.

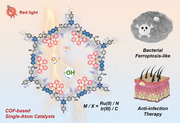

## 1 Introduction

Antimicrobial resistance severely compromises traditional chemotherapy regimens and persists as a worldwide health problem.^[^ ^1^ ^]^ Bacteria can employ a variety of strategies to avert growth suppression of conventional antibiotic therapy, consisting of enzyme inactivation and target modification.^[^ ^2^ ^]^ Furthermore, they could spark post‐antibiotic dilation and recurrent/persistent infection through reprogramming host metabolism, interfering with degradation pathways, and inhibiting immune cells.^[^ ^3^ ^]^ Alternative strategies capable of circumventing antibiotic resistance are of utmost importance. Ferroptosis is an iron‐dependent form of cell death resulting from lipid peroxidation (LPO) that has been implicated in various biological contexts, from development to aging to immunity and cancer.^[^ ^4^ ^]^ Several types of strategies have been described to induce ferroptosis to date, comprising iron delivery,^[^ ^5^ ^]^ system Xc^−^ suppression,^[^ ^6^ ^]^ glutathione (GSH) depletion, and glutathione peroxidase 4 (GPX4) inhibition.^[^ ^7^ ^]^ The precise mechanism that eventually results in ferroptotic cell death probably involves damage to membrane integrity, disruption of membrane properties through lipid cross‐linking, and further oxidative impairment to macromolecules and cellular structures induced by reactive oxygen species (ROS) derived from polyunsaturated fatty acid chains.^[^ ^8^ ^]^ It has been hypothesized that the regulation of key molecules in classical regulatory pathways of ferroptosis can serve as a potential approach to overcoming drug resistance.^[^ ^9^ ^]^

Emerging ferrous‐based nanomaterials, comprising ferumoxytol, nano‐iron sulfides, and iron–organic frameworks, have been applied as inducers for ferroptosis benefiting from the Fenton reaction impelled by Fe^2+^.^[^ ^10^ ^]^ For instance, attempts have been made to introduce iron ions into the metastable Fe_3_S_4_ (greigite) or FeSO_4_ to facilitate the iron overload‐triggered GSH consumption, leading to the ferroptosis‐like of bacterial cells.^[^ ^11^ ^]^ Ferroferric oxide‐based nanoassemblies were also utilized as the inducers for bacterial ferroptosis‐like death by eliciting intracellular iron overload and iron metabolism interference.^[^ ^12^ ^]^ However, these current iron‐based nanomaterials used to induce bacterial ferroptosis‐like are far from satisfactory, requiring very high Fe doses or supplementary ingredients to achieve combinatorial effects in general.^[^ ^10b^ ^]^ Alternatively, the direct delivery of iron species may cause detrimental effects such as neurovirulence, oxidative stress, and anaphylactic reactions in normal tissues.^[^ ^13^ ^]^ Single‐atom catalysts (SACs) have become an exciting frontier recently in chemical catalysis because of their precisely identified active centers, robust catalytic performances, and high stability.^[^ ^14^ ^]^ SACs can be regarded as the extreme limit of the precise design of nanocatalytic materials at the atomic level. Particularly, they have been utilized as bio‐inspired nanozymes to mimic natural enzymes’ structure and excellent catalytic ability, efficiently generating excessive ROS for bacterial or tumor inhibition.^[^ ^15^ ^]^ For example, Qu and colleagues report a self‐adapting iron‐based SAC to accelerate selective and safe ferroptosis;^[^ ^16^ ^]^ a nonferrous‐based Pd‐SAC with simulated activities of double peroxidase (POD) and glutathione oxidase (GSHOx) also efficiently induces ferroptosis characterized by upregulation of LPO and ROS.^[^ ^17^ ^]^ Unfortunately, considering the low intracellular H_2_O_2_ level of bacterial cells, Fenton reaction alone is difficult to generate sufficient ROS, which weakens the catalytic therapeutic efficacy of conventional SACs.^[^ ^18^ ^]^

Recently, isolated active metal centers anchored to solid support represent an innovative breakthrough in photochemistry.^[^ ^19^ ^]^ Nanoscale covalent organic frameworks (COFs) composed of suitable building blocks and organic functional groups have emerged as highly promising carriers for their tunable microstructures and optically electrical properties in preference to traditional catalyst supports.^[^ ^20^ ^]^ The possibility of utilizing COFs as supporting materials to construct SACs to meet the requirements of ferroptosis was investigated. Various monatomic metal centers anchored on COFs could present effective photocatalysis.^[^ ^21^ ^]^ Many studies have certified that transition‐metal elements such as Ir and Ru could act as single‐atom active sites without disrupting the framework for constructing high‐performance photochemical catalysts.^[^ ^22^ ^]^ Adding single transition metal atoms to the bare photocatalysts can expand the optical response range, shorten the electron transfer distance, and form stable intermediate configurations in photocatalytic reactions by virtue of the increased delocalization effect, enduing SACs with excellent photocatalytic performance.^[^ ^21^, ^23^ ^]^ Transition metal SACs have also been reported to exhibit POD and GSHOx activity.^[^ ^24^ ^]^ Hence, exploring a COF‐based SAC paradigm for bacterial ferroptosis‐inducing agents is imperative and highly desirable.

**Scheme 1:**
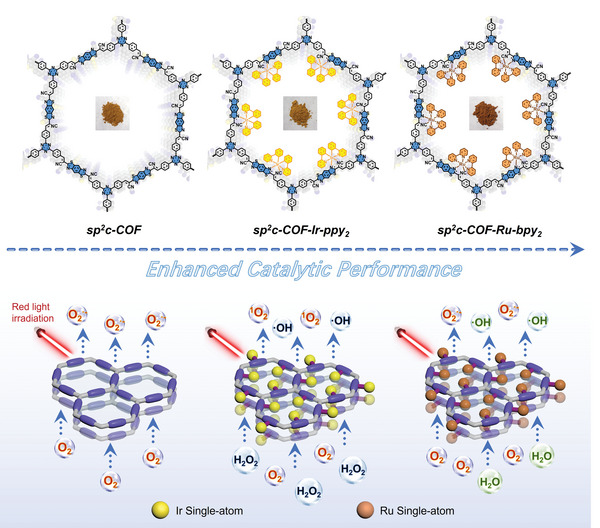
Scheme depicting coordinative synthesis of Ir and Ru SACs in the pore of sp^2^c‐COF.

Herein, we prepare two types of monatomic transition metal sites (e.g., Ir and Ru) anchored on sp^2^c‐linked COF (sp^2^c‐COF) skeletons with a metal–nitrogen–carbon bridging structure (**Scheme** **1**). Facilitated by covalent interactions in the Schiff base reaction, methoxy polyethylene glycol amine (mPEG‐NH_2_‐4000) polymer could be coated to produce the hydrophilic and highly biocompatible SACs (sp^2^c‐COF‐Ir‐ppy_2_ and sp^2^c‐COF‐Ru‐bpy_2_). The experimental results and density functional theory (DFT) calculation indicated that the excellent photocatalytic capacity and POD activity of Ir and Ru SACs were attributed to the intrinsic porous properties of the COF and the synergistic effect between atomically dispersed metal centers and sp^2^c‐COF hosts. Upon irradiation, the Ir and Ru active sites could cause the production of suprathreshold ROS, the consumption of intracellular GSH, and the disturbance of respiratory chain and metabolism together facilitating irreversible LPO‐driven ferroptosis‐like pathways. Both inducers show low hemolysis and cytotoxicity, as well as potent antibacterial activity against various bacteria, drug‐resistance bacteria, and strong therapeutic and preventive potential for methicillin‐resistant *Staphylococcus aureus* (MRSA)‐induced infections in the wound and abscess models. Collectively, we conducted a proof‐of‐concept study to discover COF‐based SAC as an antibacterial ferroptosis‐like initiator to eliminate infections.

## 2 Results and Discussion

### 2.1 Synthesis and Characterization

**Figure 1:**
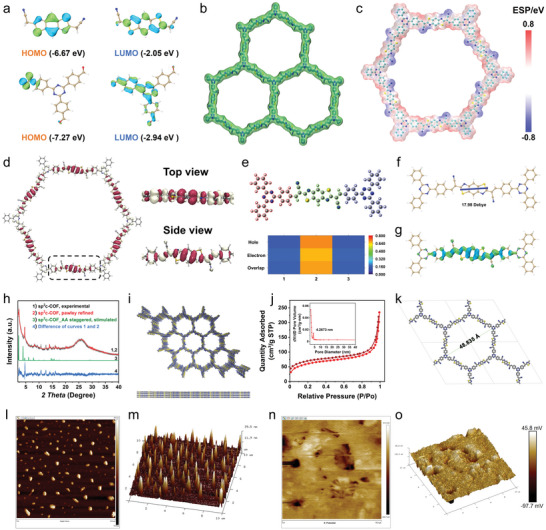
Characterizations of sp^2^c‐COF. a) HOMO and LUMO of BTHAN and TA. b) Extended structures of non‐interpenetrated sp^2^c‐COF. c) Electrostatic potential surface of sp^2^c‐COF repetitive units showing possible active sites. d) The charge density differences of sp^2^c‐COF and its magnified top and side views, with magenta and yellow representing electron accumulation and depletion, respectively. e) Hole and electron distribution heat map in the excited state of sp^2^c‐COF fragment. f) The direction of the dipole moment produced by the local polarization of sp^2^c‐COF. g) The electron–hole distributions of sp^2^c‐COF in the excited state, with blue and green representing electron accumulation and depletion, respectively. h) The Pawley refinement of sp^2^c‐COF: the experimental XRD pattern is shown in black, the Pawley refinement pattern in red, their difference in blue, and the simulated pattern using AA stacking mode in green. i) Crystal structures of the sp^2^c‐COF AA stacking model. j) N_2_ adsorption–desorption isotherms for sp^2^c‐COF at 77 K. Inset: pore‐size distribution calculated by fitting the NLDFT model to adsorption data. k) The simulation pore size of sp^2^c‐COF in AA stacking. l,m) AFM image and its 3D topography image of sp^2^c‐COF. n,o) Surface potential image and its 3D potential lines profile of sp^2^c‐COF.

Substantial attention has recently been focused on the olefin‐based COFs with fully *π*‐conjugated systems, a new class of promising semiconductor materials.^[^ ^25^ ^]^ The robust C=C bond not only endues the framework with excellent stability under harsh conditions but also ensures efficient electron transfer via extensive *π*‐conjugation throughout the framework.^[^ ^26^ ^]^ Nevertheless, due to the poor reversibility of C=C bond formation, the preparation of sp^2^‐carbon conjugated COFs maintains a tremendous challenge. Fortunately, under solvothermal conditions (tetrahydrofuran/0.1 m Cs_2_CO_3_ = 20/1 v/v, 3 days, 120 °C), the topology‐directed polycondensation of *C*_3_‐symmetric 4,4′,4″‐(1,3,5‐triazine‐2,4,6‐triyl)tribenzaldehyde (TA) as a knote and *C*_2_‐symmetric linear 2,6‐dicyanomethylbenzo[1,2‐d:4,5‐d′]bisthiazole (BTHAN) as a linker yielded a sp^2^c‐COF (Figures S1–S4, Supporting Information). The establishment of a donor–acceptor (D–A) conjugated system in COF by covalent linking of sulfur‐containing aromatic heterocyclic sites with triazine active sites would be an effective strategy to enhance intramolecular charge transfer capability to promote *π*‐electron delocalization, resulting in a narrower bandgap and desirable highest occupied molecular orbital (HOMO) and lowest unoccupied molecular orbital (LUMO) energy levels.^[^ ^27^ ^]^ This push–pull effect was demonstrated by calculating the molecular orbital density of the ligand by Gaussian at the B3LYP/6‐31G* theoretical level (**Figure** **1**a). The HOMO orbitals of BTHAN are mainly concentrated on the benzothiazole portion, while the LUMO is delocalized mainly over the triazine units, suggesting a much higher degree of LUMO–HOMO separation, leading to more efficient charge separation.

For the observation and comparison of local polarization and charge separation behavior of the synthesized COF molecules, we first exploited DFT to study the optimized molecular structures (Figure 1b,c). Noticeably, the electrostatic potential analysis for sp^2^c‐COF exhibits a positive distribution mainly on TA units, while the negative regions are mainly located on the cyanide group of the BTHAN portion, indicating that the positive and negative charges were obviously separated due to the effect of polarization on the local charge density triggered by the D–A characteristics of the backbones.^[^ ^28^ ^]^ Afterward, the charge separation ability of COFs in ground state and excited state was investigated. First, the benzothiazole group was recognized as an electron accumulator of sp^2^c‐COF in the ground‐state charge density differences (Figure 1d), yet the electrons in the TA group were depleted, indicating an uneven charge distribution. Thereafter, BTHAN can create more holes and electrons according to the heat map for sp^2^c‐COF fragments (Figure 1e). In addition, the dipole moment of the TA to BTHAN molecule was calculated as 17.98 Debye, demonstrating the presence of local charge polarization (Figure 1f). The excited‐state charge‐separation behavior of sp^2^c‐COF was analyzed with the electron–hole distribution, in which an obvious spatial charge separation was examined (Figure 1g). To sum up, these results demonstrate that the local polarization in a single sp^2^c‐COF molecule can lead to significant charge separation.

The powder X‐ray diffraction (PXRD) measurement exhibits three distinct peaks at 2.390, 4.633, and 21.817, indexed as the (100), (200), and (001) reflections, respectively (Figure 1h, black curve). The optimization for the 2D monolayer conformation and configuration of different stacking models has been conducted via the density function‐based tight binding (DFTB) method. The AA‐stacking model with the most favorable energy was obtained and the yielded PXRD pattern (Figure 1h, green curve) is consistent with the profile examined in the experiment. The Pawley‐refined PXRD pattern (Figure 1h, red curve) with the space group *P6*/*m* and unit cell parameters of *a* = *b* = 44.6378 Å, *c* = 3.5311 Å, and *c*/*a* = 0.0791 reproduced the curve observed in the experiment with negligible differences (Figure 1h, blue curve). Table S1 of the Supporting Information summarizes atomic atomistic coordinates generated by DFTB calculation and Pawley refinement, respectively. Hence, the reconstructed sp^2^c‐COF reveals an extended hexagonal 2D lattice with sp^2^ carbon skeleton along the *x* and *y* directions (Figure 1i). The existence of the (001) plane at 21.817° indicates the structural order of 3.5 Å separation in the *z* direction perpendicular to the 2D sheet.

Fourier transform infrared (FT IR) spectroscopy disclosed that for both BTHAN monomer and sp^2^c‐COF, the cyano group exhibits a stretching vibrational peak at 2248 cm^−1^ (Figure S5, Supporting Information). A peak at 1704 cm^−1^ ascribed to the C=O stretching vibration was observed in monomer TA and was widely attenuated in sp^2^c‐COF, suggesting that there was a high degree of polymerization in the skeleton. The newly formed peak at 3047 cm^−1^ in sp^2^c‐COF can be attributed to CH=C stretching, which clearly indicated the C=C connection in the skeleton. Solid‐state ^13^C cross‐polarization magic‐angle spinning nuclear magnetic resonance (^13^C CP‐MAS NMR) spectroscopy showed a peak at 163 ppm, confirming the presence of a thiazole ring (Figure S6, Supporting Information). Peaks of ≈105 and 115 ppm further supported the formation of vinylene bonds and the presence of cyano units.^[^ ^29^ ^]^ These remarkable features manifested the successful condensation of the monomers. Nitrogen adsorption–desorption tests were carried out at 77 K and the characteristic type I curves have been recognized in the simulated shape diagram (Figure 1j). At low relative pressure, gas absorption increases sharply (*P*/*P*_0_ < 0.1), which indicates the presence of micropores. The Brunauer–Emmett–Teller surface area was calculated to be 209.5 m^2^ g^−1^. The average aperture obtained by DFT fitting is ≈4.2673 nm, which corresponds to the theoretical value (4.8835 nm) in AA models (Figure 1k). AFM displayed that the surface roughness of sp^2^c‐COF is around tens of nanometers (Figure 1l,m). The significant difference in the surface potential of the sp^2^c‐COF layer in vertical‐contact mode demonstrates remarkable local polarization‐induced charge separation characteristics (Figure 1n,o).^[^ ^30^ ^]^ Finally, thermogravimetric analysis (TGA) suggested that sp^2^c‐COF performs excellent thermal stability, even with a residual carbon content of more than 50% up to 800 °C (Figure S7, Supporting Information).

**Figure 2:**
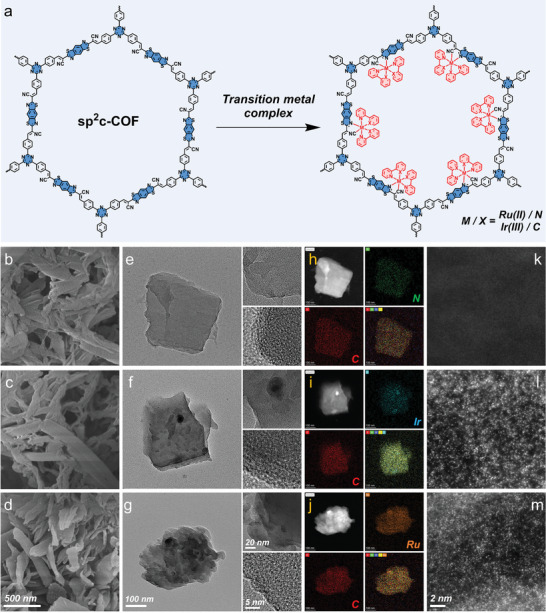
Morphologies of Ir and Ru SACs. a) Synthetic route of sp^2^c‐COF‐Ir‐ppy_2_ and sp^2^c‐COF‐Ru‐bpy_2_. b–d) FESEM images of b) sp^2^c‐COF, c) sp^2^c‐COF‐Ir‐ppy_2_, d) and sp^2^c‐COF‐Ru‐bpy_2_. e–g) TEM and HRTEM images of e) sp^2^c‐COF, f) sp^2^c‐COF‐Ir‐ppy_2_, g) and sp^2^c‐COF‐Ru‐bpy_2_. h–j) TEM‐EDX mapping images of C, N, Ir, and Ru elements in selected areas of h) sp^2^c‐COF, i) sp^2^c‐COF‐Ir‐ppy_2_, j) and sp^2^c‐COF‐Ru‐bpy_2_ (dark field mode). k–m) SAC‐HAADF‐STEM images of k) sp^2^c‐COF, l) sp^2^c‐COF‐Ir‐ppy_2_, and m) sp^2^c‐COF‐Ru‐bpy_2_.

Postsynthetic metalation of sp^2^c‐COF involved dispersing it in dichloromethane or dichloromethane/methanol in the presence of dimer [Ir_2_(ppy)_4_Cl_2_] and dimer [Ru_2_(bpy)_2_Cl_2_] (**Figure** **2**a). For this metalation, dimeric transition complexes were chosen as iridium and ruthenium sources since they are capable of binding powerful photoredox complexes through coordination with the thiazole and cyanogen ligands present in sp^2^c‐COF.^[^ ^31^ ^]^ The content of Ir and Ru incorporated into the metalized sp^2^c‐COF (sp^2^c‐COF‐Ir‐ppy_2_ and sp^2^c‐COF‐Ru‐bpy_2_) was analyzed with an inductively coupled plasma optical emission spectrometer (ICP‐OES). The contents of Ir and Ru in COFs were 5.37 and 2.12 wt%, respectively. These values imply that 31.6% of the thiazole and cyan ligands in sp^2^c‐COF are coordinated with Ir and 23.7% with Ru. Interestingly, metal absorption in the solution is quite efficient. In reality, 50% of the Ir species in solution are bound to the material, whereas 22% of the Ru precursors are bound to the framework. In addition, FT IR, ^13^C CP‐MAS NMR, and TGA for sp^2^c‐COF‐Ir‐ppy_2_ and sp^2^c‐COF‐Ru‐bpy_2_ showed no significant differences between the original and metalized COFs (see the Supporting Information). Note that the zeta potential of sp^2^c‐COF increased positively from −28.1 to −10.2 and +16.8 mV, after postsynthetic metalation of Ir and Ru (Figure S8, Supporting Information). The stability of sp^2^c‐COF‐Ir‐ppy_2_ and sp^2^c‐COF‐Ru‐bpy_2_ was also proven in neutral and weakly acidic physiological mediums, such as PBS (0.1 m, pH 7.4) and PBS (0.1 m, pH 6.0). As demonstrated in Figure S9 of the Supporting Information, both samples demonstrated superior dispersibility and stability in a given medium for over one week without forming any significant aggregations. Furthermore, the metal‐centered atoms in sp^2^c‐COF‐Ir‐ppy_2_ and sp^2^c‐COF‐Ru‐bpy_2_ were stable and negligible Ir or Ru‐release was observed for one‐week incubation in both neutral and weakly acidic conditions (Figure S9, Supporting Information).

The field emission scanning electron microscope (FESEM) images exhibited in Figure 2b–d manifest the similar layered morphology of three COFs, which are hundreds of nanometers in size. The transmission electron microscope (TEM) image reveals that sp^2^c‐COF, sp^2^c‐COF‐Ir‐ppy_2_, and sp^2^c‐COF‐Ru‐bpy_2_ possess a ribbon‐like layered structure, in good agreement with FESEM (Figure 2e–g). High‐resolution TEM (HRTEM) characterization of the same sample shows a cellular internal structure of COFs, with bright spots corresponding to the pores. The presence of evenly distributed Ir and Ru atoms in the framework was demonstrated using energy‐dispersive X‐ray spectroscopy in scanning transmission electron microscopy (TEM‐EDX) (Figure 2h–j). No Ir, Ru (or oxide) nanoparticles or clusters are detected in the spherical aberration‐corrected high‐angle annular dark‐field scanning TEM (SAC‐HAADF‐STEM) image. Single Ir and Ru atoms are identified in the SAC‐HAADF‐STEM image using Z‐contrast (Figure 2k–m).^[^ ^32^ ^]^

**Figure 3:**
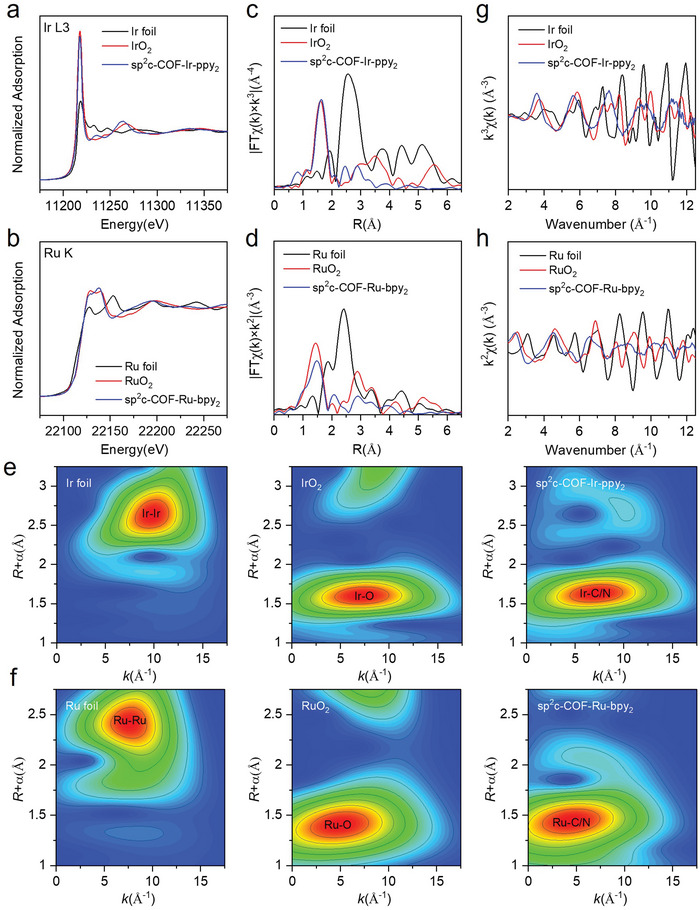
Structure of Ir and Ru SACs. a,b) Normalized Ir L_3_‐edge XANES of sp^2^c‐COF‐Ir‐ppy_2_ and sp^2^c‐COF‐Ru‐bpy_2_. c,d) FTEXAFS spectra of sp^2^c‐COF‐Ir‐ppy_2_ and sp^2^c‐COF‐Ru‐bpy_2_. e,f) WT of the sp^2^c‐COF‐Ir‐ppy_2_ and sp^2^c‐COF‐Ru‐bpy_2_. g,h) EXAFS fitting curves of sp^2^c‐COF‐Ir‐ppy_2_ and sp^2^c‐COF‐Ru‐bpy_2_ at *k* space.

Furthermore, X‐ray absorption fine structure spectroscopy (XAFS) tests were conducted to certify the dispersion and coordination environment of single Ir and Ru atoms. Compared with IrO_2_/Ir foil and RuO_2_/Ru‐foil as references, the X‐ray absorption near‐edge structure (XANES) spectra for Ir L_3_‐edge and Ru K‐edge of sp^2^c‐COF‐Ir‐ppy_2_ SACs and sp^2^c‐COF‐Ru‐bpy_2_ SACs are shown in **Figure** **3**a,b. According to the absorbed edge energy (Eb), the Ir and Ru atoms in SACs are in the oxidation states, and the pre‐edge peak of sp^2^c‐COF‐Ir‐ppy_2_ and sp^2^c‐COF‐Ru‐bpy_2_ is very close to that of IrO_2_ and RuO_2_.^[^ ^33^ ^]^ The fits are performed in the Fourier‐transformed space (*R* space) from the *k* ^2^‐weighted extended X‐ray absorption fine structure (EXAFS) data (Figure 3c,d). For IrO_2_ and Ir foil, the Ir SACs exhibit the main peak at 1.62 Å, and the peak of Ir–Ir cannot be detected at ≈2.6 Å in the Fourier transform EXAFS (FTEXAFS) curve, in accordance with the first coordination shell of Ir–C/N(O), indicating that Ir sites are atomically dispersed in sp^2^c‐COF‐Ir‐ppy_2_. In the FTEXAFS spectrum of Ru SACs, only one major peak is located at ≈1.5 Å, which is ascribed to the Ru–C/N(O) scattering pathway, and no Ru—Ru bond could be observed of ≈2.4 Å, indicating that the Ru atoms were atomically dispersed in sp^2^c‐COF‐Ru‐bpy_2_. The wavelet transformation (WT) diagram of Ir SACs in Figure 3e displays the WT maximum at 1.6 Å^−1^ between 1.5 and 1.7 Å^−1^ due to the comparison of Ir—C and Ir—N(O) bonds with that of IrO_2_ and Ir foil. Moreover, the maximum WT of Ru SACs ascribed to the Ru—C/N(O) bonding was 1.5 Å^−1^, and no corresponding maximum intensity of Ru—Ru was monitored compared to the WT diagrams of Ru foil and RuO_2_ (Figure 3f). Quantitative EXAFS analysis (Figure 3g,h) was performed for the calculation of structural parameters, and the fitting data are shown in Tables S2 and S3 of the Supporting Information. It is proved that the Ir atoms are fixed at the atomic level of the COF host, and the distance of Ir atom to the three N atoms (Ir–N) and three C atoms (Ir–C) is 2.02 Å, while the distance of Ru atom to the five N atoms (Ru–N) and one C atom (Ru–C) is 2.06 Å. The fitting graphs comport with the original data in *k*, *R*, and *q* space (Figures S10 and S11, Supporting Information), which testifies to the reliability of fitting results.

The presence of elements C, N, Ir, and Cl in sp^2^c‐COF‐Ir‐ppy_2_ and elements C, N, Ru, and Cl in sp^2^c‐COF‐Ru‐bpy_2_ is manifested in X‐ray photoelectron spectroscopy (XPS) measurement spectra (Figure S12a, Supporting Information). According to the Ir 4f XPS profiles (Figure S12b, Supporting Information), the sp^2^c‐COF‐Ir‐ppy_2_ Ir 4f signal band is deconvoluted into two peaks with Ir 4f_7/2_ at 62.0 eV and Ir 4f_5/2_ at 64.7 eV. In agreement with the above EXAFS results, due to the coordination of the C/N atom with the Ir ion, a slight variation of the two peaks toward lower binding energy is shown in sp^2^c‐COF‐Ir‐ppy_2_ compared to the dimer [Ir_2_(ppy)_4_Cl_2_], and the same phenomenon is examined in the previously reported metal‐N coordination catalysts.^[^ ^23b^ ^]^ Furthermore, high‐resolution XPS measurements of Ru 3d (Figure S12c, Supporting Information) indicate that the Ru 2p_3/2_ and Ru 2p_5/2_ binding energies are concentrated at 279.6 and 283.9 eV, respectively, with a small displacement of 0.94 and 0.49 eV, demonstrating the coordination of Ru with the thiazole and cyanogen ligands present in the framework.

### 2.2 ROS Generation

**Figure 4:**
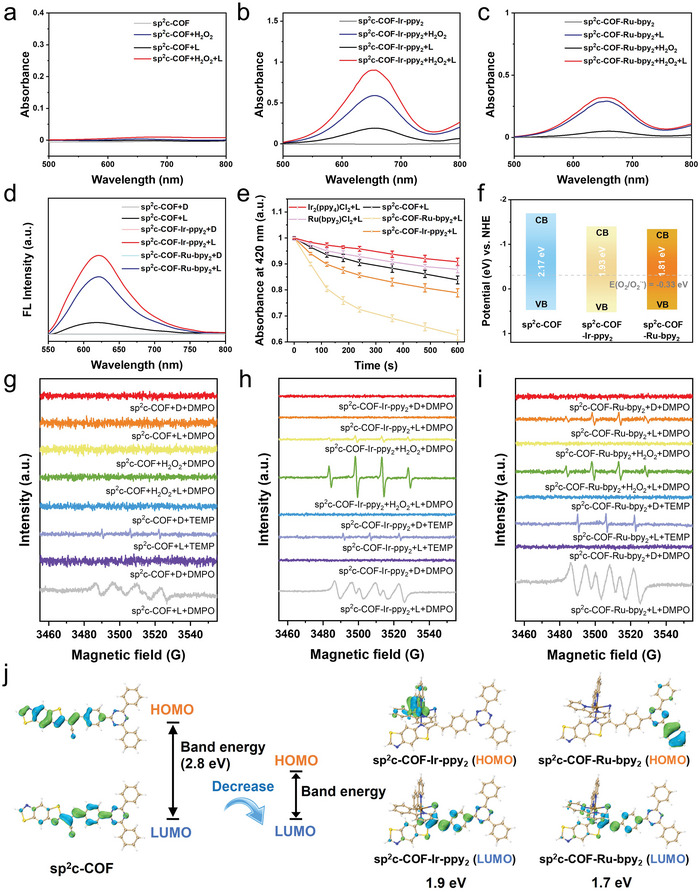
Photocatalytic ROS generation. a–c) UV–vis absorption spectra recording TMB oxidation over (a) sp^2^c‐COF, (b) sp^2^c‐COF‐Ir‐ppy_2_, and (c) sp^2^c‐COF‐Ru‐bpy_2_. d) Fluorescence emission spectra of DHE probe over sp^2^c‐COF, sp^2^c‐COF‐Ir‐ppy_2_, and sp^2^c‐COF‐Ru‐bpy_2_. e) DPBF degradation over sp^2^c‐COF, sp^2^c‐COF‐Ir‐ppy_2_, and sp^2^c‐COF‐Ru‐bpy_2_. Data are expressed as mean ± SD; *n* = 3. f) Band structure of sp^2^c‐COF, sp^2^c‐COF‐Ir‐ppy_2_, and sp^2^c‐COF‐Ru‐bpy_2_. g–i) ESR spectra of sp^2^c‐COF, sp^2^c‐COF‐Ir‐ppy_2_, and sp^2^c‐COF‐Ru‐bpy_2_ under different treatments. j) The molecular orbital diagrams of three COFs.

Next, the photoelectrochemical properties of sp^2^c‐COF, sp^2^c‐COF‐Ir‐ppy_2_, and sp^2^c‐COF‐Ru‐bpy_2_ were studied. According to the UV–vis diffuse reflectance spectra (UV–vis DRS), the three kinds of COFs present similar absorption curves and all have broad visible region absorption (Figure S13, Supporting Information). The Tauc plots were applied to calculate the optical bandgaps of sp^2^c‐COF, sp^2^c‐COF‐Ir‐ppy_2_, and sp^2^c‐COF‐Ru‐bpy_2_ to be 2.17, 1.93, and 1.81 eV, respectively (Figure S14, Supporting Information).^[^ ^34^ ^]^ From Mott–Schottky analyses, the valence band (VB) of COFs was then estimated to be 0.48, 0.53, and 0.47 eV versus standard hydrogen electrode (NHE) for sp^2^c‐COF, sp^2^c‐COF‐Ir‐ppy_2_, and sp^2^c‐COF‐Ru‐bpy_2_ (Figure S15, Supporting Information). The conductive band (CB) (vs NHE) was calculated accordingly, which were ≈−1.69 eV for sp^2^c‐COF, −1.40 eV for sp^2^c‐COF‐Ir‐ppy_2_, and −1.34 eV for sp^2^c‐COF‐Ru‐bpy_2_, respectively. The lower CB values of Ir SACs and Ru SACs suggest a stronger ability of them for electron transfer. For the electrochemical impedance spectroscopy assessment of electrical conductivity, sp^2^c‐COF‐Ru‐bpy_2_ showed the smallest semicircle radius (Figure S16, Supporting Information), indicating the lowest charge‐transfer resistance and the best‐photogenerated charges separation efficiency. It can be seen from the spectrum that the photoluminescence (PL) intensity of sp^2^c‐COF was weaker compared to that of sp^2^c‐COF‐Ir‐ppy_2_, and sp^2^c‐COF‐Ru‐bpy_2_, which demonstrated the presence of transition metal compounds inhibited the charge recombination (Figure S17, Supporting Information). And the time‐resolved PL emission attenuation spectrum exhibits that the sp^2^c‐COF‐Ir‐ppy_2_ and sp^2^c‐COF‐Ru‐bpy_2_ possess longer electronic lifetime, indicating the higher separation efficiency of photogenerated electrons and holes (Figure S18, Supporting Information). The transient photocurrent responses of as‐prepared samples with multiple switching cycles under intermittent visible‐light irradiation further clarify that the sp^2^c‐COF‐Ir‐ppy_2_ and sp^2^c‐COF‐Ru‐bpy_2_ possess higher electron transfer and charge separation efficiency than the pristine sp^2^c‐COFs (Figure S19, Supporting Information). Therefore, the integration of Ir, Ru ligands and sp^2^c‐COFs facilitates the separation of photogenerated electron–hole pairs and reinforces the light‐harvesting, logically leading to reinforced photocatalytic performance (**Figure** **4**f).

A series of 3,3′,5,5′‐tetramethylbenzidine (TMB) oxidation experiments were carried out to further elucidate the specific ROS generated by three COFs.^[^ ^35^ ^]^ The oxidation degree of TMB can be accessed by detecting the absorbance increases at ≈652 nm (Figure 4a–c). Before evaluating the performance of photocatalysis, we conducted the H_2_O_2_ catalytic decomposition assays to demonstrate the POD‐like activity of COFs. Only sp^2^c‐COF‐Ir‐ppy_2_ can convert the colorless compound TMB in the presence of H_2_O_2_ into oxidized TMB. The formation of hydroxyl radical (•OH) was confirmed by the characteristic absorbance peak at 652 nm. Overall, the results suggest that both sp^2^c‐COF‐Ir‐ppy_2_ and sp^2^c‐COF‐Ru‐bpy_2_ are capable of oxidizing TMB under 635 nm irradiation, and the former exhibits higher activity than sp^2^c‐COF‐Ru‐bpy_2_ due to extra POD‐like activity. Furthermore, dihydroethidium (DHE), which can solely react with superoxide anion (O_2_^•−^),^[^ ^36^ ^]^ was investigated with the three kinds of COFs (Figure 4d). The fluorescence emission spectra of DHE at 610 nm indicate that the production rate of O_2_^•−^ for sp^2^c‐COF‐Ir‐ppy_2_ is 1.4‐fold higher than that of sp^2^c‐COF‐Ru‐bpy_2_, highlighting the former's much greater ability in O_2_^•−^ production. In addition, 1,3‐diphenylisobenzofuran (DPBF) is a promising alternative for further verification of singlet oxygen (^1^O_2_) generation (Figure 4e). The reaction product of ^1^O_2_ and DPBF generates damped absorption at ≈420 nm. Consequently, for sp^2^c‐COF‐Ru‐bpy_2_, the absorption signal at 420 nm decreased significantly with the increase of irradiation time, which indicated the generation of ^1^O_2_ exceeded that of sp^2^c‐COF‐Ru‐bpy_2_ under irradiation with red light. By contrast, a slight degradation tendency can be observed at 420 nm for the dimeric metal complex, sp^2^c‐COF, and sp^2^c‐COF‐Ir‐ppy_2_ under similar conditions.

Electron spin resonance (ESR) spectroscopy is considered to be the most convincing evidence for ROS identification and has been adopted to further identify various ROS generated via sp^2^c‐COF, sp^2^c‐COF‐Ir‐ppy_2_, and sp^2^c‐COF‐Ru‐bpy_2_ (Figure 4g–i). The sp^2^c‐COF‐Ir‐ppy_2_ could produce •OH in the existence of H_2_O_2_, which is captured by 5,5‐dimethyl‐1‐pyrroline‐*N*‐oxide (DMPO) to form DMPO/•OH adducts, as shown in the 1:2:2:1 characteristic signal. Nevertheless, sp^2^c‐COF‐Ru‐bpy_2_ alone can also generate a noticeable ESR signal of DMPO/•OH. We conclude that this ROS species may be derived from the water molecules catalyzed by red light irradiation. Likewise, the DMPO is a radical scavenger for the identification of O_2_^•−^. The typical signal of DMPO‐OOH is observed from sp^2^c‐COF‐Ir‐ppy_2_, which further verified the O_2_^•−^ generation. In marked contrast to this is the fact that sp^2^c‐COF and sp^2^c‐COF‐Ru‐bpy_2_ give weaker signals. In addition, 2,2,6,6‐tetramethylpiperidine is a well‐known ^1^O_2_ probe used to scavenge ^1^O_2_ and produce the stable nitroxide radical 2,2,6,6‐tetramethylpiperidine‐N‐oxyl (TEMPO).^[^ ^37^ ^]^ The 1:1:1 triplet ESR signal of TEMPO for sp^2^c‐COF‐Ru‐bpy_2_ testifies to the generation of ^1^O_2_. Nevertheless, for sp^2^c‐COF and sp^2^c‐COF‐Ir‐ppy_2_, negligible weak signals are observed.

In order to understand the mechanism at the molecular level, HOMO or LUMO values for sp^2^c‐COF, sp^2^c‐COF‐Ir‐ppy_2_, and sp^2^c‐COF‐Ru‐bpy_2_ have been examined, respectively. The HOMO and LUMO distribution for sp^2^c‐COF, sp^2^c‐COF‐Ir‐ppy_2_, and sp^2^c‐COF‐Ru‐bpy_2_ are provided in Figure 4j. The bandgap between HOMO (−6.0 eV) and LUMO (−3.2 eV) orbitals for sp^2^c‐COF is determined to be 2.8 eV, which is 0.9 eV higher than that of sp^2^c‐COF‐Ir‐ppy_2_ (HOMO: −5.8 eV, LUMO: −3.9 eV). Further DFT calculations show that the HOMO/LUMO values are −4.0/−2.3 eV with the corresponding bandgaps of 1.7 eV for sp^2^c‐COF‐Ru‐bpy_2_. Compared to the sp^2^c‐COF with partial overlapping of the HOMO–LUMO electron cloud, the sp^2^c‐COF‐Ru‐bpy_2_ exhibits an entirely separated HOMO–LUMO electron cloud with extraordinarily strong charge‐transfer (CT) characteristics.^[^ ^27^ ^]^ The narrow band gap of sp^2^c‐COF‐Ir‐ppy_2_ and sp^2^c‐COF‐Ru‐bpy_2_ implies lower energy barriers for electron transport, hence better ferroptosis‐like effects for bacteria are expected.^[^ ^38^ ^]^

Based on previous reports,^[^ ^39^ ^]^ we reasoned the difference in the photocatalytic mechanism of both metalized COFs upon red light irradiation. It is hypothesized that the O_2_^•−^ generation is an electron transfer process. When sp^2^c‐COF‐Ir‐ppy_2_ is photoexcited, the excitons dissociate into charge carriers due to the energetic perturbations around the Ir centers. The electrons are then transported to O_2_ adsorbed at the Ir sites to provide O_2_^•−^, a process that has been widely investigated in semiconductor‐based photocatalytic systems. By contrast, the generation of ^1^O_2_ is more of an energy transfer process. After photoexcitation on sp^2^c‐COF‐Ru‐bpy_2_, the singlet excitons convert into the triplet state, which can activate the ground‐state O_2_ molecules to ^1^O_2_ via resonance energy transfer. The procedure does not require direct contact between the catalyst and oxygen, and it can be carried out through dipole–dipole interaction or charge exchange between donor and acceptor.^[^ ^40^ ^]^

### 2.3 In Vitro Bacterial Ferroptosis‐Like Analysis

**Figure 5:**
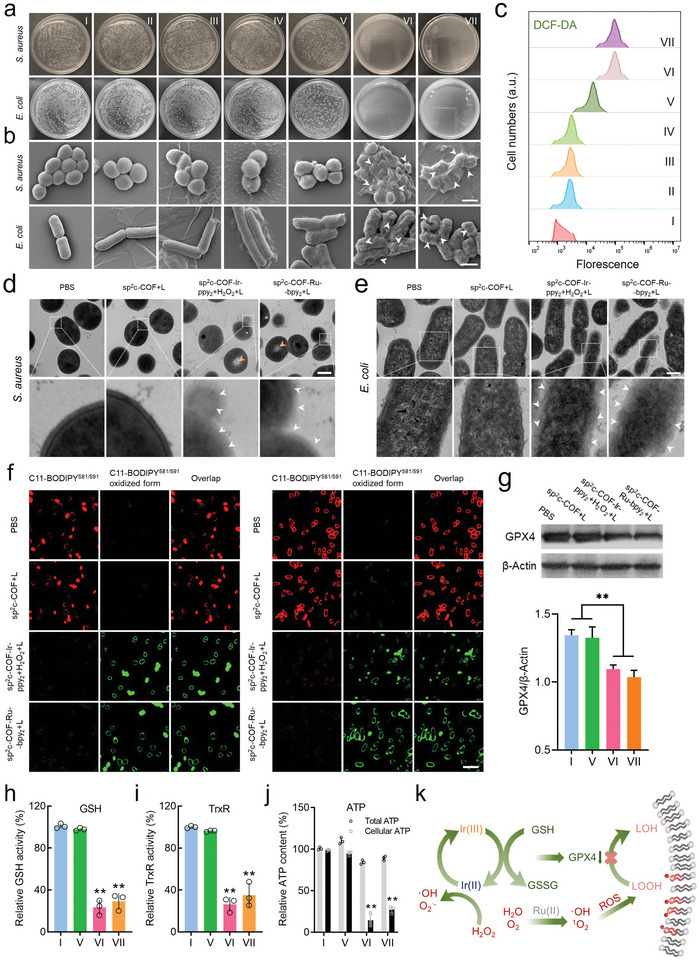
In vitro ferroptosis‐like effects. a) CFU counts, b) SEM images (scale bar = 1 µm), c) and intracellular ROS levels of *E. coli* and *S. aureus* cells treated with different formulas: (I) PBS, (II) sp^2^c‐COF, (III) sp^2^c‐COF‐Ir‐ppy_2_, (IV) sp^2^c‐COF‐Ru‐bpy_2_, (V) sp^2^c‐COF+Laser, (VI) sp^2^c‐COF‐Ir‐ppy_2_+H_2_O_2_+Laserm , (VII) sp^2^c‐COF‐Ru‐bpy_2_+Laser. d,e) TEM images of *E. coli* and *S. aureus* cells treated with PBS, sp^2^c‐COF‐Ir‐ppy_2_+H_2_O_2_, and sp^2^c‐COF‐Ru‐bpy_2_ under red light irradiation. Scale bar = 500 nm. f) CLSM images of C11‐BODIPY^581/591^ dye‐stained *E. coli* and *S. aureus* cells after being incubated with PBS, sp^2^c‐COF‐Ir‐ppy_2_+H_2_O_2_, and sp^2^c‐COF‐Ru‐bpy_2_ under red light irradiation. Scale bar = 5 µm. g) Western blot analysis of GPX4 protein in *E. coli* cells with varied conditions. Data are expressed as mean ± SD; *n* = 3. h) Intracellular GSH, i) TrxR, j) and ATP levels of *E. coli* cells in I, V, VI, VII groups. Data are expressed as mean ± SD; *n* = 3. k) Schematic diagram of LPO used in cellular membrane damage. Statistical significance was determined using a one‐way ANOVA test with a Bonferroni's comparison test, giving *P* values, * denotes *P* < 0.05, ** denotes *P* < 0.01.

Next, we tested the antibacterial performance of COF‐based SACs in vitro by counting the colony‐forming unit (CFU) on an agar plate. Notably, on account of the optimal catalytic conditions, we established seven different groups to investigate the antimicrobial performance against bacteria, including (I) PBS, (II) sp^2^c‐COF, (III) sp^2^c‐COF‐Ir‐ppy_2_, (IV) sp^2^c‐COF‐Ru‐bpy_2_, (V) sp^2^c‐COF+Laser, (VI) sp^2^c‐COF‐Ir‐ppy_2_+H_2_O_2_+Laser, and (VII) sp^2^c‐COF‐Ru‐bpy_2_+Laser groups. High H_2_O_2_ levels can cause a sharp decline in the relative viability of *Escherichia coli* and *Staphylococcus aureus* and may lead to wound ulceration. Therefore, a lower concentration of H_2_O_2_ (1 × 10^−3^ m) was selected for group VI to conduct subsequent experiments (Figure S20, Supporting Information). As shown in **Figure** **5**a, there was no difference in CFU between the I, II, III, and IV groups, suggesting that the unirradiated COFs exhibited no antimicrobial activity. Furthermore, the bacterial viability decreased only slightly after incubation with sp^2^c‐COF exposed to a 635 nm laser, as the pure photocatalytic carrier sp^2^c‐COF exhibited limited antibacterial activity. As expected, a significant inhibition occurred when sp^2^c‐COF‐Ir‐ppy_2_ (containing 1 mm H_2_O_2_) and sp^2^c‐COF‐Ru‐bpy_2_ were irradiated with red light (0.4 W cm^−2^). Figure S21 of the Supporting Information shows the performance for different concentrations of COFs in inhibiting *S. aureus* and *E. coli*. The minimum inhibitory concentrations (MICs) of COFs on various types of bacteria are revealed in Table S4 of the Supporting Information. Compared with sp^2^c‐COF, photoactivated sp^2^c‐COF‐Ir‐ppy_2_ and sp^2^c‐COF‐Ru‐bpy_2_ show significantly lower MIC. More importantly, we found that Ir and Ru SACs (256 µg mL^−1^) could also kill 98.5% and 94.9% MRSA pathogens, respectively (Figure S22, Supporting Information).

To further investigate the antimicrobial properties of the three kinds of COF, SEM was utilized to examine the morphological transformation of bacteria treated with different samples. In the absence of visible light, treatment with various COFs resulted in no significant morphological changes in *S. aureus* and *E. coli*, while the sp^2^c‐COF‐Ir‐ppy_2_‐treated and sp^2^c‐COF‐Ru‐bpy_2_‐treated bacteria irradiated with light revealed collapsed morphologies and the loss of intracellular content (Figure 5b). We applied 3,3′‐dipropylthiadicarbocyanine iodide (DISC_3_(5)) to detect the variations in membrane potentials after different treatments by flow cytometry (Figure S23, Supporting Information). Slight increases in fluorescence were observed for the sp^2^c‐COF group upon light irradiation (Group V). By contrast, after the Ir and Ru SACs treatments (Group VI and VII) with light irradiation, a marked increase in the fluorescence of bacterial cells was observed, revealing depolarization and damage of the bacterial membranes.^[^ ^41^ ^]^ In addition, the ingestion of propyl iodide (PI) also contributes to the increase of membrane permeability (Figure S24, Supporting Information). Furthermore, we quantitatively analyzed the contents of protein and nucleic acid in the supernatants of various treated *S. aureus* cells, and the results showed that the bacteria treated by Ir and Ru SACs possessed the highest degree of leaked proteins and nucleic acids under light irradiation, which was consistent with the loss of intracellular content (Figure S25, Supporting Information).

Next, we examined photoirradiation‐initiated ROS production in bacterial cells. Considering that two parallel and distinct antioxidant systems exist in Gram‐negative bacteria,^[^ ^42^ ^]^ the GSH and thioredoxin (Trx) systems are more capable of controlling the cellular redox environment than the solitary Trx system in Gram‐positive bacteria, we chose *E. coli* as the model strain for ROS study. *E. coli* cells were treated with sp^2^c‐COF, sp^2^c‐COF‐Ir‐ppy_2_, sp^2^c‐COF‐Ru‐bpy_2_, and the intracellular ROS level was determined by DCFH‐DA assay. As shown by flow cytometry (Figure 5c), treatment with three COFs caused almost no alternations in intracellular ROS levels in the absence of irradiation. Compared with the cells in Groups I, II, III, and IV, both the Ir SACs+H_2_O_2_ and Ru SACs treated cells exhibited significantly increased ROS levels after irradiation. The ROS production capacity of SACs in living bacterial cells was further investigated. Aminophenyl fluorescein (APF), DHE, and singlet oxygen sensor green (SOSG) were applied as selective fluorescence probes for measuring the amounts of the •OH, O_2_^•−^, and ^1^O_2_, respectively.^[^ ^43^ ^]^ Results showed that (Figure S26, Supporting Information) under photoirradiation, the fluorescence emission of •OH‐related APF in sp^2^c‐COF‐Ir‐ppy_2_+H_2_O_2_ and sp^2^c‐COF‐Ru‐bpy_2_ group was remarkably higher than that in the control and sp^2^c‐COF group, which was similar to the results of TMB oxidization investigation. The O_2_^•−^ associated DHE fluorescence indicated almost no change in bacteria with a 635 nm laser irradiation in the sp^2^c‐COF group (Figure S27, Supporting Information). Under laser irradiation, the DHE fluorescence was elevated in Group VI and VII. Additionally, as shown in Figure S28 of the Supporting Information, no apparent SOSG fluorescence signal associated with ^1^O_2_ was observed in bacteria after the treatment of sp^2^c‐COF. By contrast, cells treated with Ir and Ru SACs produced large amounts of ^1^O_2_ under red light irradiation. These overproduced ROS could lead to oxidative damage of cytomembranes, contributing to the emergence of LPO and increased sensitivity to ferroptosis.

Thereafter, the ferroptosis‐like induced by excess ROS was investigated. From both SACs‐induced ferroptosis‐like mechanisms for bacteria illustrated in Figure 5k, LPO accumulation and GPX4 downregulation are believed to be the crucial factors in ferroptosis‐like. As shown in Figure 5d,e, cytomembrane with cytoplasmic decomposition (marked by yellow arrows) was observed in the sp^2^c‐COF‐Ir‐ppy_2_ and sp^2^c‐COF‐Ru‐bpy_2_ group exposed to 635 nm irradiation compared to the pristine bacteria, indicating the accumulation of LPO. Generally speaking, the typical LPO process consists of three steps: 1) the initiation step where emerging ROS attack unsaturated lipids and generate lipid free radicals; 2) the propagation period in which the LPO radicals are produced in the presence of oxygen; and 3) the termination step that the lipid hydroperoxides (LOOH) decompose into smaller molecular by‐products, which contain malondialdehyde (MDA).^[^ ^5^, ^44^ ^]^ As an LPO sensor, C11‐BODIPY^581/591^ can be inserted into lipid membranes and oxidized by LPO to monitor the intracellular LPO levels.^[^ ^13b^ ^]^ As shown in Figure 5f, the *E. coli* and *S. aureus* cells treated with sp^2^c‐COF‐Ir‐ppy_2_+H_2_O_2_ and sp^2^c‐COF‐Ru‐bpy_2_ under red light irradiation exhibited significantly stronger C11‐BODIPY^581/591^ oxidation fluorescence than the other groups, indicating the elevated intracellular ROS induced LPO accumulation. During this process, the key indicators of MDA in LPO are also evidently increased (Figure S29, Supporting Information). Moreover, the degree of DNA degradation also increased with the enhancement of LPO (Figure S30, Supporting Information). On the other hand, GSH plays an auxiliary role in the GPX4‐catalyzed lipid repair systems, and its depletion can inactivate GPX4, thereby promoting the ferroptosis‐like in Gram‐negative cells.^[^ ^7^ ^]^ As indicated by the absorption changes (412 nm) of DTNB (5,5′‐dithiobis‐(2‐nitrobenzoic acid)),^[^ ^45^ ^]^ the Ir and Ru SACs could effectively consume GSH as the incubation time increased, particularly in the Ir SACs group (Figure S31, Supporting Information). ^1^H NMR spectra confirmed the gradual oxidation of GSH in the presence of Ir and Ru SACs (Figure S32, Supporting Information). The complete conversion rate was observed 1 h later in the Ir SACs group, consistent with commercially available oxidized glutathione. We further investigated the effects of metalized COFs on intracellular GSH levels. As illustrated in Figure 5h, the other two SACs groups except the sp^2^c‐COF group showed significantly reduced GSH content in *E. coli* cells. In addition, as shown in Figure 5g, western blots demonstrate that GPX4 protein expression was downregulated in the sp^2^c‐COF‐Ir‐ppy_2_ and sp^2^c‐COF‐Ru‐bpy_2_ groups. The GPX4 inactivation is especially advantageous to block the LPO elimination, suggesting that Ir and Ru SACs significantly suppress the defense and repair functions of lipid antioxidants, thereby promoting ferroptosis‐like in Gram‐negative bacterial cells. In contrast to Gram‐negative germs, thioredoxin reductase (TrxR) depletion in Gram‐positive bacteria that relies exclusively on the Trx–TrxR pathway for antioxidant defense could accelerate the accumulation of LPO and subsequently trigger bacterial ferroptosis‐like. As expected, the results in Figure 5i illustrated that Ir and Ru SACs were able to potently inhibit TrxR activity in *S. aureus* cells. However, the control and sp^2^c‐COF alone showed no effect on TrxR activity. Adenosine triphosphate (ATP)‐bound respiration represents the ATP synthesizing ability of ATP synthase to meet the energy requirements of cells.^[^ ^46^ ^]^ The ATP synthesis ability of ATP synthase was significantly reduced when sp^2^c‐COF‐Ir‐ppy_2_+H_2_O_2_ or sp^2^c‐COF‐Ru‐bpy_2_ was used alone under visible light irradiation (Figure 5j). The experiment confirmed that the synthesis of ATP in bacteria was damaged under Ir and Ru SACs stress, affecting the metabolic ability and normal respiration of bacteria.^[^ ^11^, ^47^ ^]^ From the above results, we can conclude that the COF‐based SACs could effectively induce ROS‐mediated bacterial ferroptosis‐like.

**Figure 6:**
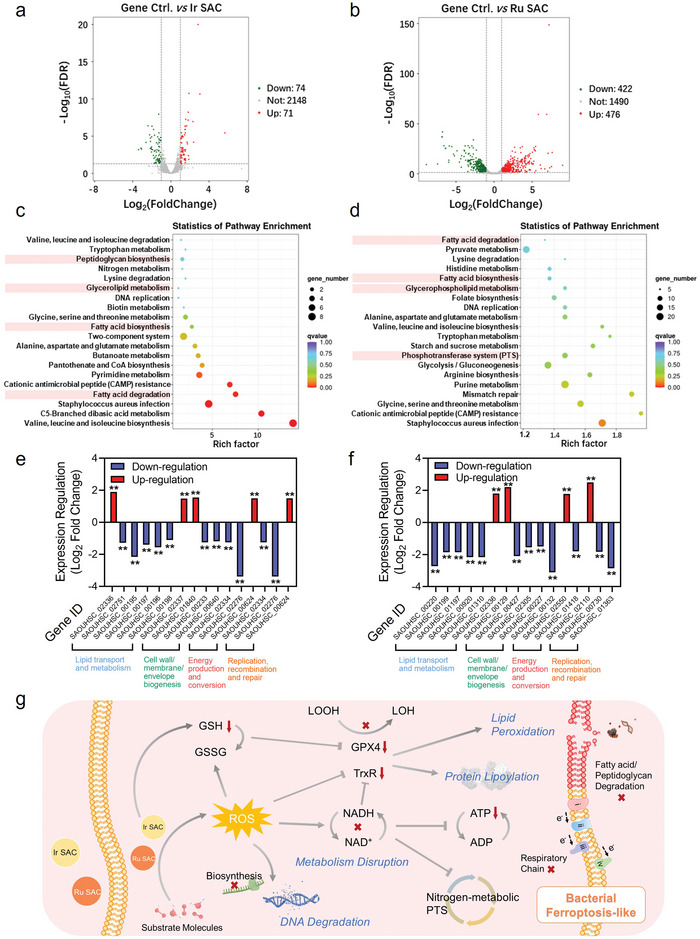
Changes in the MRSA transcriptome. Volcano map for the distribution of DEGs in a) Ir SAC and b) Ru SAC groups compared with Ctrl. c,d) Upregulated and downregulated gene ontology enrichment analysis in (a) Ir SAC and (b) Ru SAC compared with Ctrl. e,f) Expression changes of the lipid transport and metabolism, cell wall/membrane/envelope biogenesis, energy production and conversion, and replication, recombination and repair of Ctrl and Ir SAC, Ru SAC. Data are expressed as log_2_ fold change; *n* = 3. g) Schematic diagram of the Ir and Ru SACs‐mediated bacterial ferroptosis‐like mechanism based on transcriptomic analysis. Statistical significance was determined using a one‐way ANOVA test with a Bonferroni's comparison test, giving *P* values, * denotes *P* < 0.05, ** denotes *P* < 0.01.

The effect of COF‐based SACs on MRSA gene expression was studied by transcriptomic analysis.^[^ ^48^ ^]^ Volcano plots revealed remarkable differences in MRSA gene expression between the control group (Ctrl) and the Ir SACs group. A total of 2148 genes were expressed in the two groups, while exclusively 74 genes were downregulated and 71 genes were upregulated in the Ir‐SACs‐treated group (**Figure** **6**a). Likewise, compared to the Ctrl group, Volcano plots exhibited 112 significantly differentially expressed genes (DEGs) in the Ru SACs group, 422 of which were downregulated and 476 were upregulated (Figure 6b). To further elucidate the influence of COF‐based SACs on MRSA, Kyoto Encyclopedia of Genes and Genomes (KEGG) pathway enrichment analysis and Gene Ontology (GO) enrichment analysis was performed. The enrichment analysis of KEGG pathway exhibited that the effects of Ir SACs on MRSA were highly concentrated in fatty acid biosynthesis and metabolism, glycerolipid metabolism, and peptidoglycan biosynthesis, while Ru SACs were mainly interfering with the biosynthesis and degradation of fatty acid, metabolism of glycerophospholipid, and phosphotransferase system (Figure 6c,d). The GO enrichment analysis of Ir and Ru SACs also indicated that the significant genes were basically enriched in the metabolic process, cellular process, membrane and membrane part, catalytic activity, transporter activity, and antioxidant activity (Figure S33a,b, Supporting Information).

Next, we concluded the DEGs information of Ir and Ru SACs compared to the Ctrl group to study the MRSA death (Figure 6e,f). 1) The genes comprising SAOUHSC_02236, SAOUHSC_02751, SAOUHSC_01310, SAOUHSC_00220, SAOUHSC_00195, SAOUHSC_00197, SAOUHSC_00196, etc., which encoded key enzymes related with lipid transport and metabolism, were appreciably downregulated and upregulated. 2) Several genes (e.g., SAOUHSC_02337, SAOUHSC_02305, SAOUHSC_01840, SAOUHSC_00427, SAOUHSC_00233, etc.) closely related to the cell wall/membrane/envelope biogenesis were considerably changed. These differences indicated that the lipid bilayer was notably diffused and disturbed, the peptidoglycan was hydrolyzed, and the cell membrane was damaged, supporting the view of the LPO process in plasmalemma. 3) The genes (SAOUHSC_02334, SAOUHSC_00132, SAOUHSC_02276, SAOUHSC_02550, etc.) were related to the energy production and conversion during MRSAs ferroptosis‐like, meaning that the ATP generation in MRSA was significantly suppressed. It suggested that the energy metabolism of MRSA might change from aerobic to anaerobic respiration. 4) The expression of genes related to the replication, recombination and repair in the Ir SAC group (SAOUHSC_02334, SAOUHSC_02276, SAOUHSC_00624, etc.) and in the Ru SAC group (SAOUHSC_02110, SAOUHSC_00730, SAOUHSC_01363, etc.) was markedly regulated, meaning that bacterial genetic systems were further disrupted by the significant oxidative stress. 5) Beyond these physiological activities, the expressions of genes associated with oxidoreductase activity (SAOUHSC_00382), protein lipoylation (SAOUHSC_01159), and response to oxidative stress (SAOUHSC_00831) were all upregulated, which is consistent with our previous observations that Ir and Ru SACs will enhance ferroptosis‐like derived bacterial death through the accumulation of LOOH. The abovementioned trends evidently demonstrated that both COF‐based SACs could damage the fatty acid or peptidoglycan, disrupt the antioxidant system, impair nitrogen and respiratory metabolisms, and render the biosynthesis of DNA dysfunctional, which ultimately induced pronounced ferroptosis‐like damage in vitro for efficient bacteria eradication (Figure 6g).

Bacterial biofilms possess inherent resistance to antibiotics and the immune system, which are difficult to eradicate, and can lead to a variety of chronic bacterial infections. Therefore, we evaluated the inhibition effect of these ferroptosis‐like inducers on MRSA biofilm formation. 3D reconstructions of green fluorescence‐labeled live MRSA biofilms dyed with SYTO 9 were conducted to determine the transformation of biofilm structure after different treatments (Figure S34, Supporting Information). For the PBS and sp^2^c‐COF+Laser groups, bright green fluorescence signal was observed, suggesting intact bacterial biofilms. Scattered biomass and cell clusters with weak green fluorescence were observed in the sp^2^c‐COF‐Ir‐ppy_2_+H_2_O_2_+Laser and sp^2^c‐COF‐Ir‐ppy_2_+Laser groups. These results clearly depicted that Ir and Ru SACs‐mediated bacterial ferroptosis‐like treatment can efficiently decompose bacterial biofilms, validating their efficiency in biofilm elimination.

### 2.4 In Vivo Bacterial Ferroptosis‐Like and Biocompatibility Assessments

**Figure 7:**
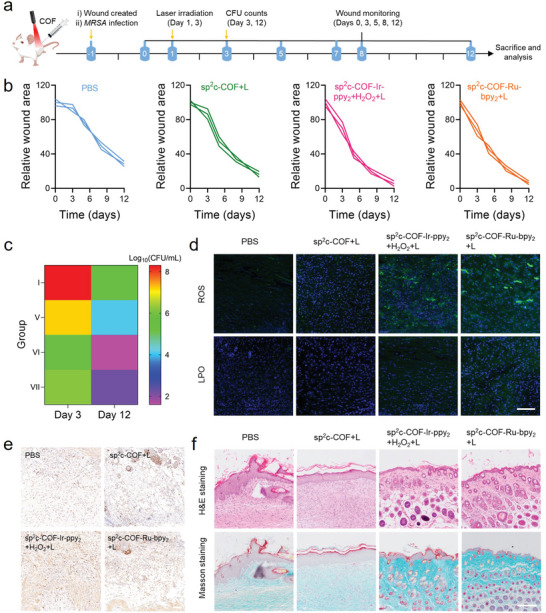
In vivo ferroptosis‐like effects. a) Schematic diagram of COF‐based SACs‐induced bacterial ferroptosis‐like assessment. b) Quantitative analysis of the residual wounded areas that received different treatments. c) Bacterial burden around the infected wounds measured with the dilution coating plate method on days 3 and 12. d) Wound tissue slices containing DAPI, DCFH‐DA, and C11‐BODIPY^581/591^. Scale bar = 100 µm. e) Histochemical staining images of Ki67‐labeled positive cells after treatment with various COFs in wound sites. Scale bar = 100 µm. f) H&E and Masson's staining of the infected skin slices on day 12. Scale bar = 200 µm.

Motivated by the expected in vitro ferroptosis‐like therapeutic effect, the in vivo ferroptosis‐like assay was performed in the mice model. A full‐thickness skin wound about 15 mm in diameter was produced in the shaved skin of mice and immediately inoculated with MRSA bacteria. 24 h later, these infected mice were separated into seven groups, which received the treatment of PBS, sp^2^c‐COF with or without irradiation, sp^2^c‐COF‐Ir‐ppy_2_ with or without H_2_O_2_ (1 mm) and light irradiation, and sp^2^c‐COF‐Ru‐bpy_2_ with or without light irradiation, respectively (**Figure** **7**a). The light‐exposed sp^2^c‐COF‐Ir‐ppy_2_+H_2_O_2_ and sp^2^c‐COF‐Ru‐bpy_2_ groups showed the highest rate of wound closure within 12 days, exhibiting a much better healing promotion than the other groups (Figure 7b; Figure S35, Supporting Information). As shown in Figure 7c, the spread plate test was utilized to calculate the CFU in the wound tissues of the 3rd and 12th days. Compared with the control and sp^2^c‐COF groups, the Ir and Ru SACs induced ferroptosis‐like therapy suppressed the growth of MRSA and achieved a higher antibacterial effect, eliminating most bacteria (3–4 log_10_ CFU mL^−1^) in vivo. These results demonstrated that COF‐based SAC could achieve different antimicrobial therapy modes in vivo. The ROS proliferation and LPO accumulation of bacterial ferroptosis‐like stimulated by SACs in an infected microenvironment were further evaluated. By DCFH‐DA staining, ROS in tissue for both sp^2^c‐COF‐Ir‐ppy_2_+H_2_O_2_+Laser and sp^2^c‐COF‐Ru‐bpy_2_+Laser groups present green fluorescence, which differs significantly from that in the control group (Figure 7d). It was demonstrated that Ir and Ru‐SACs treatments produced ROS in the infected microenvironment. As expected, the oxidative fluorescence of C11‐BODIPY^581/591^ dye labeling and green staining show notably enhanced intensity in sp^2^c‐COF‐Ir‐ppy_2_+H_2_O_2_+Laser and sp^2^c‐COF‐Ru‐bpy_2_+Laser groups compared with the control group, further illustrating the in vivo potency of Ir and Ru SACs in bacterial ferroptosis‐like (Figure 7d).

To investigate whether excess ROS long‐term harms normal cells at the infected sites, Ki67 was used to stain the proliferating cells for assessing mitotic activity. Immunohistochemical staining revealed that sp^2^c‐COF‐Ir‐ppy_2_ and sp^2^c‐COF‐Ru‐bpy_2_ were more competent than sp^2^c‐COF in advancing the expression of Ki67‐positive cells, indicating that the Ir and Ru SACs groups showed the highest cell proliferation activity (Figure 7e). The growth of granulation tissue was observed, representing the degree of wound healing. As show by hematoxylin and eosin (H&E) staining, the granulation of the control group was still sparse, while that of the group that received bacterial ferroptosis‐like was dense and favorable (Figure 7f). Masson's staining was used to assess the aggregation of collagen fibers in the regenerated skin tissue (Figure 7f). The sp^2^c‐COF‐Ir‐ppy_2_ and sp^2^c‐COF‐Ru‐bpy_2_ groups show deep collagen staining and dense structure, indicating a considerable accumulation of collagen fibers around the wound.^[^ ^41^ ^]^ However, collagen deposition was still limited in the PBS and sp^2^c‐COF groups. In consequence, both SACs could promote granulation formation and enhance collagen deposition and remodeling to promote wound reconstruction.

We next sought to evaluate the biocompatibility of Ir and Ru SACs with a sequence of systematic toxicity experiments. No apparent hemolysis was examined for sp^2^c‐COF, sp^2^c‐COF‐Ir‐ppy_2_, and sp^2^c‐COF‐Ru‐bpy_2_ at all concentrations tested (0–256 mg mL^−1^). In addition, the nanoparticles at concentrations up to 256 mg mL^−1^ still showed ignorable toxicity toward normal mammalian cells such as L929 fibroblast cells and human umbilical vein endothelial cells (Figure S36, Supporting Information). The sample was administered by intraperitoneal injection of PBS suspension. Three days after the administration, the mice were sacrificed according to the regulations of the ethical committee. We found no material accumulation in the area of the peritoneal injection site. We also examined the organ structure using H&E staining to detect any type of tissue damage (Figure S37, Supporting Information). There was no evidence of local accumulation of inflammatory cells in any of the tissue slices. Whole blood analysis and biochemical blood indicators of mice in each group exhibited that compared to the control group, all samples did not affect the levels of white blood cells, red blood cells, platelets, and hemoglobin (Figure S38, Supporting Information). Liver enzymes and renal function indicators were also analyzed after a treatment period. The results showed that aspartate transferase (AST), alanine transferase (ALT), creatinine (CRE), and blood urea nitrogen (BUN) were all within the normal range (Figure S38, Supporting Information).^[^ ^49^ ^]^ Therefore, Ir and Ru SACs can effectively kill bacterial pathogens and scarcely exhibit any hemolysis and cytotoxicity, holding excellent application prospects in anti‐infection.

### 2.5 In Vivo Abscess Healing Evaluation

**Figure 8:**
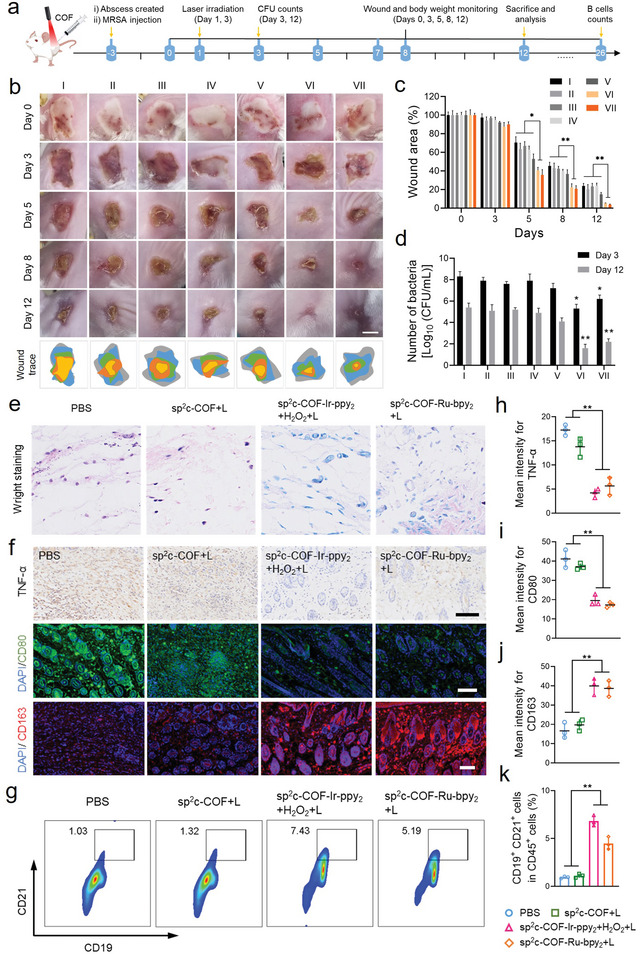
In vivo abscess healing assessments. a) Schematic depicting the treatment regime in MRSA‐infected abscess model. b) Representative images of each group infected with abscesses at given time points (scale bar = 10 mm). c) Statistical graph of residual lesion area in the process of 12 days of treatment. Data are expressed as mean ± SD; *n* = 3. d) CFU of MRSA from infected skin tissues on days 3 and 12. Data are expressed as mean ± SD; *n* = 3. e) Wright's staining of lesion sites on day 12. f) Immunohistochemistry and immunofluorescence images of TNF‐*α*, CD80, CD163 staining of lesion sites after different treatments on day 12. Scale bar = 100 µm. g) Flow cytometry assay for B cells (CD19^+^/CD21^+^/CD45^+^). h) Quantification of the average intensity in TNF‐*α* positive regions. i–j) Quantification of the mean fluorescence intensity in CD80 and CD163 positive regions. k) Calculated percentages of B cells from (g). Data are expressed as mean ± SD; *n* = 3. Statistical significance was determined using a one‐way ANOVA test with a Bonferroni's comparison test, giving *P* values, * denotes *P* < 0.05, ** denotes *P* < 0.01.

Current conventional antibiotics are unavailable for good efficacy against cutaneous abscess infections, thereby developing innovative therapeutic agents to treat abscesses effectively is highly desirable.^[^ ^50^ ^]^ We injected mice subcutaneously with MRSA microbes, causing skin abscesses. Three days after infection, cutaneous abscesses developed with apparent dermonecrotic and white lesions (filled with fluid/pus). The infected mice were randomly separated into seven groups that received different treatments including PBS, sp^2^c‐COF, sp^2^c‐COF‐Ir‐ppy_2_ (containing 1 mm H_2_O_2_), sp^2^c‐COF‐Ru‐bpy_2_ without and with 635 nm light irradiation, respectively. At the scheduled time, the abscesses were imaged, total bacterial counts in the skin lesion were determined, and a histological examination was also performed to evaluate the lesion (**Figure** **8**a). First, the changes of the lesions in each group were captured and the final healing areas were quantified (Figure 8b). By day 12, abscesses in the sp^2^c‐COF‐Ir‐ppy_2_+H_2_O_2_+Laser and sp^2^c‐COF‐Ru‐bpy_2_+Laser groups were almost closed, whereas the abscess in the other two groups remained clearly unhealed (Figure 8c). Quantitative measurements of the abscess healing process were also carried out; the sp^2^c‐COF‐Ir‐ppy_2_+H_2_O_2_+Laser group exhibited the fastest wound healing rate of 97.3%, whereas the sp^2^c‐COF‐Ru‐bpy_2_+Laser group showed the second fastest cure rate of 94.8%. The healing rate of both groups was remarkably higher than that of the other two groups. Quantitative CFU analysis of various abscess tissues after treatment exhibited the least bacterial burden of Ir and Ru SACs‐treated group under 635 nm light irradiation (Figure 8d). Further, within one week after subcutaneous injection of MRSA, the mean body weight of the surviving mice in the photoirradiation groups with sp^2^c‐COF‐Ir‐ppy_2_+H_2_O_2_ and sp^2^c‐COF‐Ru‐bpy_2_ returned to the normal range (Figure S39, Supporting Information), indicating that the mice were effectively protected from rechallenged bacteria even under lethal in vivo injection.

To further explore the progress of abscess healing, histomorphological examination of the lesion tissue was performed by H&E and Masson's staining after 12 days. The wounded skin in the sp^2^c‐COF‐Ir‐ppy_2_ and sp^2^c‐COF‐Ru‐bpy_2_ groups exhibited better reepithelization and healthier dermal components, including hair follicles and sebaceous glands, compared with the PBS and sp^2^c‐COF groups (Figures S40 and S41a, Supporting Information). Collagen deposition in the healing tissue was evaluated by Masson's staining, in which collagen was labeled in blue and keratin or muscle fiber was labeled in red. As observed from the Masson's staining (Figure S40, Supporting Information), collagen in damaged skin tissue was significantly reduced, resulting in poor wound healing and impaired tissue remodeling. The sp^2^c‐COF‐Ir‐ppy_2_ and sp^2^c‐COF‐Ru‐bpy_2_ groups showed more collagen deposition, and the collagen fibers in the skin tissues were denser, thicker, and more neatly arranged. Therefore, Ir and Ru SACs combined with visible light could highly promote the tissue regeneration and healing process of the MRSA‐infected abscess.

Local inflammatory reaction affects the whole abscess healing process. Neutrophils and eosinophils described as granulocytes are the most numerous types of white blood cells that rapidly reach the sites of injury or infection and are identified by their red‐staining granules.^[^ ^51^ ^]^ Images from the Wright‐stained sample contain many red‐staining granules in the wounded tissues in the PBS and sp^2^c‐COF groups, indicating severe inflammatory cell infiltration (Figure 8e; Figure S41b, Supporting Information). In comparison, the quantity of red‐staining granules in the sp^2^c‐COF‐Ir‐ppy_2_ and sp^2^c‐COF‐Ru‐bpy_2_ groups is lower than that in the control group, proving relatively minor inflammation. Immunohistochemical staining indicated that sp^2^c‐COF‐Ir‐ppy_2_ and sp^2^c‐COF‐Ru‐bpy_2_ were more capable of knocking down the level of proinflammatory cytokines in lesions, including TNF‐*α*, IL‐1*β*, and IL‐6 compared with individual treatment (Figure 8f,h; Figure S42, Supporting Information). We attempted to explain this behavior by immunofluorescent double staining of CD80 and CD160, as it was previously reported to be related to M2 macrophage polarization.^[^ ^52^ ^]^ In this regard, visual examination of the stained images and quantitative results revealed that the Ir and Ru SACs possess the minimum number of CD86^+^ proinflammatory M1 macrophages and the maximum number of CD206^+^ anti‐inflammatory M2 macrophages (Figure 8f,i,j). Our result confirmed that our final formulation plays a functional role in suppressing inflammatory responses and immunoregulation in abscess healing through this hypothetical pathway, achieving the initiation of the proliferative phase of the healing cascade.

Angiogenesis is an indispensable step in the remodeling stage of abscess healing. M2d‐type macrophages could secrete vascular endothelial growth factor (VEGF) and increase the proliferation of vascular endothelial cells to promote angiogenesis. Next, we studied the expression of VEGF in vivo in established abscesses by immunohistochemical staining, while staining newly formed vessels with a cluster of differentiation 31 (CD31). VEGF expression was lower in the lesion tissues of the control and sp^2^c‐COF groups, while Ir and Ru SACs treatment remarkably rescued the associated protein expression in the damaged lesion (Figure S43, Supporting Information). A similar trend was observed, with significantly positive CD31 staining in the Ir and Ru SACs groups compared to the other two control specimens. This result indicated that both types of SACs could promote angiogenesis in MRSA‐infected abscesses.

**Scheme 2:**
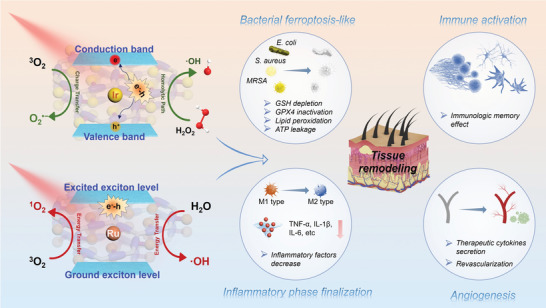
A schematic illustration for the therapeutic mechanism of Ir and Ru SACs through the pathways of bacterial ferroptosis‐like death, advanced termination of the inflammatory phase, proangiogenesis, and immune activation.

On day 26, the mice were divided into four groups, each with two subgroups. The first subgroup was euthanized to assess B cell levels (CD19^+^/CD21^+^/CD45^+^) by flow cytometry assay, while the second subgroup was re‐exposed to the same doses of the MRSA bacteria as the original administration (1.0 × 10^7^ CFU mL^−1^, *i.h*.) without any further treatment over the next few days. To analyze the immunological memory level induced by the sp^2^c‐COF‐Ir‐ppy_2_ and sp^2^c‐COF‐Ru‐bpy_2_ treatments, peripheral blood B cells were evaluated via flow cytometry assay, which indicates that the expression in the group previously treated with sp^2^c‐COF‐Ir‐ppy_2_+H_2_O_2_ and sp^2^c‐COF‐Ru‐bpy_2_ was notably increased under red light radiation, compared with the other groups (Figure 8g,k). It was thus confirmed that the immunogenicity enhancement caused by this bacterial ferroptosis‐like inducer Ir and Ru SACs could trigger a potent and long‐term immune memory effect to minimize the risk of infection recurrence. Therefore, COFs‐based SACs accelerated drug‐resistance bacteria‐infected abscess healing by inducing bacterial ferroptosis‐like death, advancing termination of the inflammatory phase, promoting angiogenesis, and activating immune systems (**Scheme** **2**).

## 3 Conclusion

In summary, we have proposed a nonferrous ferroptosis‐like bacterial cell death strategy. Compared with the conventional ferrous‐based inducers, the highly potent Ir and Ru SAC inducers with metal–nitrogen–carbon bridging structures possess several advantages. i) They exhibit a highly efficient ROS generation capacity under red light irradiation or in the presence of H_2_O_2_ due to their narrower bandgap and POD‐like nanozyme activity. ii) They can act as a favorable GSHOx‐mimicking nanozyme to enhance endogenous GSH depletion for deactivating GPX4 enzyme and inhibit TrxR levels, resulting in the destruction of antioxidant systems and further accumulation of LPO. iii) They can heavily destroy bacteria's metabolic ability and normal respiration to transform aerobic to anaerobic respiration. iv) They cause severe oxidative stress contributing to impaired bacterial genetic systems and protein lipoylation. Upon irradiation, the Ir and Ru SACs display effective antibacterial activity on Gram‐positive bacteria, Gram‐negative bacteria, clinically isolated MRSA, and can also disrupt biofilms. Both ferroptosis‐like inducers show low toxicity both in vitro and in vivo, suggesting excellent biosafety in potential clinical applications. Finally, the application of Ir and Ru SACs not only significantly accelerated the healing process of wounds and abscesses infected by MRSA and rescued mice with severe infection, but also induced pathogen‐specific immunological memory response to reduce reinfection risk. Overall, our findings first innovatively introduce and validate the nonferrous bacterial ferroptosis‐like death strategy based on COF‐based SACs, which provides a hopeful direction for future anti‐infection therapy.

## Conflict of Interest

The authors declare no conflict of interest.

## Author Contributions

B.S. conceived the idea and designed the project. B.S., X.W., Z.Y., J.Z., and X.C. performed the experiments and analyzed the results. X.W. and Z.Y. assisted with the figure production and experiment design. B.S., X.W., and F.W. wrote and revised the original draft of the manuscript. B.S., N.Z., M.Z., and C.Y. edited the manuscript. J.S. supervised the whole project. All authors discussed the results and commented on the manuscript.

## Supporting information

Supporting Information

## Data Availability

The data that support the findings of this study are available from the corresponding author upon reasonable request.
