# Supplementary material for: Designing Single‐Atom Active Sites on sp2‐Carbon Linked Covalent Organic Frameworks to Induce Bacterial Ferroptosis‐Like for Robust Anti‐Infection Therapy
Source: Adv Sci (Weinh). 2023 Feb 27;10(13):2207507. doi: 10.1002/advs.202207507 (PMC10161020; doi:10.1002/advs.202207507)
Supplement: Supplementary file 1 — Supporting Information [file ADVS-10-2207507-s001.pdf]

Supplementary Information for

Designing Single-Atom Active Sites on sp^2-Carbon Linked Covalent Organic Frameworks to Induce Bacterial Ferroptosis-Like for Robust Anti-Infection Therapy

Baohong Sun, Xinye Wang, Ziqiu Ye, Juyang Zhang, Xiong Chen, Ninglin Zhou, Ming Zhang*, Cheng Yao*, Fan Wu*, Jian Shen*

B. Sun, X. Wang, Z. Ye, J. Zhang, M. Zhang, N. Zhou, J. Shen
National and Local Joint Engineering Research Center of Biomedical Functional Materials
School of Chemistry and Materials Science
Nanjing Normal University
Nanjing 210023, China
E-mail: zhouninglin@njnu.edu.cn (N. Z.); mzhan@dtu.dk (M. Z.)

B. Sun, X. Chen, C. Yao
School of Chemistry and Molecular Engineering
Nanjing Tech University
Nanjing 211816, China
E-mail: yaocheng@njtech.edu.cn (C. Y.)

F. Wu
Key Laboratory of Cardiovascular & Cerebrovascular Medicine
School of Pharmacy
Nanjing Medical University
Nanjing 211166, China
E-mail: wufan71@njmu.edu.cn (F. W.)

J. Shen
Jiangsu Engineering Research Center of Interfacial Chemistry
Nanjing University
Nanjing, 210023, China

Table of Contents

Experimental Procedures **S3**
Materials and Characterizations **S3**
Synthetic Procedures **S4**
Methods **S8**
Results and Discussion **S14**
References **S34**

Experimental Procedures

Materials and Characterizations: Fourier transform infrared (FT IR) spectra were measured with a Nicolet NEXUS670 FTIR spectrometer. X-ray photoelectron spectra (XPS) were performed on a Thermo Fisher ESCALAB Xi X-ray photoelectron spectrometer equipped with Al K*α* monochromatic X-ray source and a micro-focused monochromator. UV-Vis diffuse reflectance absorption spectra (DRS) were recorded on a Shimadzu UV-3600i Plus spectrophotometer. Fluorescence spectra were measured on a JASCO model FP-6600 spectrofluorometer. Fluorescent lifetime was recorded on an FLS1000 spectrometer (Edinburgh Instruments) that contains a 450 W ozone-free xenon arc lamp that covers a range of 230 nm to >1000 nm for steady-state measurements. The fluorescence decay curves were refitted with triple exponential functions, while the intensity-weighted average fluorescence lifetimes were calculated by the equation.

Liquid nuclear magnetic resonance (NMR) spectra were recorded on Bruker Advance 400 at 400 MHz using TMS as an internal standard. Solid-state ^{13}C CP/MAS NMR spectra were performed on a Bruker spectrometer AVANCE III 500 MH using a rotor frequency of 10 kHz. Field-emission scanning electron microscopy (FE-SEM) was performed on a ZEISS SIGMA 500/VP operating at an accelerating voltage of 5.0 kV. The samples were prepared by drop-casting anhydrous acetone suspension onto a mica substrate and coated with gold. High-resolution transmission electron microscopy (HR-TEM) images were recorded on a FEI Talos F200s. Samples were prepared by drop-casting a super-sonicated ethanol suspension of the sp^2c-COFs onto a copper grid. Energy dispersive X-ray analysis (EDX) and elemental mapping were acquired on a FEI Super-X EDS Detector. Spherical aberration-corrected high-angle annular dark-field scanning TEM (SAC-HAADF-STEM) was captured on a FEI Themis Z spherical aberration-corrected transmission electron microscope. Height distributions of samples were determined using a Bruker Dimension Icon atomic force microscope (AFM). The surface potential images and signals of the samples were measured using KPFM (Bruker) under an ambient atmosphere in the amplitude-modulated (AM-KPFM) mode. Quantitative analysis of metal contents was performed by inductively coupled plasma optical emission spectroscopy (ICP-OES) using an Agilent 5110 spectroscopy after dissolving COF samples in acid. Size distribution and zeta potential were detected by using Zetasizer Nano-ZS90 (Malvern). Thermogravimetric analysis (TGA) curves were recorded on a Hitachi Instruments STA7300 with a heating rate of 10 °C/min from 30 °C to 800 °C under argon flow (10 mL/min).

Ir L_3-edge and Ru K-edge analysis was performed with Si(111) crystal monochromators at the BL11B beamlines at the Shanghai Synchrotron Radiation Facility (SSRF) (China). Before the analysis at the beamline, samples were pressed into thin sheets with 1 cm in diameter and sealed using Kapton tape film. The XAFS spectra were recorded at room temperature using a 4-channel Silicon Drift Detector (SDD) Bruker 5040. Ir L_3-edge and Ru K-edge extended X-ray absorption fine structure (EXAFS) spectra were recorded in transmission mode. Negligible changes in the line-shape and peak position of Ir L_3-edge and Ru K-edge XANES spectra were observed between two scans taken for a specific sample. The XAFS spectra of these standard samples (Ir foil, Ru foil, IrO_2, and RuO_2) were recorded in transmission mode. The spectra were processed and analyzed by the software codes Athena and Artemis.

Powder X-ray diffraction (PXRD) data were recorded on a Bruker D8 Advance diffractometer by setting powder on the glass substrate, from 2θ = 2.0° up to 40° with 0.1° increment. Nitrogen gas sorption curves were measured on a Micromeritics ASAP 2020 PLUS HD88 Microporous physical adsorption apparatus. Before measurement, powder samples were degassed in a vacuum at 120 °C for 12 h. The Brunauer-Emmett-Teller (BET) approach was applied to evaluate the surface areas. Pore volume was calculated

from the nitrogen gas sorption curve using the non-local density functional theory (NLDFT) model. Elemental analysis was recorded on a Yanako CHN CORDER MT-6 elemental analyzer. Molecular modeling and Pawley refinement were carried out using Reflex, a software package for crystal determination from XRD pattern, implemented in MS modeling version 4.4 (Accelrys Inc.). Simulated PXRD of AA stacking was performed by the PXRD Diffraction module. Pawley refinement was conducted to optimize the lattice parameters iteratively until the R_P and R_{WP} values converge. The crystalline structures of COFs were determined using the density-functional tight-binding (DFTB+) method including Lennard-Jones (LJ) dispersion. The DFT calculations were performed via the Vienna ab initio simulation Package (VASP), with the projected augmented wave (PAW) method to describe the interaction between the ionic cores and valence electrons, and the Perdew-Burke-Ernzerhof (PBE) generalized gradient approximation (GGA) is used for the exchange-correlation functional. The atomic charges were calculated using the DDEC6 method. Third-order DFTB calculations were also performed using the DFTB+ package to obtain the charges and electrostatic potentials.
CHI660E electrochemical workstation (Chenhua, China) was used to explore the photoelectrochemical properties of as-synthesized samples. All measurements were executed in a three-electrode system containing saturated Ag/AgCl as the reference electrode, Pt foil as the counter electrode, sample-coated FTO glass as the working electrode, and Na_2SO_4 (0.1 M) aqueous solution as the electrolyte. Specifically, as-synthesized samples (5 mg) were ultrasonically dispersed in a mixture (DMF/Nafion solution: 1 mL/50 μL). The resulting slurry was dispensed on the surface of FTO glass.

Synthetic Procedures: Unless otherwise noted, all commercially available reagents were used without further purification. All organic solvents, including N, N-dimethylformamide (DMF), dichloromethane, chloroform, ethanol (EtOH), tetrahydrofuran (THF), 2-ethoxyethanol, cyclohexane, acetone, were purchased from Energy Chemical (Shanghai SAEN Chemical Technology Co., Ltd.). Iridium trichloride, ruthenium chloride, cesium carbonate, and acetic acid (AcOH) were purchased from Sigma-Aldrich. Benzene-1,4-diamine, ammonium thiocyanate, bromine, hydrochloric acid, potassium hydroxide (KOH), malononitrile, ammonia, sulfuric acid, and NH_4Cl were obtained from Sinopharm Chemical Reagent Co., Ltd. (Shanghai, China). 1,1'-(1,4-Phenylene)bis(thiourea), triflic acid, *p*-tolunitrile, acetic anhydride, chromium (VI) oxide, *n*-BuLi, and 1-formylpiperidine were acquired from Energy Chemicals.
2,6-Dicyanomethylbenzo[1,2-d:4,5-d']bisthiazole (TA)[1], 4,4',4''-(1,3,5-triazine-2,4,6-triyl)tribenzaldehyde (BTHAN)[2], dimer [$Ir_2(ppy)_4Cl_2$][3], and dimer [$Ru_2(bpy)_2Cl_2$][4] were synthesized according to reported methods.

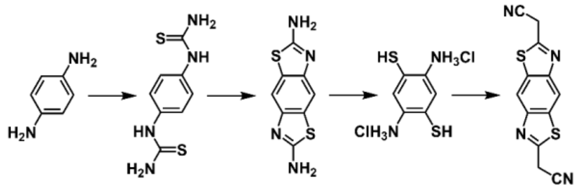

Figure S1. Synthesis of compound 5.

1,1'-(1,4-Phenylene)bis(thiourea) (2). Benzene-1,4-diamine (compound 1, 17.0 g, 157 mmol), hydrochloric acid (30.7 mL, 125 mmol), and activated charcoal (1.1 g, 157 mmol) were added to a 500 mL round bottom flask then heated to 50 °C. The mixture was then filtered through a pad of celite, directly

into another 500 mL flask. Ammonium thiocyanate (48.4 g, 636 mmol) was then added and the reaction was heated at 95 °C for 24 h with stirring. A yellow granular product precipitated during the reaction. The mixture was allowed to cool and the product was filtered and washed with hot water (2 x 40 mL). The yellow granular product was dried in a vacuum desiccator for 48 h and used without further purification (31.9 g, 90 %). ^{1}H NMR (400 MHz DMSO-d_6): δ/ppm = 10.08 (4H, s, NH_2), 7.81-8.05 (4H, m, Ar-H).

Benzo[1,2-d:4,5-d']bis(thiazole)-2,6-diamine (3). A solution of bromine (15.8 mL, 307 mmol) in chloroform (100 mL) was added to a stirred suspension of 1,1'-(1,4-phenylene)bis(thiourea) (30 g, 133 mmol) in chloroform (80 mL) slowly, as to prevent the reaction exceeding 50 °C. The orange slurry was stirred at rt. overnight. The reaction was then heated to reflux for 24 h. The reaction was allowed to cool before being filtered and washed with chloroform (4 x 300 mL). The product was then stirred with 20% sodium bisulfite solution at 90 °C until the orange color subsided. The solid was filtered off as it cool before being dissolved in boiling dilute HCl (1500 mL) and stirred with activated carbon for 3 h before being filtered through celite. Basification with ammonia precipitated a white amorphous solid. The solid was filtered, washed (3 x 100 mL methanol then 100 mL diethyl ether) and then dried in a vacuum desiccator (24.8 g, 84%). ^{1}H NMR (400 MHz DMSO-d_6): δ/ppm = 7.73 (2H, s, Ar-H), 7.44 (4H, s, NH_2).

2,5-Diaminobenzene-1,4-dithiol dihydrochloride (4). Benzo[1,2-d:4,5-d']bis(thiazole)-2,6-diamine (12.0 g, 54.0 mmol) (3) was added to a 250 mL round bottomed flask and the flask was degassed several times. Potassium hydroxide (48.5 g, 864 mmol) was dissolved in another round bottom flask in degassed water (60 mL), then transferred via syringe to the reaction flask. The mixture was stirred for 5 h under reflux and then cooled overnight with stirring. The resulting mixture was filtered under Ar. The yellow precipitate was then dissolved in deaerated water (60 mL) and filtered directly into a flask containing deaerated water (120 mL) and concentrated hydrochloric acid (120 mL). The resulting white crystals were filtered, washed with degassed methanol, and dried under Ar for 1 h before being used immediately in the next step without purification or exposure to air.

2,6-Dicyanomethylbenzo[1,2-d:4,5-d']bisthiazole (5). 10.83 g (62.8 mmol) compound 4, 8.3 g (126 mmol) malononitrile and 7.54 mL (131 mmol) glacial acetic acid were refluxed in 250 mL ethanol for 3 days. The remaining solid was filtered off and washed with ethanol. Vacuum drying yielded 14.68 g (54.3 mmol, 86%) 5 as a grey solid. ^{1}H NMR (400 MHz, DMSO-d_6): δ/ppm = 8.83 (s, 2 H; CH), 4.82 (s, 4 H; CH_2); ^{13}C NMR (100 MHz, DMSO-d_6): δ/ppm = 162.92, 150.70, 134.92, 116.92, 116.56, 23.22.

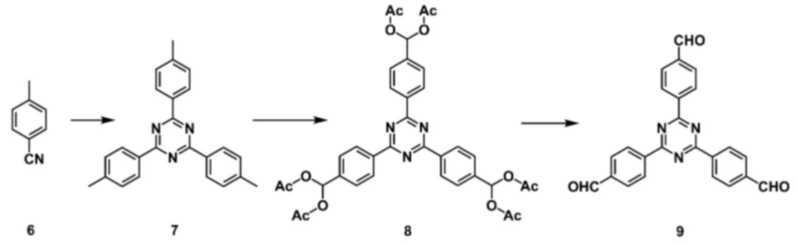

Figure S2. Synthesis of compound 9.

1,3,5-tris-(4-methyl-phenyl)triazine (7). *p*-Tolunitrile (6) was liquefied by putting the storage vessel in a 60 °C drying oven for 30 min. 5.0 mL (8.24 g, 53.8 mmol, 2.15 eq.) of triflic acid (AlfaAesar, 98%) were added to a 25 mL round-bottom Schlenk flask with stir bar and cooled to -20 °C in a Dewar with salt/ice bath (1:3 v/v) under stirring. 3.1 mL (2.99 g, 25.0 mmol, 1.0 eq.) of compound 6 were added dropwise with help of a syringe pump over 1 h. The solution turned into a slurry solid over time and was left for 24 h. The cake was scratched off and transferred in ice water under stirring. This solution was neutralized

with 4-5 mL 25% ammonia. The off-white precipitate was filtered off, washed with acetone (3 x 5 mL) and dried in a vacuum to yield the title compound 7 (2.56 g, 7.29 mmol, 88%).

[4,4',4''-(1,3,5-Triazine-2,4,6-triyl)tris(4,1-phenylene)]-tris(methanetriyl)hexaacetate (8). 100 mg (0.285 mmol, 1.0 eq.) of 7 and 1.00 mL of acetic anhydride were added to a 25 mL round-bottom flask with stir bar and rubber septum and cooled down to -20 °C in a salt/ice bath. After addition of 0.2 mL 98% sulfuric acid, a solution of chromium (VI) oxide (250 mg, 92.6 mmol, 325 eq.) in 1.25 mL acetic anhydride was added to the yellowish solution dropwise by syringe over a period of 3.5 h under stirring. The temperature was kept below 0 °C. The greenish solution was stirred for another hour and then added dropwise to 12.5 mL stirred ice water. The yellowish precipitate was filtered off, washed with dest. water (3 x 3 mL) until neutral and dried in a vacuum. The subsequent further purification by column chromatography (50:1 DCM/EtOAc) on silica gel yielded the title compound 8 (75 mg, 0.107 mmol, 38%).

4,4',4''-(1,3,5-Triazine-2,4,6-triyl)tribenzaldehyde (9). *N*-BuLi (2.5 M in hexane, 1.2 mL, 3.0 mmol, 3.3 eq.) was added dropwise to a suspension of compound 8 (0.50 g, 0.92 mmol, 1.0 eq.) in THF (50 mL) at -78°C. The mixture was stirred at -78°C for 90 min, then 1-formylpiperidine (0.34 mL, 3.0 mmol, 3.3 eq.) was added dropwise at -78°C. The mixture was stirred at -78°C for 30 min and allowed to warm to room temperature. Aqueous concentrated NH_4Cl solution (1 mL) was added and the solvent was removed under reduced pressure. The residue was suspended in a mixture of EtOH (3 mL) and water (3 mL). The suspension after suction filtration was washed with water (10 mL) and EtOH (10 mL) to afford 4,4',4''-(1,3,5-triazine-2,4,6-triyl)tribenzaldehyde (0.35 g, 0.91 mmol, 97 %) as an off-white solid. ^1H NMR (400 MHz, $CDCl_3$): δ/ppm = 10.22 (s, 3H), 8.97-8.99 (d, 6H), 8.16, 8.14 (m, 6H) ppm. ^{13}C NMR (101 MHz, $CDCl_3$): δ/ppm = 191.88, 140.77, 139.32, 129.99, 129.69.

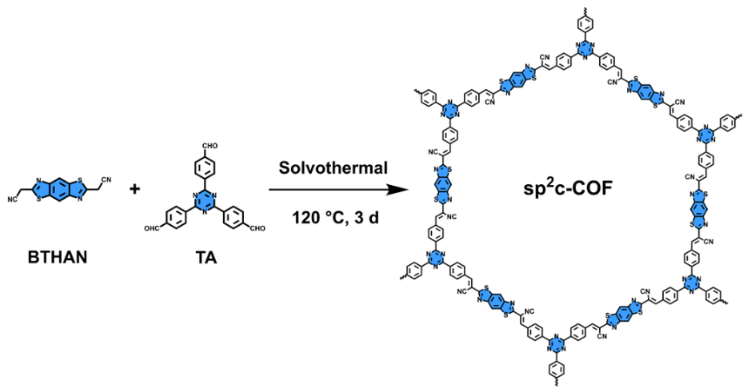

Figure S3. Synthesis of sp^{2}c-COF.

Synthesis of sp^{2}c-COF. A Pyrex tube was charged with TA (compound 5, 12.0 mg, 0.030 mmol), BTHAN (compound 9, 12.4 mg, 0.046 mmol), Cs_2CO_3 (0.1 M, 0.1 mL), tetrahydrofuran (2.0 mL). After being degassed by freeze-pump-thaw technique for three times and then sealed under vacuum, the tube was placed in an oven at 120 °C for 3 d. The resulting precipitate was filtered, washed with tetrahydrofuran, CH_2Cl_2, DMF for 3 d, and dried at 120 °C under vacuum for 12 h. The activated sp^{2}c-COF was obtained as a yellow powder insoluble in common organic solvents (14.9 mg, yield 61.2%) (^{13}C CP/MAS spectra shown as **Figure S6**). Elemental analysis for the calculated: C, 67.54%; H, 2.70%; N, 16.88%; O, 12.88%. Found: C, 68.14%; H, 2.66%; N, 16.52%; O: 12.68%.

The sp^{2}c-COF was poorly soluble in water, which was unfavorable for biomedical applications. To resolve this issue, we decorated the drug surface with methoxy polyethylene glycol amine (mPEG-NH_2, Mw = 4000 Da) via the reaction with aldehyde groups at the COF surface. sp^{2}c-COF (10 mg) dispersed in 50

mL of H_2O were mixed with 50 mg of mPEG-NH_2-4000. After being sonicated for 30 min and stirred for 4 h, excess PEG molecules were removed by centrifugation.

Synthesis of dimer [$Ir_2(ppy)_4Cl_2$]. 2-Phenylpyridine (840 mg, 3.24 mmol) and $IrCl_3$ (440 mg, 1.47 mmol) were placed into a 100 mL Schlenk equipped with a stir bar. Three vacuum-Ar cycles were performed. Then, 18 mL of degassed 2-ethoxyethanol and 6 mL of degassed distilled water were added. The reaction was stirred at 120 °C under Ar atmosphere for 24 hours. After cooling to room temperature, the yellow solid formed was filtrated and gently washed with deionized water and cyclohexane, and finally dried under vacuum. 0.986 g obtained, 77 % yield.

Synthesis of dimer [$Ru_2(bpy)_2Cl_2$]. 2,2′-Bipyridine (1.5 g, 9.64 mmol) and $RuCl_3$ (1 g, 4.82 mmol) were placed into a 100 mL Schlenk equipped with a stir bar. Three vacuum-Ar cycles were performed. Then, 30 mL of degassed ethanol was added. The reaction was stirred at 120 °C under Ar atmosphere for 24 hours. After cooling to room temperature, the purple solid formed was filtrated and gently washed with deionized water and CH_2Cl_2, and finally dried under vacuum. 2.14 g obtained, 86 % yield.

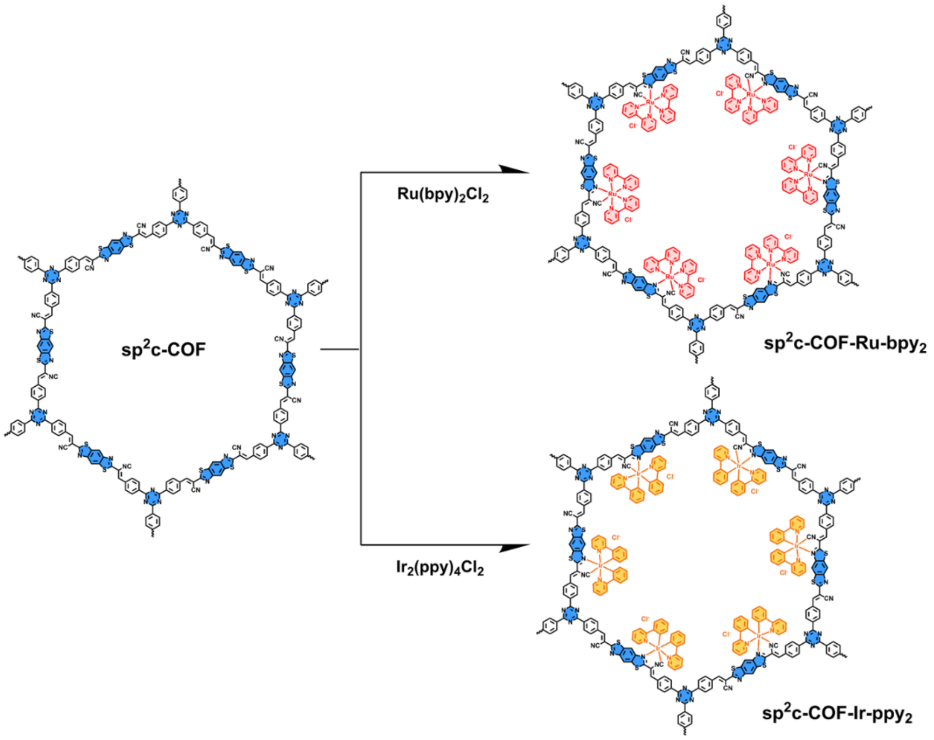

Figure S4. Synthesis of sp^2c-COF-Ir-ppy_2 and sp^2c-COF-Ru-bpy_2.

Synthesis of sp^2c-COF-Ir-ppy_2. The following procedure was done entirely within an argon glovebox with the use of flame-dried glassware as well as dry and degassed solvents. sp^2c-COF (50 mg), dimer [$Ir_2(ppy)_4Cl_2$] (53.6 mg, 0.05 mmol), and CH_2Cl_2 (12 mL) were combined and stirred overnight (24 h). The resulting suspension quickly changed from yellow to dark green during this time. The sp^2c-COF-Ir-ppy_2 solids were then collected by vacuum filtration and washed with dry CH_2Cl_2 (~10 mL). ICP-OES found 5.37 wt% of Ir in the COF. Finally, according to the same operation, we use mPEG-NH_2-4000 to modify the surface of sp^2c-COF-Ir-ppy_2 for enhancing hydrophilia and biocompatibility.

Synthesis of sp^2c-COF-Ru-bpy_2. sp^2c-COF (50.0 mg) and dimer [$Ru_2(bpy)_2Cl_2$] (48.3 mg, 0.1 mmol) were added in a mixture solvent (CH_2Cl_2: methanol = 2: 1, 15.0 mL) and kept at 70 °C for 48 hours with reflux condensation. The sp^2c-COF-Ru-bpy_2 precipitate was collected by filtration and washed with dry CH_2Cl_2 (4 × 10 mL) to afford brown powder. The Ru content in sp^2c-COF was 2.12 wt% as determined

by ICP-OES analysis. Also, we employed mPEG-NH_2-4000 (1 wt%) via the reaction with aldehyde groups at the COF surface following the above-mentioned procedures.

Methods: Kinetic Study, *In Vitro* Antibacterial Study, SEM Observations of Bacteria, TEM Observation of Bacteria, *In Vitro* Live/Dead Bacterial Cell Staining, Cytoplasmic Membrane Potential Assay, Measurement of Intracellular ROS Level, DNA and Protein Assay, Intracellular ROS Imaging, Detection of Lipid Peroxidation (LPO), Detection of MDA, Detection of DNA Degradation, GSH Depletion Capacity, Western Blotting for GPX4, Detection of Intracellular GSH, TrxR, and ATP, Transcriptome Study of MRSA, *In Vitro* Antibiofilm Activity, *In Vivo* Ferroptosis-Like Evaluation, Cytotoxicity Assay, Hemolysis Assay, MRSA-Infected Abscesses Healing Evaluation, Statistical Analysis.

Kinetic Study. a) Detection of·Hydroxyl Radical. The artificial nanozyme activity of the sp^2c-COF-Ir-ppy_2 and sp^2c-COF-Ru-bpy_2 nanocomposites was investigated by monitoring the oxidation of the chromogenic colorless substance 3,3',5,5'-tetramethylbenzidine (TMB) to its intermediate blue colored charge transfer complex oxTMB exhibiting the maximum absorbance at the wavelength of 652 nm. For performing the peroxidase reaction, 1.0 mM of TMB and nanozyme (100 μg/mL) were added to a PBS buffer (pH 7.4) solution. After 30 min incubation and 635 nm irradiation (0.4 W/cm^2, 10 min), the UV–vis absorbance of the resultant oxTMB was monitored at 652 nm wavelength.

b) Detection of Singlet Oxygen. Briefly, 1 mL of sp^2c-COF (100 μg/mL), sp^2c-COF-Ir-ppy_2 (100 μg/mL, containing 1 mM H_2O_2), and sp^2c-COF-Ru-bpy_2 (100 μg/mL) were dispersed in PBS buffer (pH 7.4), respectively. Then, 30 μL of DPBF (1 mg/mL) was added, and the solutions were irradiated for 10 min using a 635 nm laser (0.4 W/cm^2). Immediately, they were centrifuged, and the UV–vis absorption spectra of supernatants were measured.

c) Detection of Superoxide Anion. Briefly, sp^2c-COF, sp^2c-COF-Ir-ppy_2, and sp^2c-COF-Ru-bpy_2 were dissolved in 3 mL of PBS solutions containing DHE (5 μM) with a final concentration of 100 μg/mL, and the above mixtures were cultured for 10 min in dark or irradiation (635 nm, 0.4 W/cm^2) for 10 min. Then, the above mixtures were centrifuged at 10,000 rpm, and the fluorescence of DHE in the liquid supernatant was detected. The fluorescence of DHE in ~600 nm (excitation: 300 nm) was measured.

***In Vitro* Antibacterial Study.** a) Spread Plate Method. Gram-positive *S. aureus* (ATCC 25923), MRSA (from Nanjing Drum Tower Hospital), and Gram-negative *E. coli* (ATCC 25922) were used as model bacteria. The *in vitro* antibacterial effect of various COFs was assessed by the plate counting method. Briefly, bacterial dispersion was diluted to 10^6 CFU/mL with phosphate buffer (PBS, pH 7.4), and subsequently, 0.5 mL of the diluted bacterial suspension was mixed with 0.5 mL of sp^2c-COF, sp^2c-COF-Ir-ppy_2 (containing 2 mM H_2O_2), and sp^2c-COF-Ru-bpy_2 (0, 32, 64, 128, 256, 512 μg/mL). After being treated with and without 635 nm laser irradiation (0.4 W/cm^2, 10 min), the mixture solutions were incubated for another 2 h. Finally, the resulting bacterial suspension was diluted with sterile PBS to 10^4 CFU/mL, and then 40 μL of the diluted bacterial suspension was spread on the solid medium and incubated at 37 °C overnight to count the number of colonies.

b) Broth Microdilution Method. Each sample was divided into four groups: (1) PBS; (2) sp^2c-COF with illumination; (3) sp^2c-COF-Ir-ppy_2 with H_2O_2 (1 mM) and illumination; and (4) sp^2c-COF-Ru-bpy_2 with illumination. The bacterium concentration in the Luria-Bertani (LB) medium was 1 × 10^5 CFU/mL. Different samples were added to achieve a final concentration from 0 to 256 μg/mL in a 96-well plate. The illumination with a wavelength of 635 nm and a power density of 0.4 W/cm^2 was applied for 10 min. All groups were incubated at 37 °C for 12 h with gentle shaking, and then the absorbance at 600 nm was

measured by a microplate reader (Biotek). The inhibition efficiency in solution (η) was determined from the following equation[5]:

$$\eta\ (\%)=\frac{A_1\text{-}A_2}{A_0\text{-}A_3}\times 100\%$$

where A_1, A_2, A_0, and A_3 are the OD600 values of the bacterium culture containing COFs, LB medium containing COFs, the bacterium culture in LB medium, and LB medium alone, respectively. MIC was determined as the lowest concentration of antibacterial materials when no visible growth of bacterium was observed.

SEM Observations of Bacteria. For the observation of SEM, logarithmic growth phase cells of two tested bacterial strains (1×10^6 CFU/mL) were washed with PBS buffer three times and subjected to various treatments (100 μg/mL). The bacteria were immediately fixed with 2.5% glutaraldehyde solution at 4 °C overnight. The fixed bacteria were further dehydrated with a series of ethanol solutions (25%, 50%, 75%, 90%, and 100%). The samples were fixed on an SEM support and dried under a vacuum. The obtained bacterial cells were sputter-coated with gold before being observed using a JSM-5610 LV SEM (JEOL) at an accelerating voltage of 15.0 kV.

TEM Observations of Bacteria[6]. For the observation of TEM, *S. aureus* and *E. coli* bacteria in LB broth medium were washed three times with PBS buffer, suspended in PBS (1×10^6 CFU/mL), and subjected to various treatments (100 μg/mL). The bacterial cells were then pre-fixed with 2.5% glutaraldehyde solution at 4 °C overnight and post-fixed with 1% OsO_4 at 4 °C for 2 h. After that, the fixed bacterial cells were washed thrice with PBS buffer and dehydrated with a S15 series of ethanol solutions (50%, 75%, 90%, 95%, and 100%) and a mixture of ethanol and acetone (v/v = 1:1) for 30 min in each solution. The obtained bacterial cells were treated with a mixture of acetone and epoxide resin (v/v = 1:2) for 12 h and then completely immersed in the epoxy resin for polymerization. Last, the resin products were cut into ~100 nm ultrathin slices and placed on carbon-coated grids. We use a Hitachi H-7650 TEM at a low accelerating voltage to observe the morphological analysis of bacterial cells.

***In Vitro* Live/Dead Bacterial Cell Staining.** The live/dead bacteria with fluorescent labeling were imaged using a Nikon Ti-E-A1R confocal laser scanning microscope (CLSM). Briefly, 500 μL of the mentioned-above bacterial suspensions (10^6 CFU/mL) after various treatments in SEM experiments were treated with 10 μL of SYTO-9/PI double stain kit including propidium iodide (PI, 4.5 μm) and SYTO-9 (4 μM) in the dark for 30 min. In this process, live bacterial cells were stained into green fluorescence with SYTO-9, and dead bacterial cells were stained into red fluorescence with PI. The resulting bacteria samples were placed on a glass slide surface and observed by CLSM.

Cytoplasmic Membrane Potential Assay. The cytoplasmic membrane potential changes were conducted in the presence of a membrane potential-sensitive fluorescent dye of $DiSC_3(5)$. Specifically, mid-logarithmic phase *S. aureus* cells were washed with HEPES buffer (5 mM HEPES, pH 7.4, containing 20 mM glucose), and then diluted to 1×10^7 CFU/mL in HEPES buffer. The cell suspension was incubated with $DiSC_3(5)$ (0.4 μM) until a stable value of fluorescence intensity was achieved. Then KCl was added to a final concentration of 5 mM to equilibrate the cytoplasmic and external K^+ ions. Afterward, the cell suspension and 100 μL of sp^2c-COF, sp^2c-COF-Ir-ppy$_2$, and sp^2c-COF-Ru-bpy$_2$ (100 μg/mL) were mixed with the bacteria in a black 96-well plate to desired concentrations, followed with or without light irradiation (635 nm, 0.4 W/cm^2, 10 min). Changes in fluorescence intensity were recorded with flow cytometry (excitation wavelength: 622 nm; emission wavelength: 670 nm).

Measurement of Intracellular ROS Level. The generation of ROS can be detected by the DCFH-DA probe through the flow cytometry using an excitation of 488 nm, and the emission can be collected

between 580-650 nm. Typically, *E. coli* and *S. aureus* cells (10^6 CFU/mL) were seeded into culture dishes and incubated for 24 h. Then, cells were incubated with DCFH-DA (10 μM in PBS buffer) for another 30 min. After that, each culture dish was rinsed with PBS three times and further incubated with 1 mL of PBS buffer, sp^2c-COF solution (100 μg/mL in PBS buffer), sp^2c-COF-Ir-ppy_2 (100 μg/mL in PBS buffer, containing 1 mM H_2O_2) and sp^2c-COF-Ru-bpy_2 (100 μg/mL in PBS buffer) and irradiated for 10 min. Cells were further rinsed for three times by PBS, and the flow cytometry was measured.

DNA and Protein Assay. After the treatment of sp^2c-COF, sp^2c-COF-Ir-ppy_2, and sp^2c-COF-Ru-bpy_2 with or without 635 nm light irradiation for 10 min, aqueous suspensions of *S. aureus* cells were centrifuged, filtered with a membrane (0.22 μm), and the supernatants were collected. *S. aureus* cells with 1% TritonX-100 and PBS buffer were used as the positive and negative control, respectively. DNA concentrations were quantified by measuring the optical values of the collected supernatants at the wavelength of 260 nm (OD260). The protein contents in the supernatants were measured using an BCA protein assay kit. The absorbance intensity at 562 nm was recorded, and the protein concentrations were calculated against a standard calibration curve using bovine serum albumin (BSA) as a model protein.

Intracellular ROS Imaging. To study the production of hydroxyl radical, singlet oxygen, and superoxide anion in bacterial cells, *E. coli* and *S. aureus* cells (10^6 CFU/mL) were seeded in the 20 mm confocal plate. After incubation for 24 h, the medium was replaced with 1 mL culture medium containing different formulations (sp^2c-COF, sp^2c-COF-Ir-ppy_2, and sp^2c-COF-Ru-bpy_2). After further incubation for 2 h, the cells were treated with or without H_2O_2 (1 mM), stained by APF (10 μM), SOSG probe (40 μM), and DHE (20 μM) for 30 min, and then washed with PBS; the confocal imaging of oxidized APF (Ex: 488 nm, Em: 515 nm), SOSG (Ex: 488 nm, Em: 500–550 nm), and DHE (Ex: 488–535 nm, Em: 610 nm) was immediately recorded on a CLSM (Nikon Ti-E-A1R).

Detection of Lipid Peroxidation (LPO). A C11-$BODIPY^{581/591}$ fluorescence probe was utilized as an indicator to research the LPO level in bacterial cells[7]. Briefly, *E. coli* and *S. aureus* cells were incubated with 100 μg/mL sp^2c-COF, sp^2c-COF-Ir-ppy_2, and sp^2c-COF-Ru-bpy_2 for 2 h, respectively. Subsequently, the illumination groups were taken out and irradiated for 10 min (635 nm, 0.4 W/cm^2). Then, all groups were incubated for another 2 h. Finally, the cells were stained with C11-$BODIPY^{581/591}$ (10 μM) for 30 min followed by CLSM observation.

Detection of MDA. For the measurement of MDA, *E. coli* cells (1×10^8 CFU/mL) were treated with sp^2c-COF, sp^2c-COF-Ir-ppy_2, and sp^2c-COF-Ru-bpy_2 under irradiation. 2×10^5 cells were harvested and washed twice by cold PBS 8 h later after irradiation, and then lysed with RIPA reagent and centrifuged at 12,000 g for 10 min at 4 °C. The collected supernatant was kept on ice. The protein content was determined by BCA assay and equilibrated. 200 μL of 0.37% TBA reagent (diluted with 5% trichloroacetic acid) were added into each well containing 100 μL sample, and the mixture was incubated at 95 °C for 60 min. After cooling down to room temperature in an ice bath for 10 min, 200 μL supernatant (containing MDA-TBA adduct) was taken and added into a 96-well microplate. The plate was immediately measured on a microplate reader at OD 532 nm for colorimetric assay or at Ex/Em = 532/553 nm for fluorometric assay.

Detection of DNA Degradation. The *S. aureus* cells were treated with sp^2c-COF, sp^2c-COF-Ir-ppy_2, and sp^2c-COF-Ru-bpy_2 dispersion, washed twice with 0.1 M PBS (0.1 M, pH 7.4), and dissolved with lysozyme and proteinase K. After that, intracellular DNA was extracted by reference to a Bacterial genomic DNA extraction kit. Finally, the extracted DNA was stained with Gel-Red and identified by gel electrophoresis[8].

GSH Depletion Capacity. The indicator DTNB was utilized to investigate *in vitro* GSH consumption induced by the Ir and Ru SACs. sp^{2}c-COF-Ir-ppy$_2$ (100 μg/mL) and sp^{2}c-COF-Ru-bpy$_2$ (100 μg/mL) were dispersed respectively in PBS (pH 7.4), and GSH (1 mM) was added into the mixing solution and incubated for different time durations. Then, DTNB was added, and the absorbance was determined using UV-vis spectroscopy. In addition, the GSH-depleting property of different COFs (sp^{2}c-COF-Ir-ppy$_2$ and sp^{2}c-COF-Ru-bpy$_2$) was monitored using ^{1}H NMR. In brief, the freshly-prepared sp^{2}c-COF-Ir-ppy$_2$ (100 μg/mL) and sp^{2}c-COF-Ru-bpy$_2$ (100 μg/mL) were respectively added to 5 mL of GSH solution (0.5 mM) at room temperature. After 1 h incubation, 100 μL of this solution was extracted and centrifugated to remove the precipitates. Then, 0.5 mL of D_2O was added to the supernatant solutions. Finally, the solution was measured by ^{1}H NMR spectra.

Western Blotting for GPX4. The *E. coli* cells were cultured overnight. Then, bacterial cells were incubated with 128 μg/mL sp^{2}c-COF, sp^{2}c-COF-Ir-ppy$_2$ (1 mM H_2O_2), and sp^{2}c-COF-Ru-bpy$_2$ and subsequently irradiated with red light for 10 min. Cell lysates were collected and analyzed by denatured polyacrylamide gel electrophoresis (PAGE). Quantification of the results was performed using the Image software.

Detection of Intracellular GSH, TrxR, and ATP. *E. coli* cells were incubated with sp^{2}c-COF, sp2c-COF-Ir-ppy2, and sp2c-COF-Ru-bpy2 (128 μg/mL) for 2 hours and irradiated with 635 nm for 10 min (0.4 W/cm^2). The GSH amount was evaluated using the GSH assay kit (Beyotime); the TrxR activity was measured using the TrxR assay kit (Solarbio), and ATP amount was monitored using the ATP assay kit (Beyotime). In addition, the protein concentration of cellular samples was assayed using the BCA protein assay kit.

Transcriptome Study of MRSA[9]. MRSA was divided into three groups according to different treatments: control group (no treatment), Ir SACs group (cultured with sp^{2}c-COF-Ir-ppy$_2$ and 1 mM H_2O_2 for 2 h and irradiated with 635 nm laser for 10 min), and Ru SACs group (cultured with sp^{2}c-COF-Ru-bpy$_2$ for 2 h and irradiated with 635 nm laser for 10 min). Then, the total RNA of MRSA in the two groups was extracted by a TRIzol kit for RNA sequencing detection. Next, differential gene expression analysis was performed, according to $|\log_2 FC| > 1$ (P value < 0.05). And the data was analyzed through a method including the fastp (v0.20.0, for raw sequencing data filtering), hisat2 (2.1.0, for data comparison with reference genome), stringtie (v2.1.3b, for transcriptome assembly and quantification), Rockhopper (2.0.3, for analysis of new transcripts), RBSfinder (for SD sequence prediction), TransTermHP (v2.09, for terminator prediction), RNAfold (2.4.14, for secondary structure prediction of sRNA), samtools (1.9, for comparison result processing), bcftools (1.9-170-gd7bb95b, for acquisition of variation sites), qualimap (2.2.1, for bam compared the results), pheatmap (1.0.12, for draw heat maps), blast (2.10.1, for gene function annotation), DESeq2 (1.26.0, for repeated differential analysis), and edgeR (3.28.1, for variation analysis). Functions of genes were further analyzed through the Kyoto Encyclopedia of Genes and Genome (http://www.genome.jp/kegg/) and Gene Ontology (http://www.geneontology.org), respectively. All analyses were carried out by Nanjing Jisihuiyuan Biotechnology Co., LTD.

***In Vitro* Antibiofilm Activity.** MRSA cells incubated in LB media were used for evaluation of the therapeutic effects of different treatments against dense biofilm structures. Prepared bacterial suspensions (10^7 CFU/mL) were seeded in confocal dishes for 72 hours of incubation at 37 °C to form biofilms. Then, the biofilms were treated in the Control, sp^{2}c-COF+Laser, sp^{2}c-COF-Ir-ppy$_2$+H_2O_2+Laser, and sp^{2}c-COF-Ru-bpy$_2$+Laser groups, respectively. The concentrations of both sp^{2}c-COF, sp^{2}c-COF-Ir-ppy$_2$, and sp^{2}c-COF-Ru-bpy$_2$ in the above groups are 256 μg/mL. Around 10 min before red light irradiation, sp^{2}c-COF, sp^{2}c-COF-Ir-ppy$_2$ (containing 5 mM H_2O_2), or sp^{2}c-COF-Ru-bpy$_2$ solutions were

applied to biofilms. After 30 min irradiation, 500 μL of SYTO 9 (5.01 μM) was used to stain the biofilms in the dark for 30 min. The biofilm was observed on a Nikon Ti-E-A1R CLSM.

***In Vivo* Ferroptosis-Like Evaluation.** All *in vivo* experiments were conducted under the authority of project and personal licenses granted by the Nanjing Normal University Animal Ethical Management Office. Female BALB/c mice aged 6-8 weeks were purchased from Jiangsu KeyGEN Biotech Corp., Ltd. Each mouse was anesthetized (isoflurane) and shaved, and the round full-thickness cutaneous wounds (~15 mm) were created on their back. Afterward, 10 μL of MRSA cell suspension (1 × 10^8 CFU/mL) was smeared on the wound to build a bacterial infectious model. These mice were randomly divided into seven groups (n = 3): (I) PBS group; (II) sp^2c-COF, (III) sp^2c-COF-Ir-ppy_2, (IV) sp^2c-COF-Ru-bpy_2, (V) sp^2c-COF+Laser, (VI) sp^2c-COF-Ir-ppy_2+H_2O_2+Laser, (VII) sp^2c-COF-Ru-bpy_2+Laser. Each single injection dose contained the same amount of sample (8 mg/mL in PBS buffer, 30 μL). All mice have treated with formulations on days 1 and 3, covered with 3M® Tegaderm dressing, and kept individually with food and drinking water in an SPF animal room. Wound growth was captured by a digital camera on days 0, 3, 5, 8, and 12 for observing the wound-healing process. The wound recovery (%) was calculated by the following equation:

$$\text{Wound recovery (\%)}=\frac{\text{Wound area on certain day}}{\text{Wound area on day 0}}\times 100\%$$

The skin tissue samples of 12 days were collected, fixed, paraffin-embedded, and sliced (Jiangsu KeyGEN Biotech Corp., Ltd.). Meanwhile, partial tissue was homogenized and diluted with deionized water (100-fold). The diluent was plated on LB agar to test the number of bacteria in the wound tissue. The slices were examined by a digital microscope (Jiangnan). H&E, Masson, and Ki67 staining were performed to the determination of histological morphology. Paraffin-embedded wound sections (4 μm-thickness) were incubated with antibodies against H&E, Masson, and Ki67. For Ki67 staining, sections were incubated with a secondary antibody for 1 h. After additional nuclear staining, the coverslips were mounted on slides. The samples were visualized using a microscope, and photomicrographs were taken using a digital camera. Positive indexes were expressed as the percentage change [(number of positive nuclei/total number of nuclei) × 100%]. Besides, the deparaffinized sections were incubated with DCFH-DA and C11-$BODIPY^{581/591}$ antibodies at 4 °C overnight according to the manufacturer's protocols. The samples were photographed through a V5200 tissue panoramic imaging scanning system (Olympus).

Cytotoxicity Assay. 3-(4,5-Dimethylthiazol-2-yl)-2,5-diphenyltetrazolium bromide (MTT) was used to measure the cytotoxicity of each sample. For the MTT experiment, L929 cells (1 × 10^4 cells/mL) and HUVECs (5 × 10^4 cells/mL) were seeded to the 96-well plate (100 μL) and treated with several concentrations (100 μL; 32, 64, 128, 256, and 512 μg/mL) of sp^2c-COF, sp^2c-COF-Ir-ppy_2, and sp^2c-COF-Ru-bpy_2 for 24 h. The cells were washed with PBS buffer and immersed in a 200 μL fresh DMEM medium. 20 μL of MTT solution (5 mg/mL) was added to each well and incubated for 4 h, and finally, 100 μL DMSO was added to dissolve the formazan in the cells. MTT can be reduced by succinate dehydrogenase in the mitochondria of living cells to produce hydrophobic blue-violet formazan. The absorbance at 570 nm was surveyed by a multifunction Synergy 2 microplate reader (BioTek).

Hemolysis Assay. The hemolytic activity of sp^2c-COF, sp^2c-COF-Ir-ppy_2, and sp^2c-COF-Ru-bpy_2 was measured with red blood cells (RBCs). First, 5 mL of whole anticoagulant blood was centrifuged at 3,000 rpm for 6 min to obtain fresh RBCs. The erythrocytes were washed with normal saline and resuspended in normal saline. Afterward, 1.2 mL of saline solution containing various concentrations of samples was mixed with 0.3 mL of the attenuated RBCs suspension. 1.2 mL of Triton X-100 (1%) and saline were mixed with 0.3 mL of the attenuated RBCs suspension to acquire positive and negative controls group,

respectively. All mixtures were maintained at 37 °C for 3.5 h, and then centrifuged at 3,000 rpm for 6 min. The supernatant (100 μL) was transferred to a 96-well plate, and the hemolysis was calculated based on the absorbance at 570 nm using the following equation:

$$\text{Hemolysis (\%)}=\frac{A_S\text{-}A_N}{A_P\text{-}A_N}\times 100\%$$

where A_S is the absorbance of the sample, A_N represents the absorbance of the negative control, and A_P means the absorbance of the positive control.

***In Vivo* Safety Evaluation.** Evaluation of sub-acute toxicity was conducted to assess the probable cumulative toxic effect[10]. Firstly, three COFs at the dose of 2.4 mg/kg were injected (*i.p.*) into mice (n = 3 each), every 24-h for three consecutive days (total of 3 injections). During the study period, animals were monitored daily for mortality, changes in their fur, eyes, and behavioral signs (salivation, tremors, convulsions, diarrhea, and lethargy). Finally, animals were anesthetized with ketamine-xylazine (100:10 mg/kg *i.p.*), and blood samples were collected via cardiac puncture. Blood samples of half of the mice from each group were collected into tubes containing no anticoagulants. Samples were allowed to clot, centrifuged (3000 g for 15 min), and sera were obtained for blood chemistry. Biochemical evaluation of creatinine, urea, aspartate transaminase (AST), and alanine transaminase (ALT) were performed. The blood samples of another half of the mice from each group were collected in ethylene diamine tetra-acetic acid (EDTA) tubes for hematological evaluation. Immediately after the blood collection, the heart, liver, spleen, lung, and kidney were dissected out, weighed, and fixed in 4 % paraformaldehyde solution for histological evaluation.

MRSA-Infected Abscesses Healing Evaluation. Animal experiments were performed according to the protocols approved by the Animal Ethics Committee of Nanjing Normal University (IACUC-20220265) and complied with the Animal Care and Use Committee guidelines. Female BALB/c mice (six-week-old, ≈18 g) were used in all animal experiments, and they were randomly divided into seven groups: (I) PBS group; (II) sp^2c-COF, (III) sp^2c-COF-Ir-ppy_2, (IV) sp^2c-COF-Ru-bpy_2, (V) sp^2c-COF+Laser, (VI) sp^2c-COF-Ir-ppy_2+H_2O_2+Laser, (VII) sp^2c-COF-Ru-bpy_2+Laser. Before being subcutaneously injected into the skin of the back, the mouse was anesthetized by intraperitoneal injection of 4% chloral hydrate (10 mL per 1 kg body weight). Then, ~ 50 μL of MRSA (10^8 CFU/mL) was injected, and the mice model of severe abscess infection was successfully established. Then, the mice received 50 μL of COFs (100 μg/mL) by subcutaneous injection on days 1 and 3. Each group received the corresponding treatment listed above. The abscess conditions of the tissue were photographed and recorded every day. On the last day (12th day), mice were euthanized, and the skin tissues at the infected wound sites were dissected. The residues of bacteria in the surrounding tissues of various infected abscesses were further evaluated by plate counting. After being fixed with a 4% paraformaldehyde solution, the skin tissues were used to perform the Wright, H&E, staining, and Masson staining. On the 12th day, the infected tissues of the mice were collected, fixed with 4% paraformaldehyde, embedded in paraffin, and sliced into 5 μm thick tissue sections. For histological analysis, sections were stained with H&E, Wright, and Masson trichrome stains after deparaffinization and rehydration. For immunohistochemical staining, the tissue sections were placed in a citrate antigen retrieval buffer (pH 6.0) for antigen retrieval. Then the tissue sections were sealed with 3% BSA for 30 min. After the sealing solution was shaken off, tissues were incubated with diluted CD31, VEGF, TNF-α, IL-1β, and IL-6 as the primary antibody at 4 °C overnight. The DAB solution was used for tissue color development and photographed with an optical microscope (BM2100, Jiangnan). For immunofluorescence staining, deparaffinized and rehydrated sections were heat-induced for antigen retrieval in citrate buffer (10×10^{-3} M, pH 6.0) at 98 °C for 10 min. After infiltration, 10% goat

serum was used to block non-specific binding for 1 h and 10 min. To assess the level of macrophages, the rabbit anti-CD80 and anti-CD163 primary antibody was used, followed by incubation with goat anti-rabbit secondary antibody. The nuclei were counterstained with DAPI for 10 min. A fluorescence microscope (Olympus, Japan) was used to acquire immunofluorescence images, and Image J software was used for the quantification. On day 26, to detect the amount of memory B cells, cells from blood samples with the removal of red blood cells by the red blood cell lysate were incubated with anti-CD19-APC, anti-CD21-FITC, and anti-CD45-PE. The excess antibodies were washed away with FACS Buffer before the flow cytometry assay. Data analysis was carried out using FlowJo software.

Statistical Analysis. All the experimental data were statistically analyzed, and the results were expressed as a mean standard deviation (SD). Statistical differences were determined using one-way ANOVA followed by a Bonferroni post hoc test for multiple comparisons with SPSS, version 24 (IBM). In all cases, differences were considered significant if $P<0.05$.

Results and Discussion

Figures:

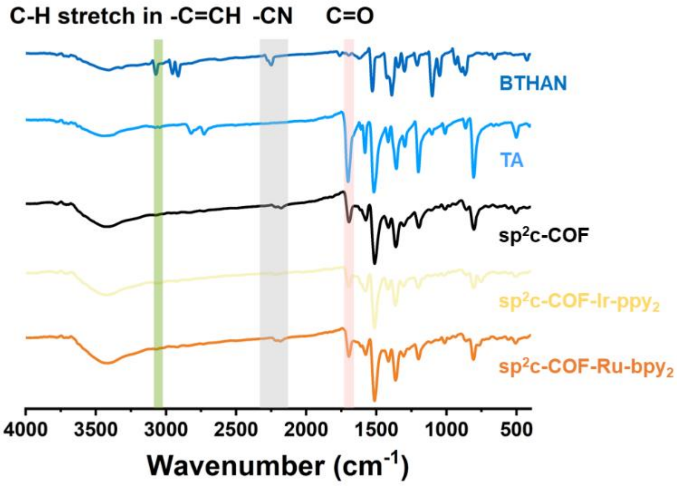

Figure S5. FT IR spectra of BTHAN, TA, sp^{2}c-COF, sp^{2}c-COF-Ir-ppy$_2$, and sp^{2}c-COF-Ru-bpy$_2$.

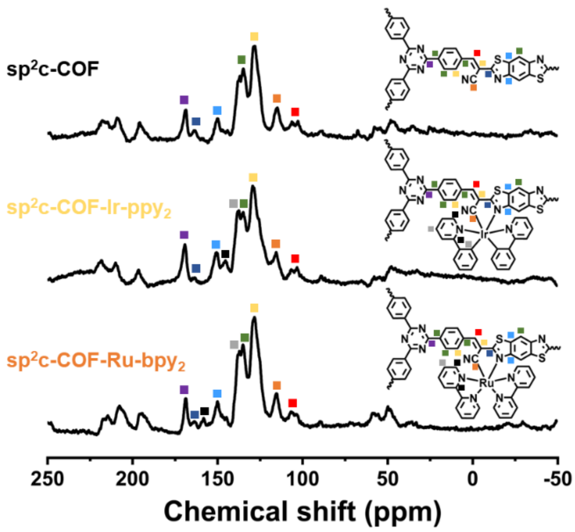

Figure S6. Solid-state ^{13}C CP-MAS NMR spectra of sp^{2}c-COF, sp^{2}c-COF-Ir-ppy$_2$, and sp^{2}c-COF-Ru-bpy$_2$.

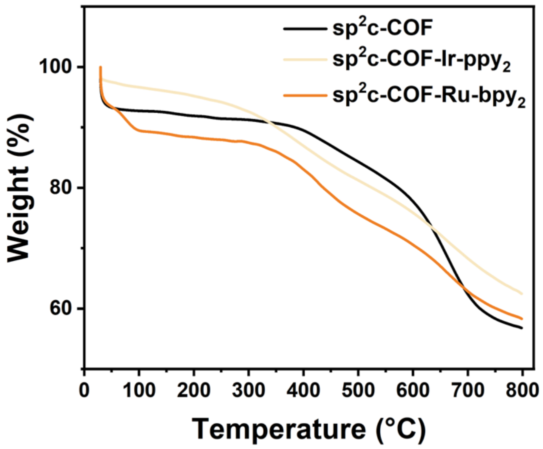

Figure S7. TGA curves of sp^{2}c-COF, sp^{2}c-COF-Ir-ppy$_2$, and sp^{2}c-COF-Ru-bpy$_2$.

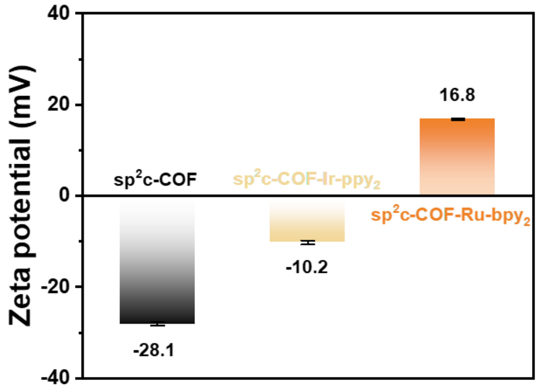

Figure S8. Zeta potential of sp^{2}c-COF, sp^{2}c-COF-Ir-ppy$_2$, and sp^{2}c-COF-Ru-bpy$_2$. Data are expressed as mean ± SD; n = 3.

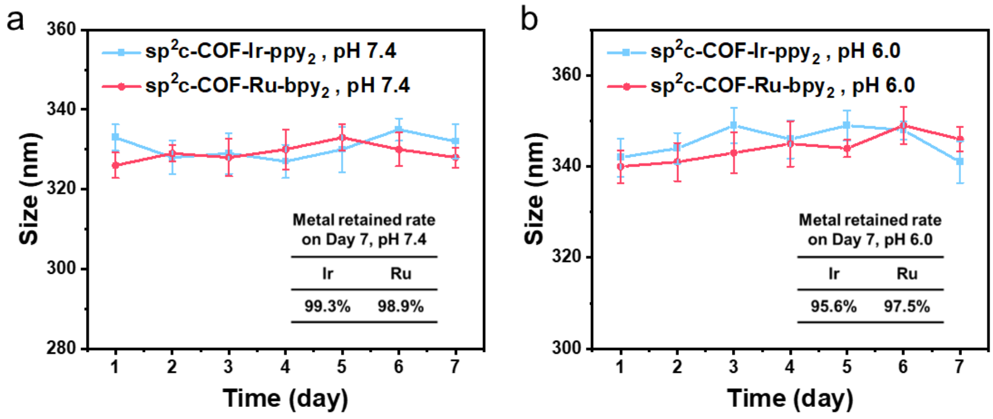

Figure S9. a-b) Stability of sp^{2}c-COF-Ir-ppy$_2$ and sp^{2}c-COF-Ru-bpy$_2$ in PBS (0.1 M, pH 7.4) and PBS (0.1 M, pH 6.0) by monitoring particle size. The inset is the corresponding Ir or Ru retained on the materials on day 7 measured by ICP-OES. Data are expressed as mean ± SD; n = 3.

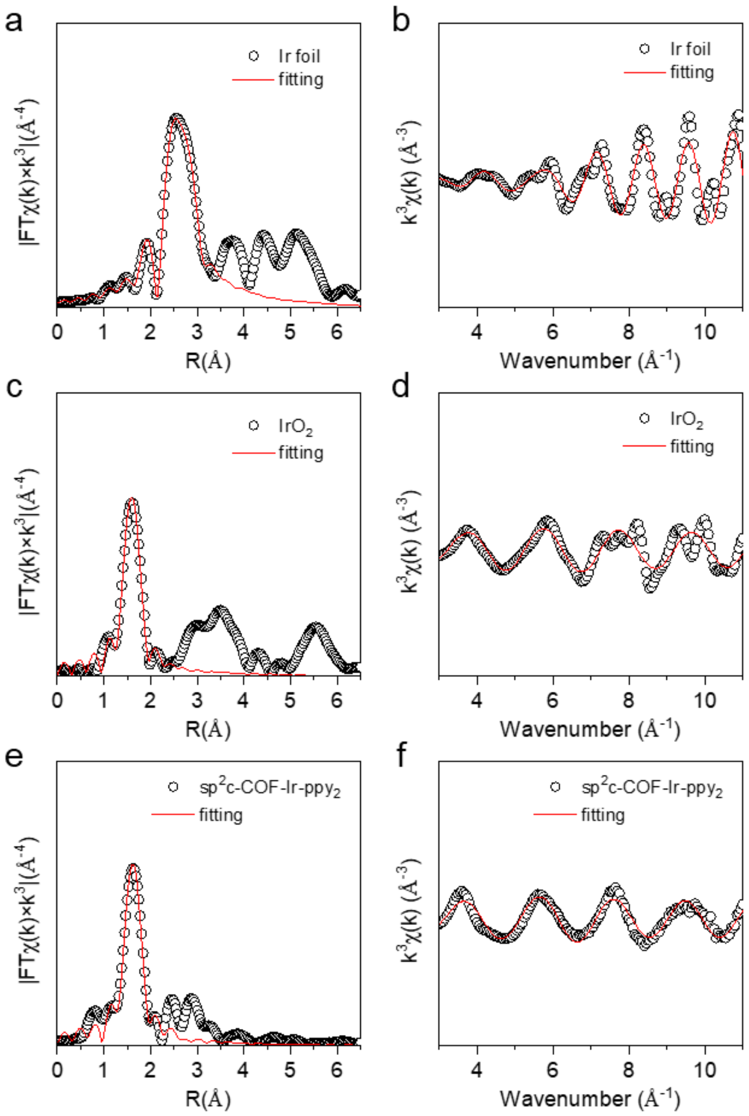

Figure S10. a-f) The fitting of the FTEXAFS spectra of Ir foil, IrO_2, and sp^2c-COF-Ir-ppy_2.

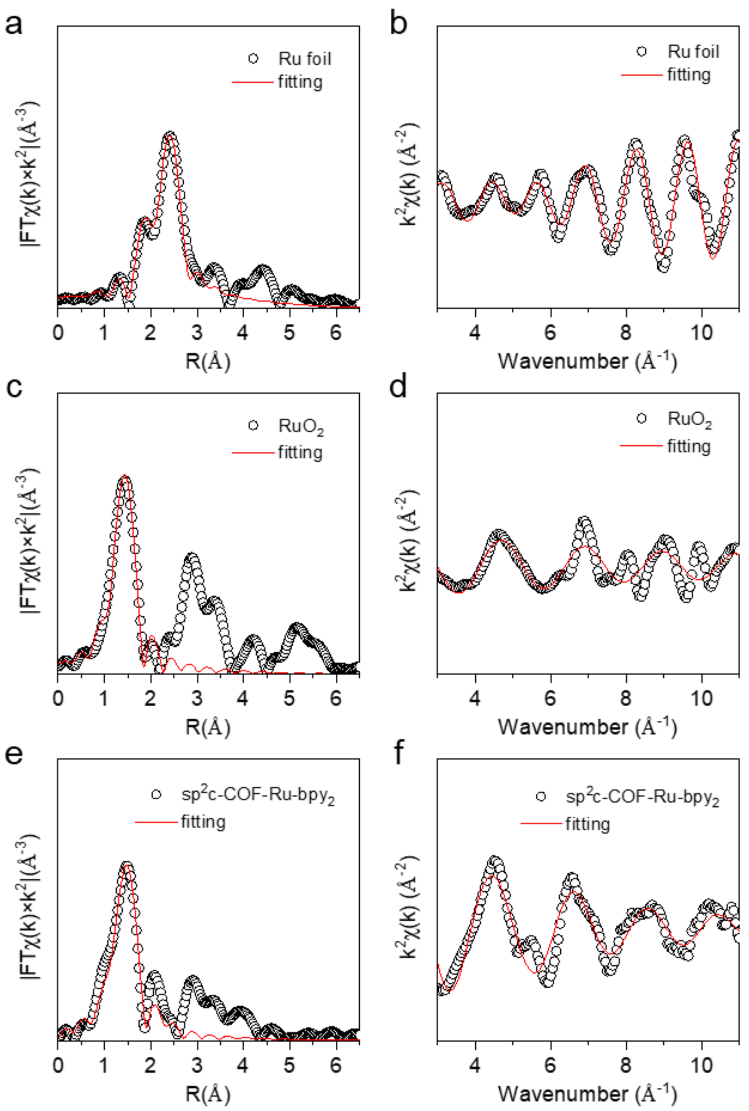

Figure S11. a-f) The fitting of the FTEXAFS spectra of Ru foil, RuO_2, and sp^{2}c-COF-Ru-bpy$_2$.

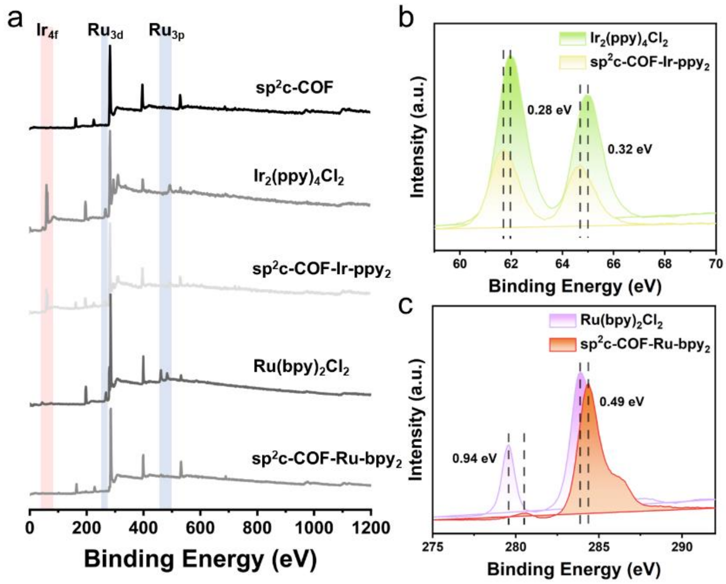

Figure S12. a) XPS spectra of sp^{2}c-COF, $Ir_2(ppy)_4Cl_2$, sp^{2}c-COF-Ir-ppy$_2$, $Ru(bpy)_2Cl_2$, and sp^{2}c-COF-Ru-bpy$_2$. b) High-resolution XPS spectra in Ir 4f regions of the $Ir_2(ppy)_4Cl_2$ and sp^{2}c-COF-Ir-ppy$_2$. c) High-resolution XPS spectra in Ru 3d regions of the $Ru(bpy)_2Cl_2$ and sp^{2}c-COF-Ru-bpy$_2$.

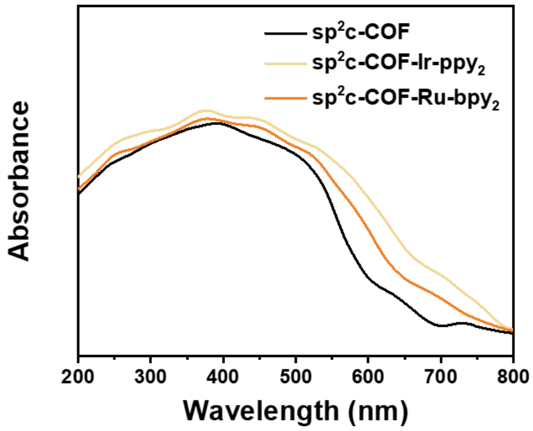

Figure S13. UV–vis DRS spectra of sp^{2}c-COF, sp^{2}c-COF-Ir-ppy$_2$, and sp^{2}c-COF-Ru-bpy$_2$.

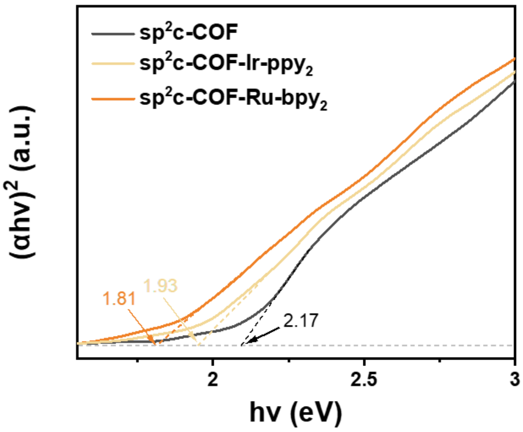

Figure S14. The Tauc plots and corresponding bandgap of sp^{2}c-COF, sp^{2}c-COF-Ir-ppy$_2$, and sp^{2}c-COF-Ru-bpy$_2$.

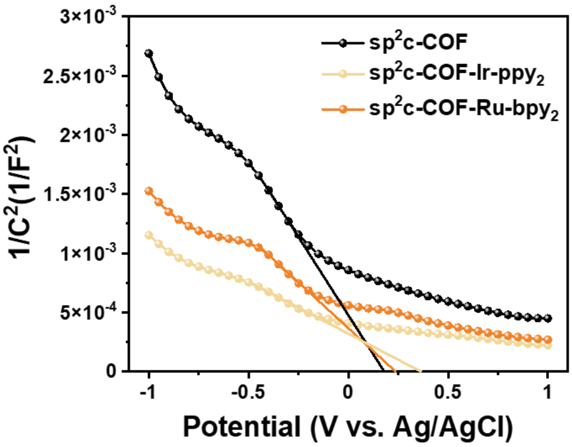

Figure S15. Mott−Schottky (MS) plots of sp^{2}c-COF, sp^{2}c-COF-Ir-ppy$_2$, and sp^{2}c-COF-Ru-bpy$_2$. Three COFs showed the characteristic of the *p*-type semiconductor.

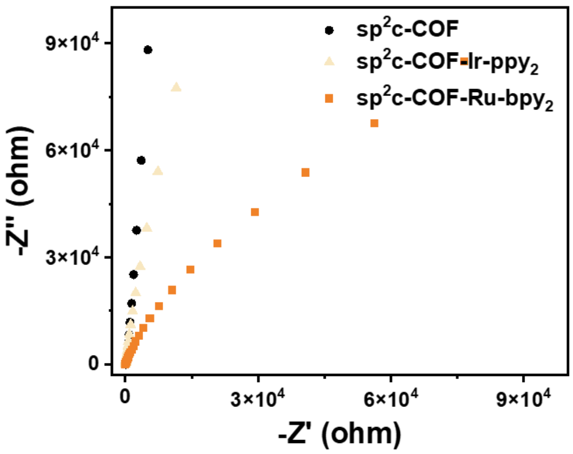

Figure S16. EIS plots of sp^{2}c-COF, sp^{2}c-COF-Ir-ppy$_2$, and sp^{2}c-COF-Ru-bpy$_2$.

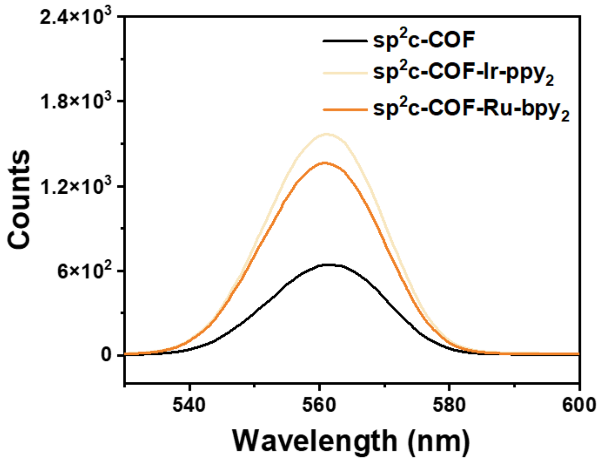

Figure S17. Fluorescence emission spectra of sp^{2}c-COF, sp^{2}c-COF-Ir-ppy$_2$, and sp^{2}c-COF-Ru-bpy$_2$.

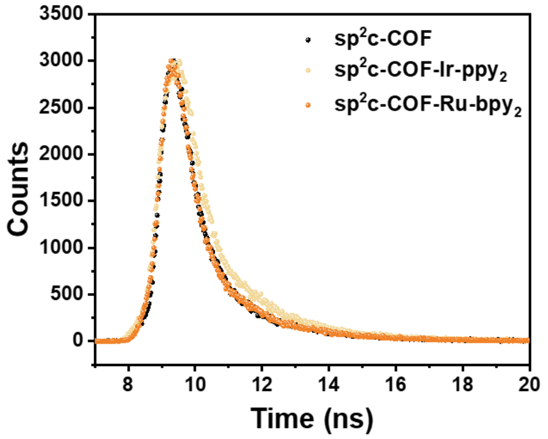

Figure S18. Transient fluorescence spectra of sp^2c-COF, sp^2c-COF-Ir-ppy_2, and sp^2c-COF-Ru-bpy_2.

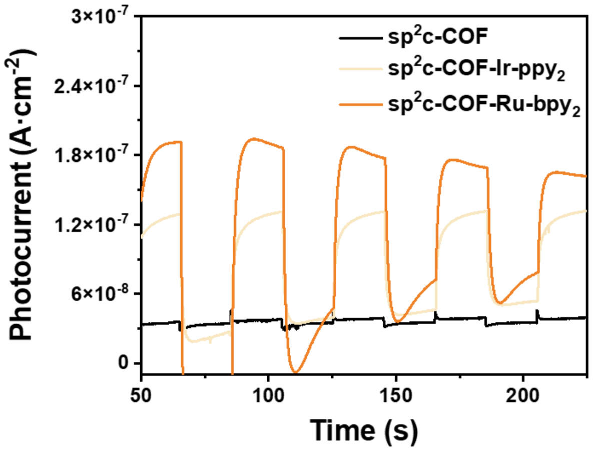

Figure S19. Photocurrent densities of sp^2c-COF, sp^2c-COF-Ir-ppy_2, and sp^2c-COF-Ru-bpy_2.

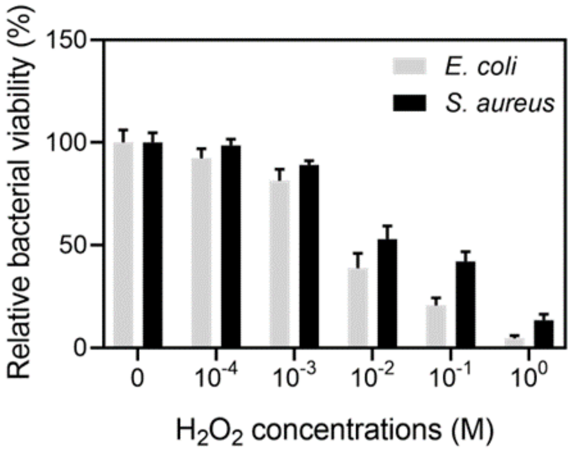

Figure S20. Relative viability of bacterial cells in the presence of different concentrations of H_2O_2. Data are expressed as mean ± SD; n = 3.

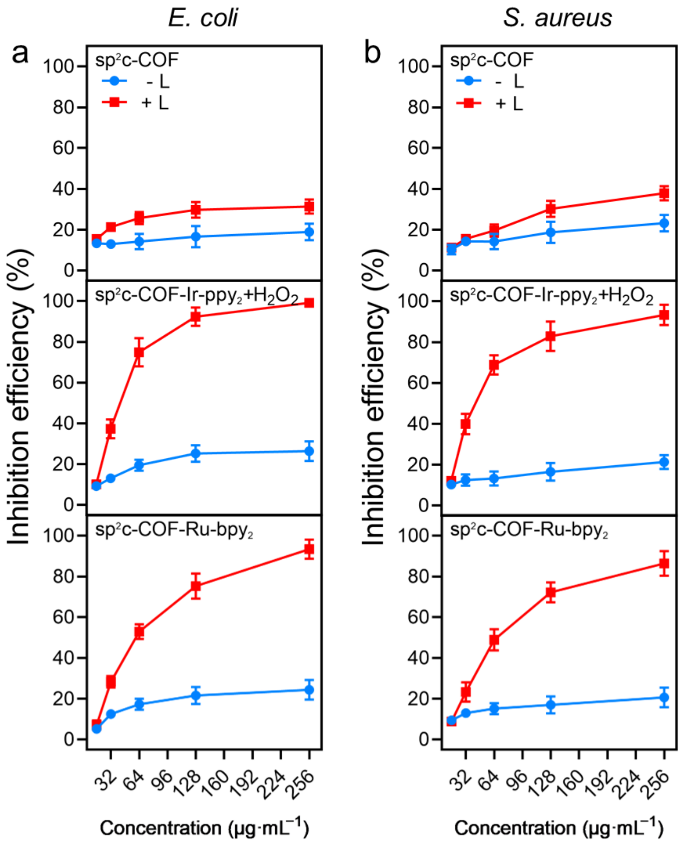

Figure S21. Inhibition efficiency of antibacterial samples for a) *E. coli* and b) *S. aureus*. Data are expressed as mean ± SD; n = 3.

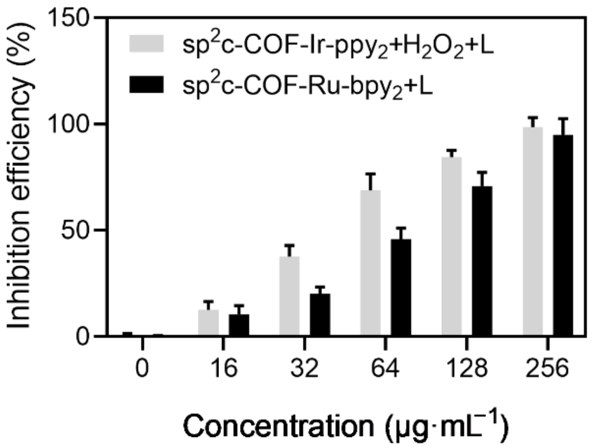

Figure S22. Antibacterial activity against MRSA determined by CFU assay of aqueous dispersions of sp^{2}c-COF-Ir-ppy$_2$ and sp^{2}c-COF-Ru-bpy$_2$ with 635 nm light irradiation for 10 min (0.4 W/cm^2). Data are expressed as mean ± SD; n = 3.

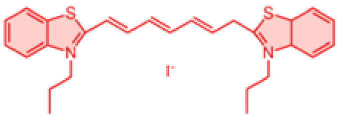

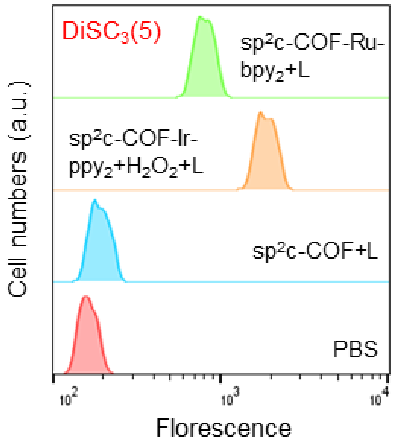

Figure S23. Fluorescent changes of DISC3(5) probe upon incubation with *S. aureus* cells in the presence of sp²c-COF, sp²c-COF-Ir-ppy2, and sp²c-COF-Ru-bpy2.

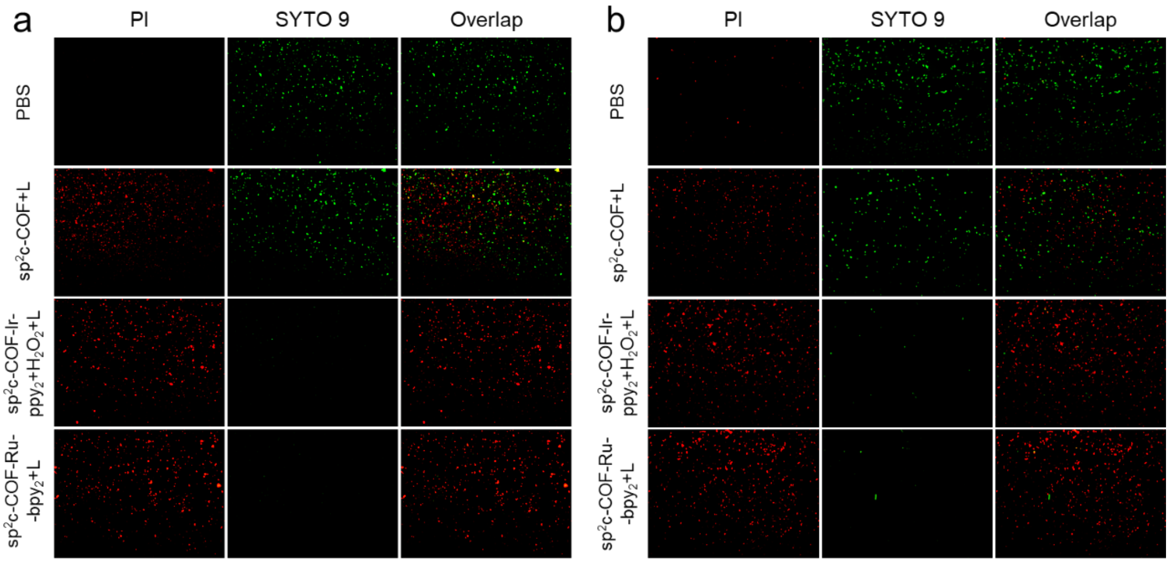

Figure S24. CLSM images of (a) *S. aureus* and (b) *E. coli* cells upon incubation with sp²c-COF, sp²c-COF-Ir-ppy2, and sp²c-COF-Ru-bpy2 using SYTO-9 and PI probe.

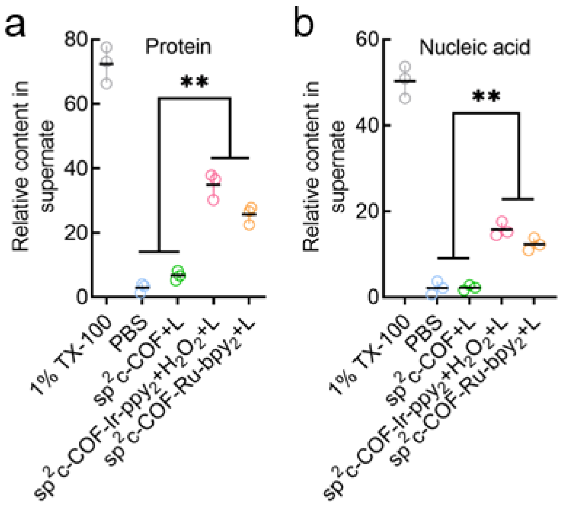

Figure S25. a) BCA protein assay, and b) nucleic acid leakage assay of *S. aureus* cells in the presence of sp²c-COF, sp²c-COF-Ir-ppy$_2$, and sp²c-COF-Ru-bpy$_2$ with red light irradiation. Data are expressed as a scatter plot (show all points); n = 3. The significant differences between data were assessed by one-way ANOVA with Bonferroni's comparison test, giving *P* values, * denotes *P* < 0.05, ** denotes *P* < 0.01.

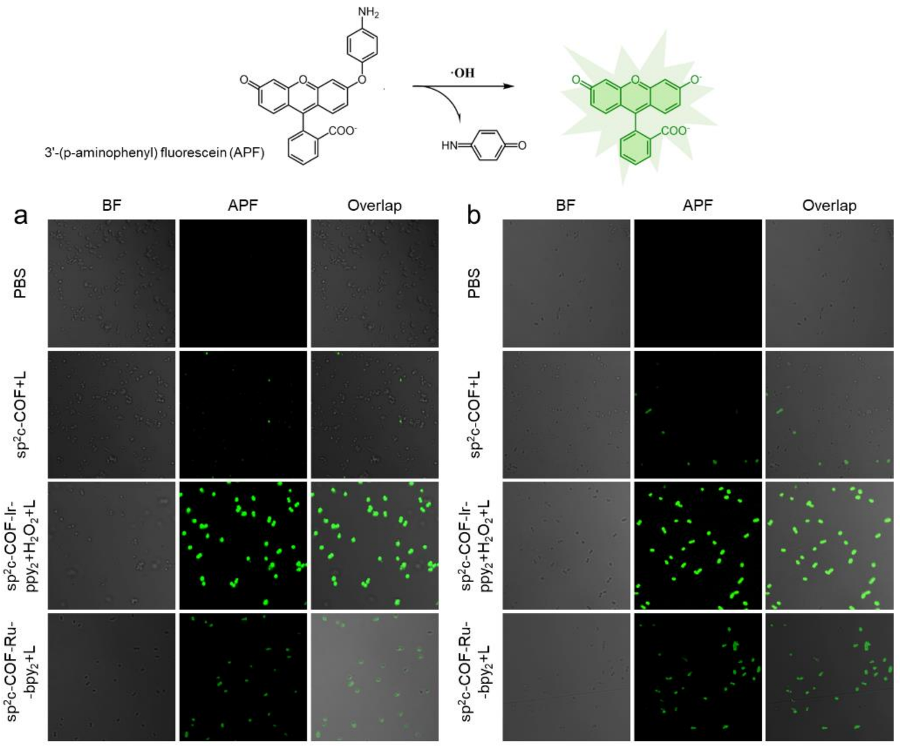

Figure S26. CLSM images of (a) *S. aureus* and (b) *E. coli* cells upon incubation with sp²c-COF, sp²c-COF-Ir-ppy$_2$, and sp²c-COF-Ru-bpy$_2$. APF probe was used for the detection of •OH.

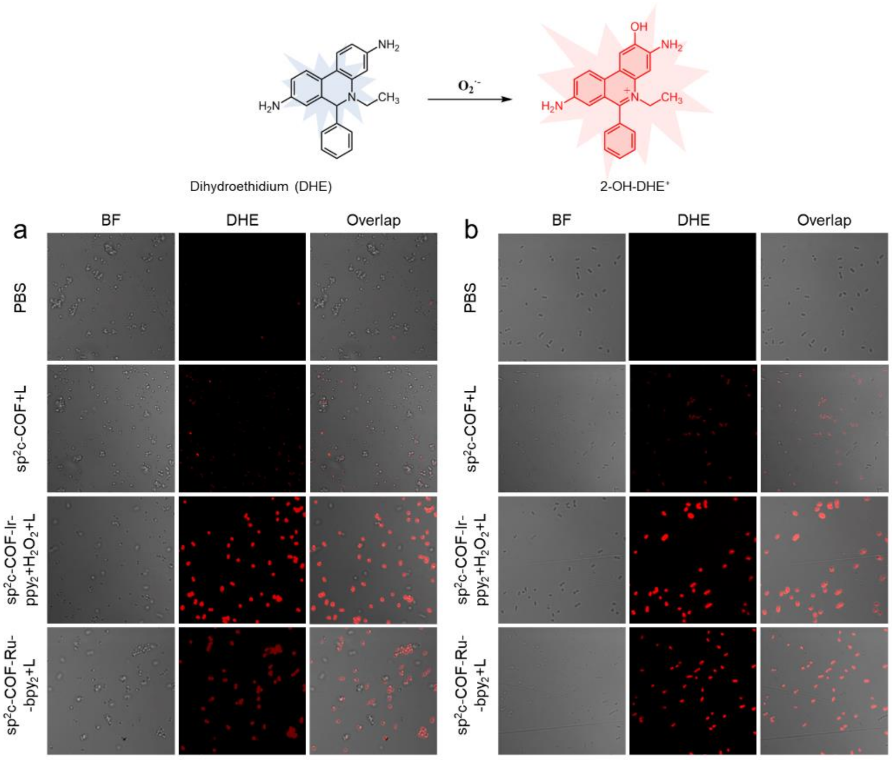

Figure S27. CLSM images of (a) *S. aureus* and (b) *E. coli* cells upon incubation with sp^{2}c-COF, sp^{2}c-COF-Ir-ppy$_2$, and sp^{2}c-COF-Ru-bpy$_2$. DHE probe was used for the detection of $O_2^{\cdot-}$.

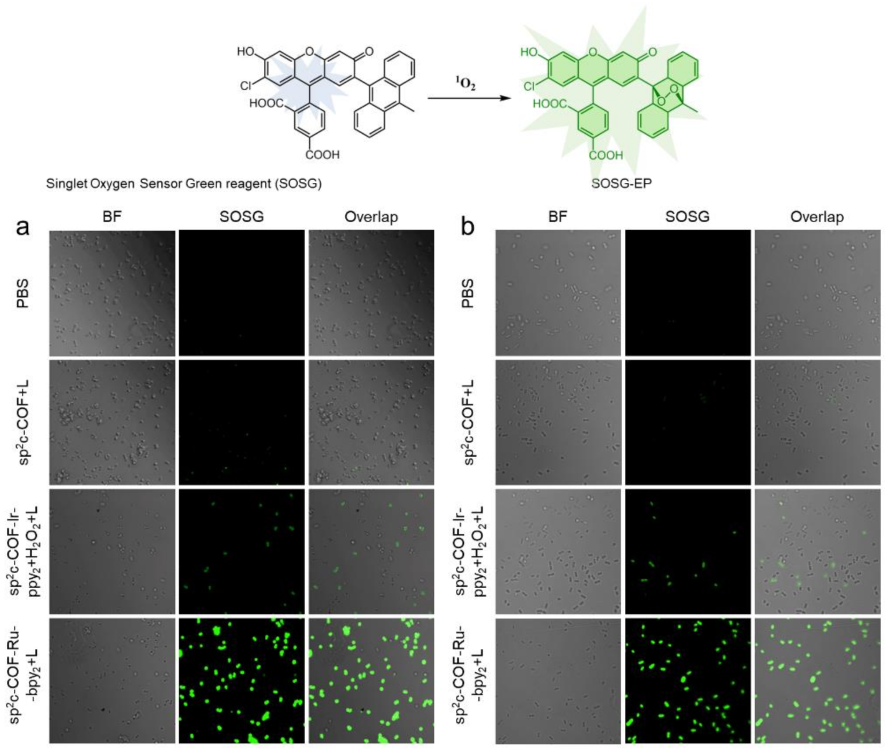

Figure S28. CLSM images of (a) *S. aureus* and (b) *E. coli* cells upon incubation with sp^{2}c-COF, sp^{2}c-COF-Ir-ppy$_2$, and sp^{2}c-COF-Ru-bpy$_2$. SOSG probe was used for the detection of $^1O_2$.

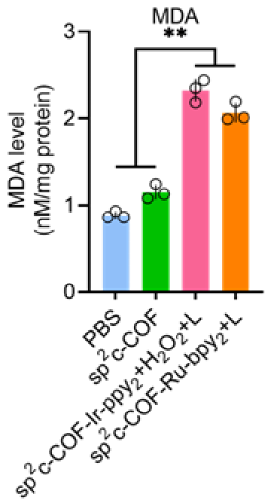

Figure S29. The analysis of MDA extracted from *S. aureus* treated with sp[2]c-COF, sp[2]c-COF-Ir-ppy$_2$, and sp[2]c-COF-Ru-bpy$_2$. Data are expressed as a scatter plot (show all points); n = 3. The significant differences between data were assessed by one-way ANOVA with Bonferroni's comparison test, giving *P* values, * denotes $P < 0.05$, ** denotes $P < 0.01$.

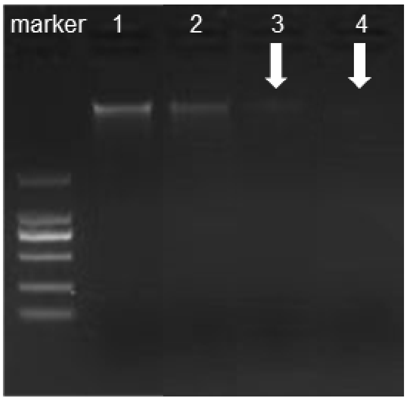

Figure S30. Gel electrophoresis of DNA extracted from *S. aureus* treated with sp[2]c-COF, sp[2]c-COF-Ir-ppy$_2$, and sp[2]c-COF-Ru-bpy$_2$.

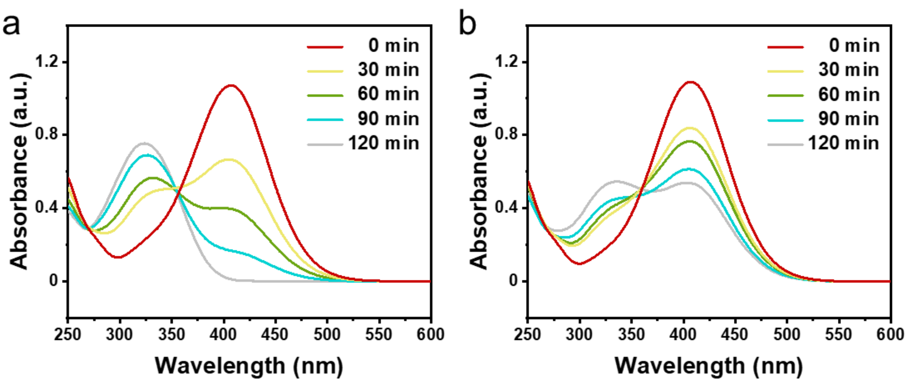

Figure S31. GSH depletion by a) sp[2]c-COF-Ir-ppy$_2$ and b) sp[2]c-COF-Ru-bpy$_2$ presented by absorbance of DTNB.

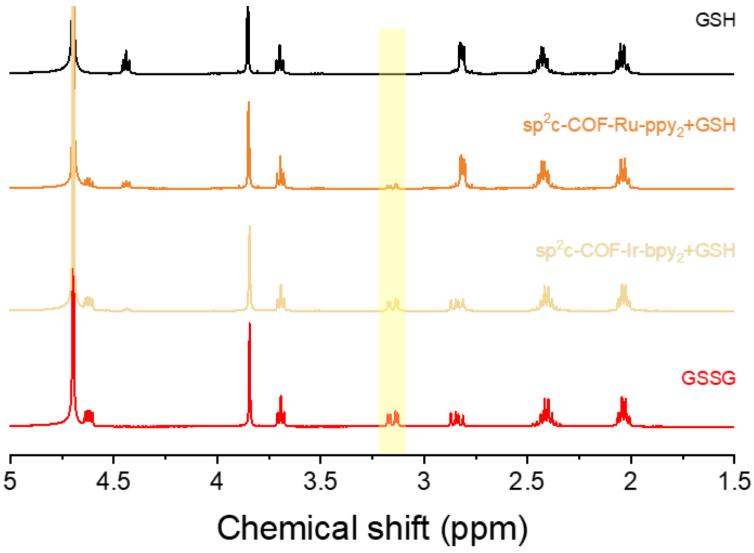

Figure S32. ^{1}H NMR spectra of GSH, GSH + sp[2]c-COF-Ir-ppy2 (1 h), GSH + sp[2]c-COF-Ru-bpy2 (1 h), and commercial GSSG.

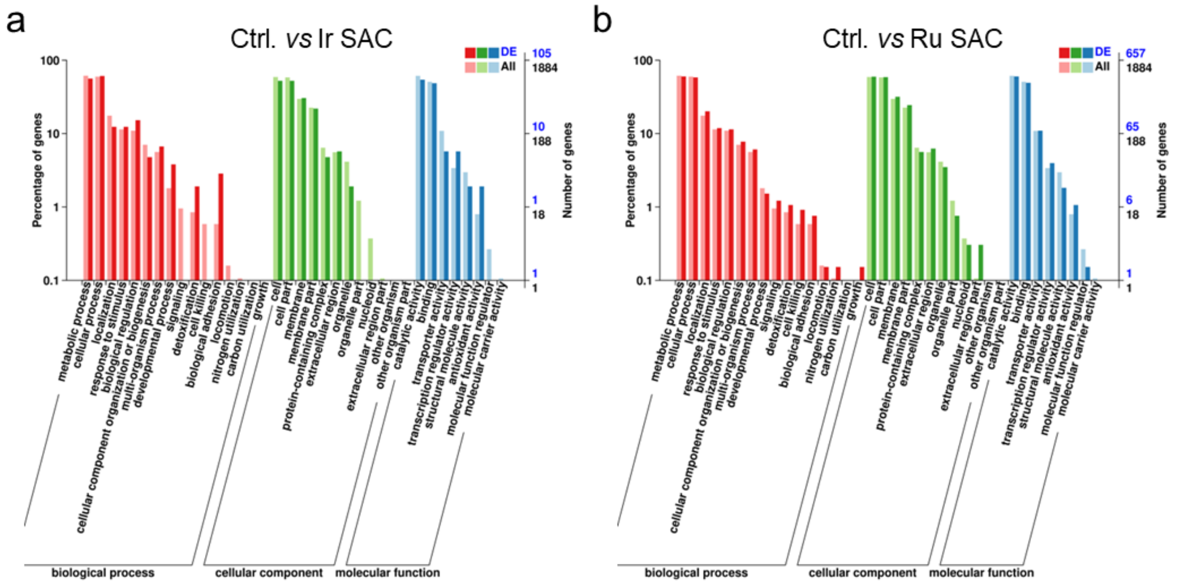

Figure S33. GO enrichment analysis of the gene functions of upregulated and downregulated DEGs in a) Ir SAC and b) Ru SAC compared with Ctrl.

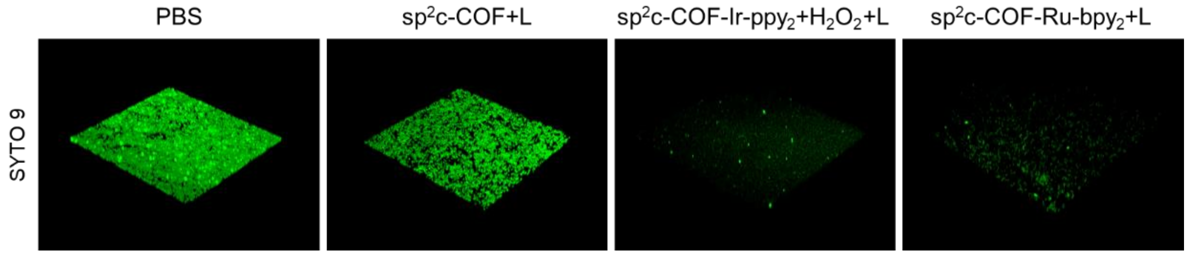

Figure S34. Three-dimensional reconstructions of the fluorescence-labeled MRSA biofilms stained with SYTO 9 after different treatments.

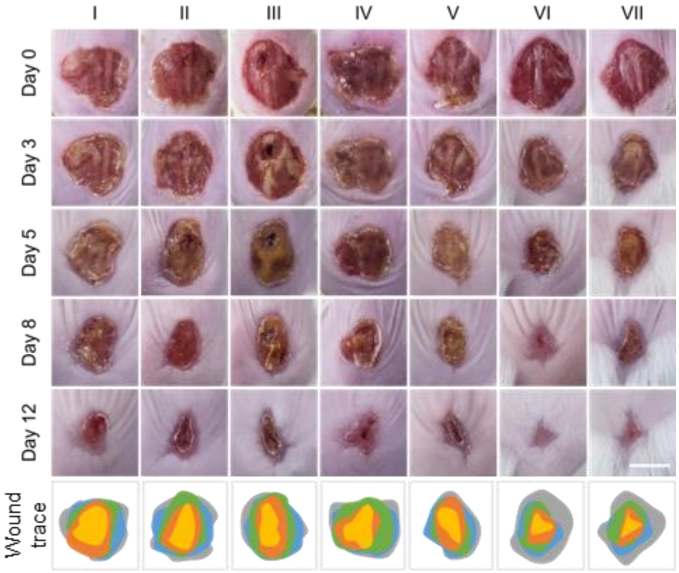

Figure S35. Photographs of wounds in the differently treated groups on days 0, 3, 5, 8, and 12. n = 3. Scale bar = 12 mm.

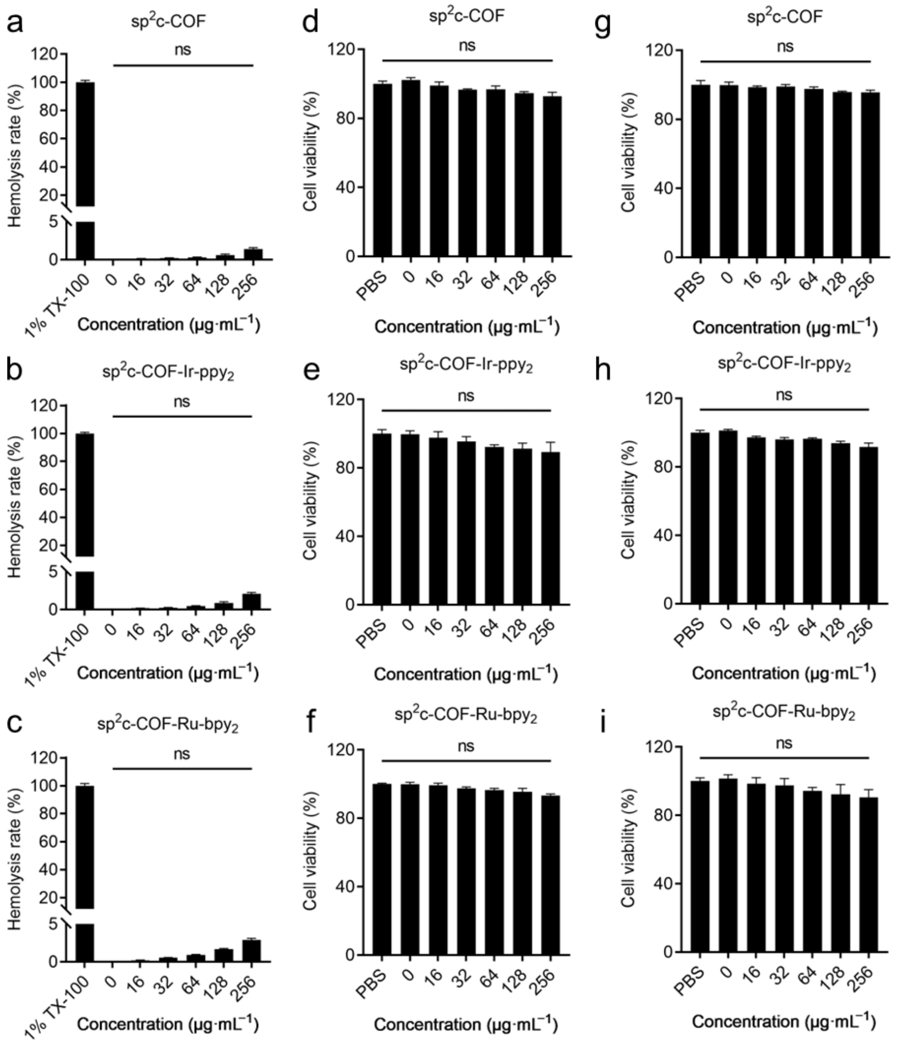

Figure S36. a-c) Hemolytic assay of a) sp^2c-COF, b) sp^2c-COF-Ir-ppy_2, and c) sp^2c-COF-Ru-bpy_2, respectively. 1% Triton X-100 and PBS were used as the positive and negative control, respectively. d-f) Cell viability of L929 cells as determined by MTT assay of aqueous dispersions of d) sp^2c-COF, e) sp^2c-COF-Ir-ppy_2, and f) sp^2c-COF-Ru-bpy_2. g-i) Cell viability of HUVECs as determined by MTT assay of aqueous dispersions of g) sp^2c-COF, h) sp^2c-COF-Ir-ppy_2, and i) sp^2c-COF-Ru-bpy_2. Data are expressed as mean ± SD; n = 3. The significant differences between data were assessed by one-way ANOVA with Bonferroni's comparison test, giving *P* values, * denotes $P < 0.05$, ** denotes $P < 0.01$.

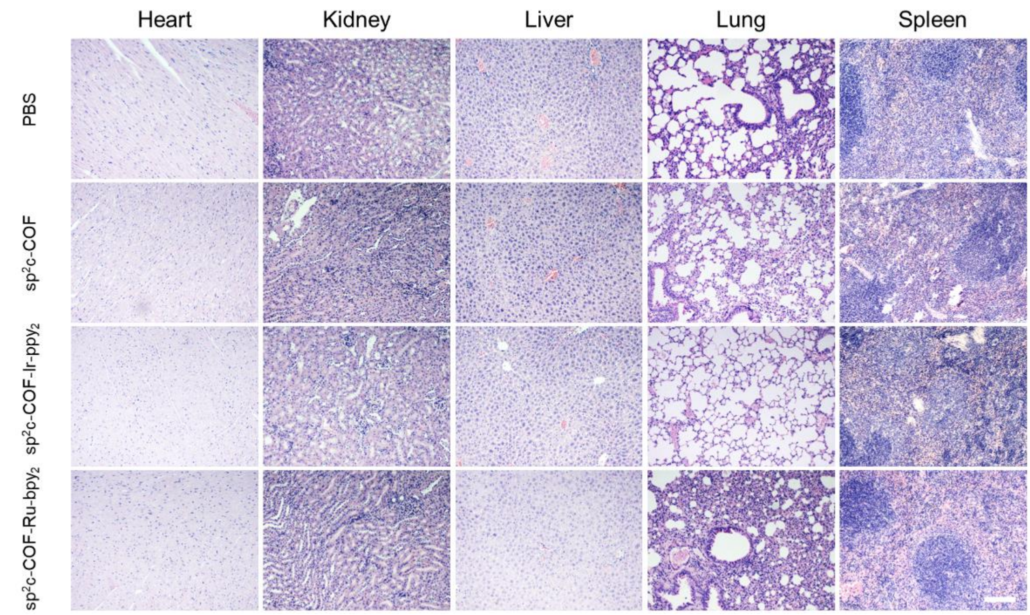

Figure S37. Histological examination of major organs on day 3 after the intraperitoneal injection of sp^2c-COF, sp^2c-COF-Ir-ppy$_2$, and sp^2c-COF-Ru-bpy$_2$ (scale bar = 100 μm).

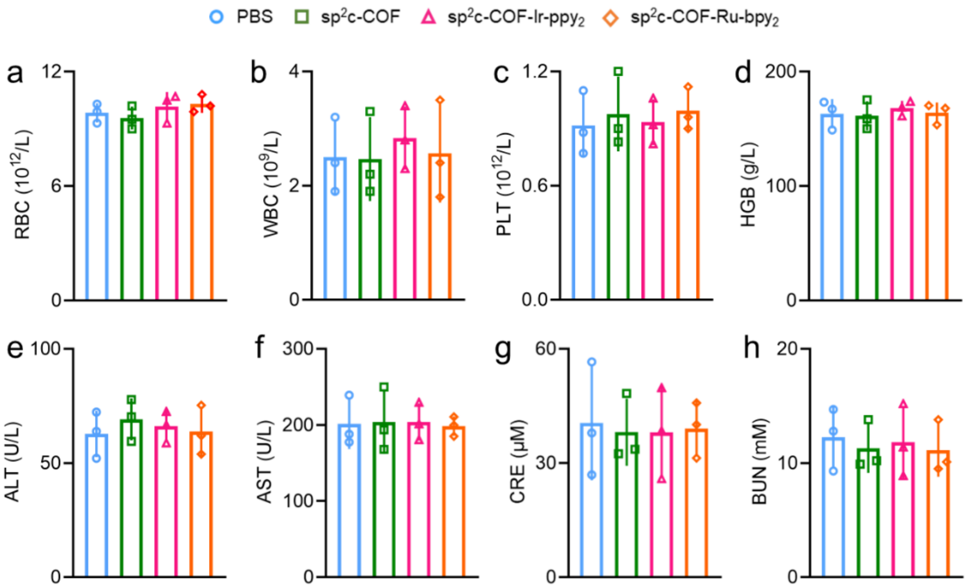

Figure S38. Hematology and clinical chemistry results for 3-day mice exposed to sp^2c-COF, sp^2c-COF-Ir-ppy$_2$, and sp^2c-COF-Ru-bpy$_2$. Data are expressed as mean ± SD; n = 3.

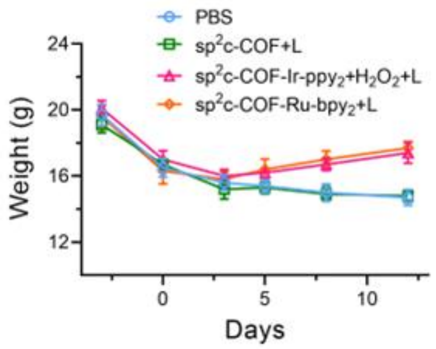

Figure S39. Body weight trajectory of the infected mice during 12-day treatments. Data are expressed as mean ± SD; n = 3.

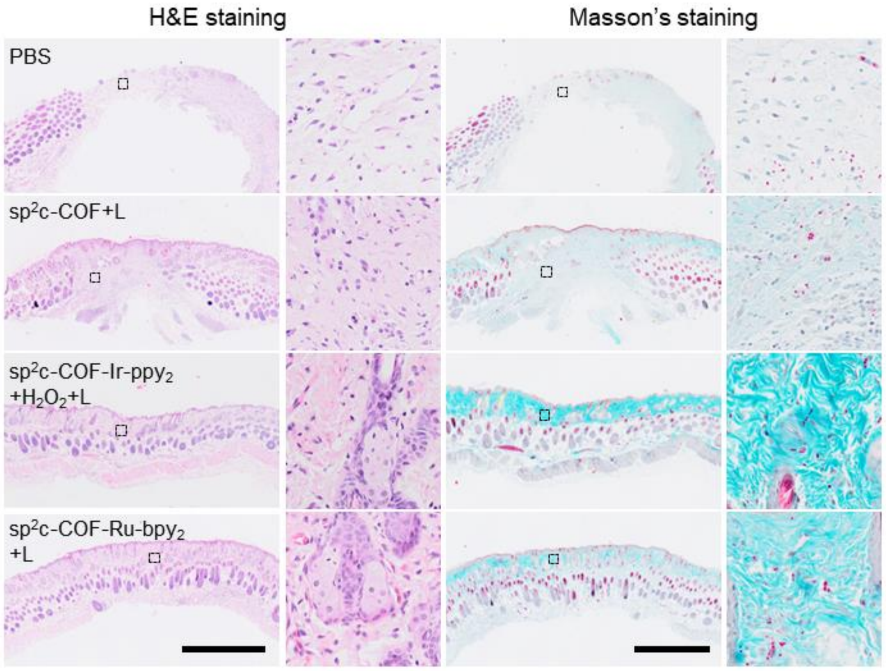

Figure S40. H&E staining and Masson's staining of the infected skin slices on day 12. Scale bar = 1 mm.

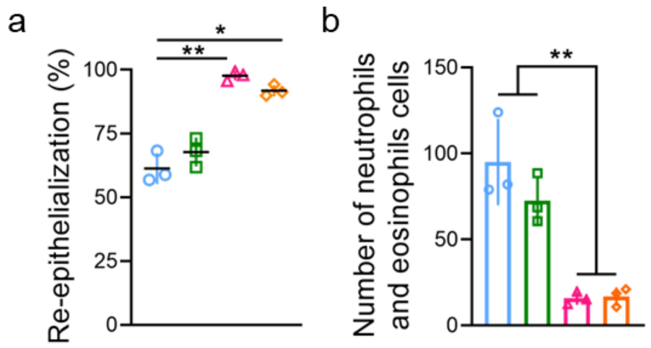

Figure S41. a) Quantification of the re-epithelialization rates. b) quantification of the number of neutrophils and eosinophils cells for Wright-stained areas. Data are expressed as a scatter plot (show

all points); n = 3. The significant differences between data were assessed by one-way ANOVA with Bonferroni's comparison test, giving *P* values, * denotes *P* < 0.05, ** denotes *P* < 0.01.

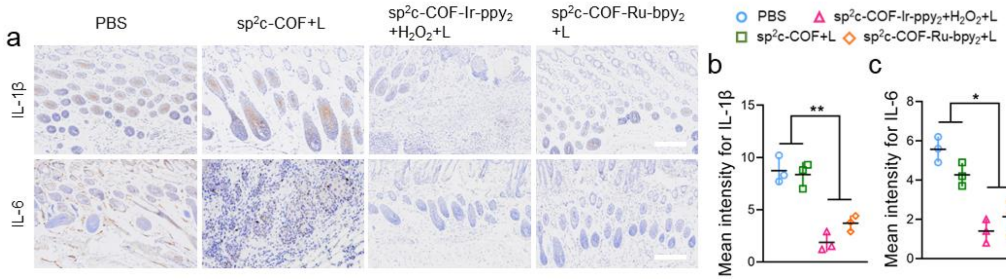

Figure S42. a) Immunohistochemical staining images of proinflammatory factors including IL-1β and IL-6 in infected wounds b-c) and quantification of the mean intensities for the areas positive for these proinflammatory factors. Scale bar = 200 μm. Data are expressed as a scatter plot (show all points); n = 3. The significant differences between data were assessed by one-way ANOVA with Bonferroni's comparison test, giving *P* values, * denotes *P* < 0.05, ** denotes *P* < 0.01.

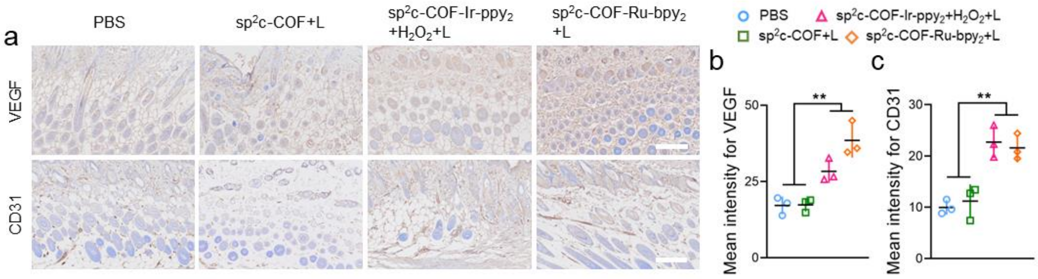

Figure S43. a) Immunohistochemical staining images of proinflammatory factors including CD31 and VEGF in infected wounds b-c) and quantification of the mean intensities for the areas positive for these proinflammatory factors. Scale bar = 200 μm. Data are expressed as a scatter plot (show all points); n = 3. The significant differences between data were assessed by one-way ANOVA with Bonferroni's comparison test, giving *P* values, * denotes *P* < 0.05, ** denotes *P* < 0.01.

Tables:

Table S1. Atomistic coordinates for the AA-stacking mode of sp^{2}c-COF optimized by DFTB+ method.

Atomic parameters						
Atom	Wyck.	Site	*x/a*	*y/b*	*z/c*	*U* [Å^2]
N1	6k	m..	0.31659	-0.31504	1/2	0
C2	6k	m..	0.29811	-0.35016	1/2	0
C3	6k	m..	0.37162	-0.26002	1/2	0
C4	6k	m..	0.24246	-0.34968	1/2	0
C5	6k	m..	0.20633	-0.36697	1/2	0
C6	6k	m..	44636	-0.40312	1/2	0
C7	6k	m..	0.20477	-0.42181	1/2	0
C8	6k	m..	0.24088	-0.40459	1/2	0

C9	6k	m..	0.43119	-0.14908	1/2	0
C10	6k	m..	44626	-0.46912	1/2	0
C11	6k	m..	0.46903	-0.5	1/2	0
C12	6k	m..	0.4682	-0.53189	1/2	0
N13	6k	m..	0.50386	-0.56049	1/2	0
C14	6k	m..	0.53619	-0.5522	1/2	0
S15	6k	m..	0.56773	-0.50588	1/2	0
C16	6k	m..	0.51979	-0.61488	1/2	0
C17	6k	m..	0.45352	-0.42112	1/2	0
C18	6k	m..	0.50169	-0.35632	1/2	0

Table S2. EXAFS fitting parameters at the Ir L_3–edge for various samples.

c	Shell	CN^a	$R(Å)^b$	$\sigma^2(Å^2)^c$	$\Delta E_0(eV)^d$	*R* factor
Ir foil	Ir-Ir	12*	2.71±0.03	0.0027	9.8	0.0022
IrO_2	Ir-O	6.1±0.9	1.98±0.04	0.0016	11.6	0.0108
**Ir SAC**	**Ir-C/N**	**6.3±0.8**	**2.02±0.02**	**0.0018**	**5.2**	**0.0055**

$^a CN$, coordination number; $^b R$, distance between absorber and backscatter atoms; $^c\sigma^2$, Debye-Waller factor to account for both thermal and structural disorders; $^d\Delta E_0$, inner potential correction; *R* factor indicates the goodness of the fit. S_0^2 was fixed to 0.72, according to the experimental EXAFS fit of Ir foil by fixing *CN* as the known crystallographic value. A reasonable range of EXAFS fitting parameters: 0.600 < S_0^2 < 1.000; *CN* > 0; σ^2 > 0 $Å^2$; $|\Delta E_0|$ < 15 eV; *R* factor < 0.02.

Table S3. EXAFS fitting parameters at the Ru K–edge for various samples.

Sample	Shell	CN^a	$R(Å)^b$	$\sigma^2(Å^2)^c$	$\Delta E_0(eV)^d$	*R* factor
Ru foil	Ru-Ru	12*	2.67±0.04	0.0010	-3.7	0.0175
RuO_2	Ru-O	5.8±0.7	1.97±0.01	0.0018	1.2	0.0067
**Ru SAC**	**Ru-C/N**	**6.1±1.0**	**2.06±0.01**	**0.0046**	**-0.7**	**0.0175**

$^a CN$, coordination number; $^b R$, distance between absorber and backscatter atoms; $^c\sigma^2$, Debye-Waller factor to account for both thermal and structural disorders; $^d\Delta E_0$, inner potential correction; *R* factor indicates the goodness of the fit. S_0^2 was fixed to 0.71, according to the experimental EXAFS fit of Ru foil by fixing *CN* as the known crystallographic value. A reasonable range of EXAFS fitting parameters: 0.600 < S_0^2 < 1.000; *CN* > 0; σ^2 > 0 $Å^2$; $|\Delta E_0|$ < 15 eV; *R* factor < 0.02.

Table S4. MICs of Different Samples against *S. aureus* and *E. coli*.

	S. aureus	*E. coli*
sp^2c-COF	> 256 μg/mL	> 256 μg/mL
sp^2c-COF-Ir-ppy_2	128 μg/mL	128 μg/mL
sp^2c-COF-Ru-bpy_2	256 μg/mL	256 μg/mL

NMR spectra:

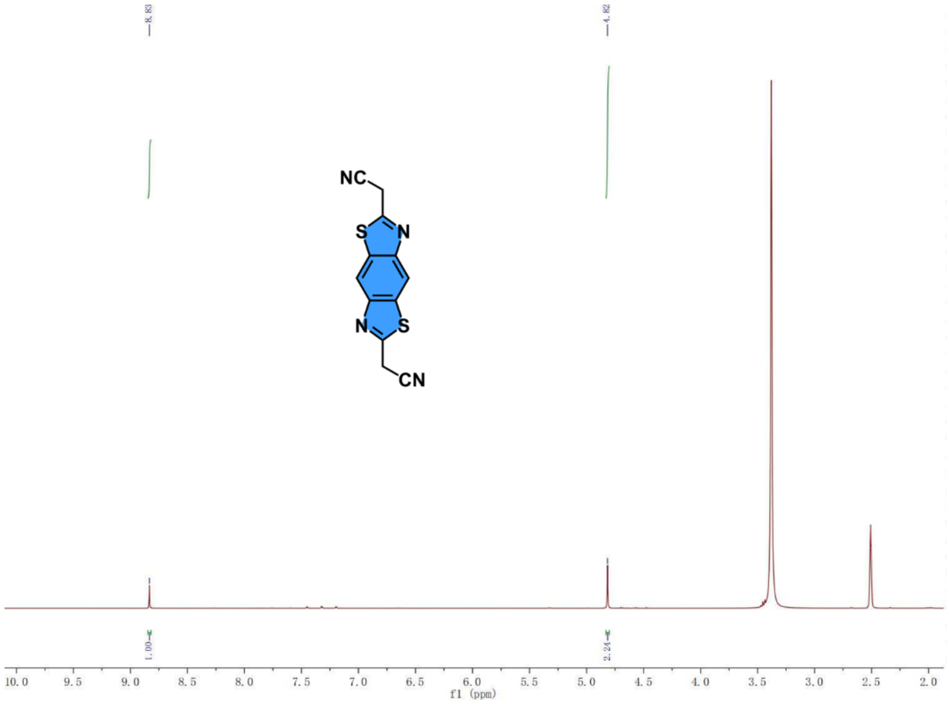

Figure S44. Liquid ^{1}H NMR spectrum of BTHAN.

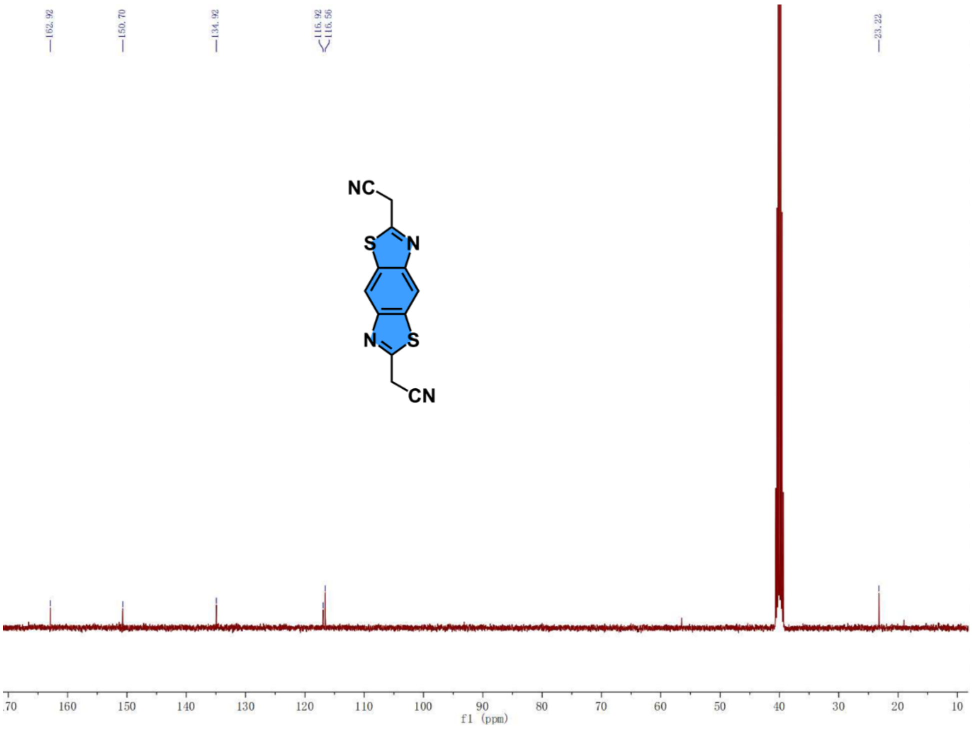

Figure S45. Liquid ^{13}C NMR spectrum of BTHAN.

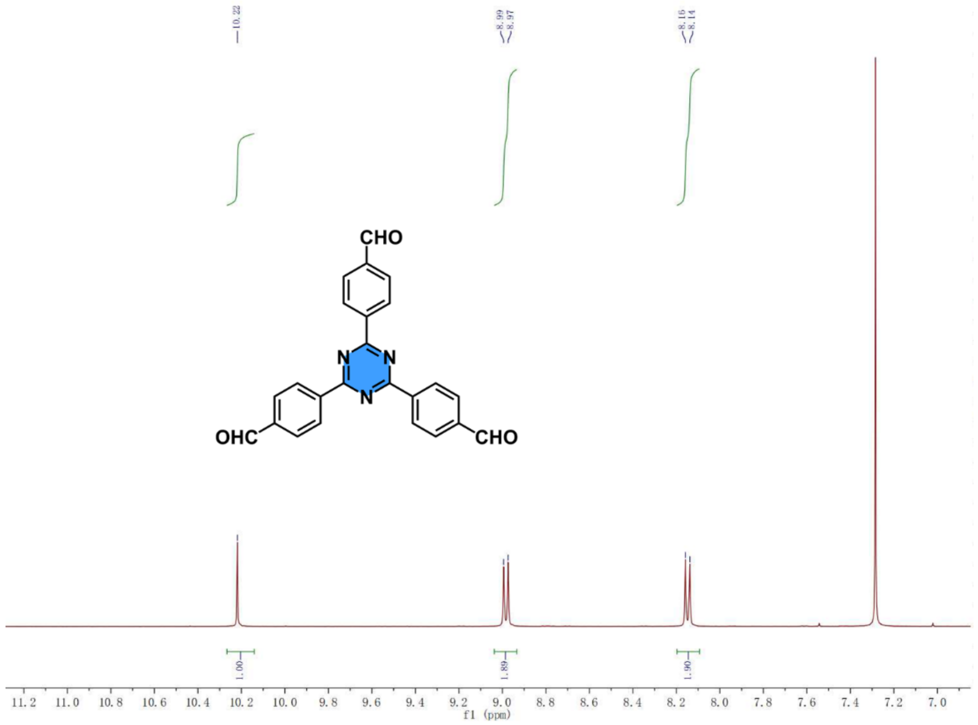

Figure S46. Liquid ^{1}H NMR spectrum of TA.

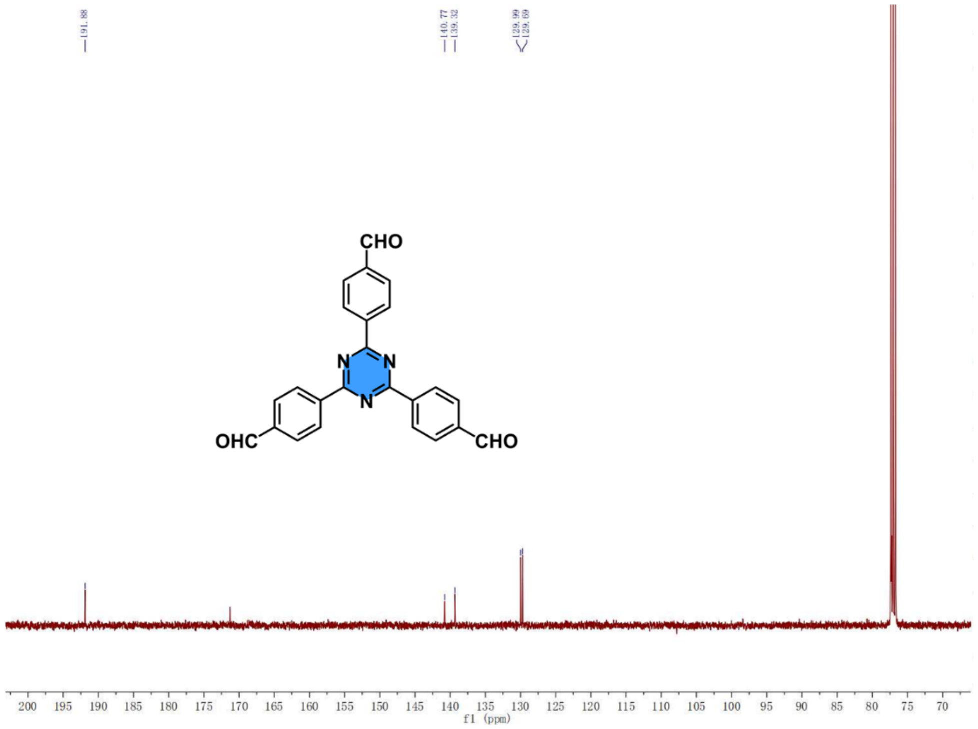

Figure S47. Liquid ^{13}C NMR spectrum of TA.

References

[1] L. Stegbauer, K. Schwinghammer, B. V. Lotsch, *Chem. Sci.* **2014**, 5, 2789.

[2] Y. Wang, W. Hao, H. Liu, R. Chen, Q. Pan, Z. Li, Y. Zhao, *Nat. Commun.* **2022**, 13, 1.

[3] A. A. Eremina, M. A. Kinzhalov, E. A. Katlenok, A. S. Smirnov, E. V. Andrusenko, E. A. Pidko, V. V.

Suslonov, K. V. Luzyanin, *Inorg. Chem.* **2020**, 59, 2209.
[4] J. J. Concepcion, J. W. Jurss, P. G. Hoertz, T. J. Meyer, *Angew. Chem., Int. Ed.* **2009**, 121, 9637.
[5] Y. Zhu, C. Xu, N. Zhang, X. Ding, B. Yu, F. J. Xu, *Adv. Funct. Mater.* **2018**, 28, 1706709.
[6] L. Gao, J. Cheng, Z. Shen, G. Zhang, S. Liu, J. Hu, *Angew. Chem., Int. Ed.* **2022**, 134, e202112782.
[7] C. Wu, Z. Liu, Z. Chen, D. Xu, L. Chen, H. Lin, J. Shi, *Sci. Adv.* **2021**, 7, eabj8833.
[8] S. He, J. Huang, Q. Zhang, W. Zhao, Z. Xu, W. Zhang, *Adv. Funct. Mater.* **2021**, 31, 2105198.
[9] C. Gong, W. Guan, X. Liu, Y. Zheng, Z. Li, Y. Zhang, S. Zhu, H. Jiang, Z. Cui, S. Wu, *Adv. Mater.* **2022**, 34, 2206134.
[10] A. M. Nash, M. I. Jarvis, S. Aghlara-Fotovat, S. Mukherjee, A. Hernandez, A. D. Hecht, P. D. Rios, S. Ghani, I. Joshi, D. Isa, *Sci. Adv.* **2022**, 8, eabm1032.
